# Evolution and Expression of Tissue Globins in Ray-Finned Fishes

**DOI:** 10.1093/gbe/evw266

**Published:** 2016-11-09

**Authors:** Michael D. Gallagher, Daniel J. Macqueen

**Affiliations:** Institute of Biological and Environmental Sciences, University of Aberdeen, Aberdeen, United Kingdom

**Keywords:** oxygen transport, ray-finned fish, globin gene family, phylogeny, evolution, gene expression

## Abstract

The globin gene family encodes oxygen-binding hemeproteins conserved across the major branches of multicellular life. The origins and evolutionary histories of complete globin repertoires have been established for many vertebrates, but there remain major knowledge gaps for ray-finned fish. Therefore, we used phylogenetic, comparative genomic and gene expression analyses to discover and characterize canonical “non-blood” globin family members (i.e., myoglobin, cytoglobin, neuroglobin, globin-X, and globin-Y) across multiple ray-finned fish lineages, revealing novel gene duplicates (paralogs) conserved from whole genome duplication (WGD) and small-scale duplication events. Our key findings were that: (1) globin-X paralogs in teleosts have been retained from the teleost-specific WGD, (2) functional paralogs of cytoglobin, neuroglobin, and globin-X, but not myoglobin, have been conserved from the salmonid-specific WGD, (3) triplicate lineage-specific myoglobin paralogs are conserved in arowanas (Osteoglossiformes), which arose by tandem duplication and diverged under positive selection, (4) globin-Y is retained in multiple early branching fish lineages that diverged before teleosts, and (5) marked variation in tissue-specific expression of globin gene repertoires exists across ray-finned fish evolution, including several previously uncharacterized sites of expression. In this respect, our data provide an interesting link between myoglobin expression and the evolution of air breathing in teleosts. Together, our findings demonstrate great-unrecognized diversity in the repertoire and expression of nonblood globins that has arisen during ray-finned fish evolution.

## Introduction

The globins are an ancient superfamily of hemeproteins that represent the most widespread oxygen-binding proteins among multicellular organisms (Hardison 1998; Weber and Vinogradov 2001; Vázquez-Limón et al. 2012; Vinogradov et al. 2013). The first discovered family members, hemoglobin (Hb) and myoglobin (Mb), are highly characterized and in vertebrates, have respective classic functions in oxygen transport and storage in blood and striated muscle, along with additional roles relating to nitric oxide (NO) and reactive oxygen species (ROS) metabolism [reviewed in Burmester and Hankeln (2014)]. More recently, multiple additional family members have been characterized that, similar to Mb, are classically considered “nonblood” or “tissue-expressed” globins [although, see Götting and Nikinmaa (2015), Corti et al. (2016a,b)], namely, neuroglobin (Ngb) (Burmester et al. 2000), cytoglobin (Cygb) (Kawada et al. 2001; Burmester et al. 2002; Trent and Hargrove 2002), Globin-X (GbX) (Roesner et al. 2005), globin-Y (GbY) (Fuchs et al. 2006), globin-E (GbE) (Kugelstadt et al. 2004), and androglobin (Adgb) (Hoogewijs et al. 2012). The functions and expression sites of these different globin genes in vertebrates are diverse and have been recently reviewed elsewhere (Burmester and Hankeln 2014).

Different globin family members are not conserved uniformly across different vertebrate lineages (Hoffman et al. 2011; Storz et al. 2011; Hoffman et al. 2012a,b; Storz et al. 2013). Thus, while some species conserve a full-range of globin types ancestral to jawed vertebrates, including coelacanths (Schwarze and Burmester 2013) and turtles (Schwarze et al. 2015), notable expansions and losses are known. For example, teleost ray-finned fish, the focus of the current study, retain functionally distinct duplicates of Cygb (Fuchs et al. 2005) and Hb alpha- and beta-type subunits (Quinn et al. 2010; Opazo et al. 2013) owing to whole genome duplication (WGD) in the teleost ancestor ∼320–350 Ma (Jaillon et al. 2004; hereafter: teleost-specific WGD, tsWGD). Moreover, salmonids have undergone further expansions in their Hb repertoire (Quinn et al. 2010), due to a further round of WGD ∼95 Ma (Macqueen and Johnston 2014; Lien et al. 2016; hereafter: salmonid-specific WGD, ssWGD). An independent WGD event in the ancestor to carp and goldfish (Cyprininae) ∼8 Ma (Xu et al. 2014) allowed the only known retention of Mb gene duplicates in ray-finned fish, which have evolved unique expression patterns and functions, potentially related to hypoxia tolerance (Fraser et al. 2006; Helbo et al. 2012). In addition to such expansions, teleosts have lost key globin genes at various times during evolution, for example, *GbY* in the lineage ancestor (Burmester and Hankeln 2014; Opazo et al. 2015). More unusual among vertebrates is the loss of *Hb* in the ancestor to the Antarctic fish family Channichthyidae [reviewed in Sidell and O’Brien (2006)] as well as losses of Mb function and/or expression in multiple lineages, including channichthyids (Sidell and O’Brien 2006), sticklebacks (Gasterosteidae), and African butterflyfish *Pantodon buchholzi* (Pantodontidae) (Macqueen et al. 2014).

Beyond these findings, there remain gaps in our understanding of the globin repertoire of several ray-finned fish lineages, including teleosts. In particular, there remain major teleost groups, where, to the best of our knowledge, globin genes remain entirely unstudied. This includes the two ray-finned fish superorders Osteoglossomorpha and Elopomorpha, which, early in teleost evolution, split from the ancestor to the superorder Clupeocephala (including lineages where globin diversity has been well-characterized, e.g., zebrafish, *Danio rerio*), creating extensive scope for divergent outcomes post-tsWGD (Martin and Holland 2014). In addition, the “nonblood” globins of salmonids are largely uncharacterized, but are likely to be maintained as gene duplicates retained from ssWGD, which is the case for over half of all genes in the genome (Lien et al. 2016), leading to expanded gene families relative to other teleosts (Macqueen et al. 2010, 2013). Finally, a systematic overview of expression of nonblood globins is yet to be achieved across the major teleost lineages in comparison to a ray-finned fish that did not undergo tsWGD, e.g., spotted gar (*Lepisosteus oculatus*) (Braasch et al. 2016). This latter work may be useful to contextualize globin roles that have evolved specifically within teleosts, including with respect to tsWGD.

Therefore, the primary objective of this study was to improve our understanding of the evolutionary diversity of nonblood/tissue globin family members in ray-finned fish, including the role played by WGD events. We employed phylogenetic, comparative genomic, and quantitative expression analyses to define and characterize full gene repertoires from a range of lineages, many previously unstudied. As a secondary objective, we included in our expression analyses two separate pairs of lineages where evolutionary losses of high cardiac Mb expression evolved independently (Macqueen et al. 2014), allowing us to explore potential impacts on the regulation of other globin family members. Our findings provide new insights into the remarkable functional-evolutionary diversity of globin genes in different fish lineages.

## Materials and Methods

### Bioinformatics

Globin gene family (protein-coding) sequences from a number of vertebrate species were obtained from NCBI (http://blast.ncbi.nlm.nih.gov/Blast.cgi), Ensembl (http://www.ensembl.org/index.html), Salmobase (http://www.salmobase.org), animalgenome.org (http://www.animalgenome.org/), and various transcriptome databases (including from: Vidotto et al. 2013; Martin and Holland 2014; Wyffels et al. 2014; Braasch et al. 2016) using the BLAST algorithm (Altschul et al. 1997). Full details of databases and accession numbers are provided in supplementary table S1, Supplementary Material online. Seventeen globin gene family member sequences used in the study that were acquired from unpublished transcriptome databases for osteoglossiform species (more details in supplementary table S1, Supplementary Material online) are provided within the supplementary material (supplementary dataset S1, Supplementary Material online). We also downloaded scaffold68 (accession number KV411197; containing three Mb paralogs) from the Asian arowana *Scleropages formosus* genome assembly (accession: ASM162426v1) (Bian et al. 2016) and used Spidey (Wheelan et al. 2001) to predict Mb intron-exon structures. Comparative analyses of synteny for genomic neighborhoods proximal to GbX genes was determined manually by inspection of assemblies from Nile tilapia *Oreochromis niloticus*, northern pike *Esox lucius*, Atlantic salmon *Salmo salar*, and spotted gar *L. oculatus* (assembly versions used provided in supplementary table S1, Supplementary Material online).

### Phylogenetic Analysis

Vertebrate globin protein sequences (*n* = 177) representing putative Mb, Ngb, GbX, Cygb, GbE, and GbY family members (identity initially assigned by BLAST), were collected and aligned using MAFFT v.7 (Katoh and Standley 2013). Adgb was not included in our study, due to its distant relationship to all other globin family members (Hoogewijs et al. 2012). The GUIDANCE2 algorithm (Sela et al. 2015) was employed to gain statistical confidence for each aligned site (overall GUIDANCE score: 0.95). The final alignment, consisting of 177 sequences and 194 aligned sites (supplementary dataset S2, Supplementary Material online), was uploaded to Mega v.6.0 (Tamura et al. 2013) and the best-fitting amino acid substitution model probabilistically determined. Phylogenetic tree building was performed using the Bayesian phylogenetic program BEAST v.1.8.2 (Drummond et al. 2012), employing the best-fitting amino acid substitution model (JTT + G: Jones et al. 1992), an uncorrelated lognormal relaxed molecular clock (Drummond et al. 2006), a Yule speciation prior (Gernhard et al. 2008), and a UPGMA tree as the start point. The BEAST analysis was run twice with a Markov chain Monte Carlo (MCMC) chain length of 25 million generations, logging the estimated parameters every 2,500 generations. Convergence and appropriate mixing of the MCMC chains were assessed using TRACER v.1.6 (http://tree.bio.ed.ac.uk/software/tracer/), where final effective samples size (ESS) values were above 200 for all sampled parameters. A maximum clade credibility (MCC) tree from one run was created using TreeAnnotator v.1.8.2 (Drummond et al. 2012), after removing the first 10% of sampled trees.

Further phylogenetic analyses were performed on *GbX*, *Mb*, and *Cygb* nucleotide protein-coding sequences, with the goal to provide better resolution to poorly resolved branching patterns (i.e., in the above phylogenetic analysis of amino acid sequences) within major globin family clades (rationale within the “Results and Discussion” section). Thus, *n* = 40, 76, and 55 respective protein-coding sequences for *GbX*, *Mb*, and *Cygb* were aligned separately using MAFFT v.7 (supplementary datasets S3–S5, Supplementary Material online, respectively). To check for the presence of substitution saturation in these alignments, which might limit accurate phylogenetic inference, we implemented the test of Xia et al. (2003) in DAMBE v.5.67 (Xia 2013). This test revealed that all three nucleotide alignments contained extensive phylogenetic signal. Specifically, in each case, comparison of the index of substitution saturation (*Iss*) to the critical Iss value (*Iss.c*) revealed that *Iss* was significantly lower than *Iss.c* under all permutations of the analysis (Xia et al. 2003). The tree-building analysis for the *GbX*, *Mb* and *Cygb* nucleotide alignments was performed in BEAST v.1.8.2, as described above, except using GTR + G+I as the best-fitting substitution model (determined in Mega v.6.0) and an MCMC chain of 100 million generations (logging estimated parameters each 10,000th generation). Analysis of the MCMC traces and generation of the MCC trees was performed as described above, again after first removing 10% of the sampled trees.

### Tests for Positive Selection on Osteoglossiform Mb

Complete protein-coding sequences of *Mb* from eleven osteoglossiform species (all sequences embedded within supplementary dataset S3, Supplementary Material online; codon alignment provided separately as supplementary dataset S6, Supplementary Material online) were manually aligned and uploaded to the Datamonkey webserver (Delport et al. 2010). Subsequently, a branch-site test was ran incorporating a random effects likelihood approach [described in Kosakovsky Pond et al. (2011)] and fixing a tree topology inferred by Bayesian phylogenetic analysis (section 2.2; tree provided in Newick format within supplementary dataset S6, Supplementary Material online). The visual output of the branch-site test provided by Datamonkey was used in the preparation of figure 3*b*.

### Animals and Tissue Sampling

We accessed previously sampled tissues from spotted gar (*n* = 4) and African butterflyfish (*n* = 5) (after: Macqueen et al. 2014) and Atlantic salmon (after: Macqueen et al. 2013). We also sampled four further species: (1) three-spined stickleback *Gasterosteus aculateus* (*n* = 5; each a pool of five individuals with mean mass: 0.35 g, SD: 0.01 g, mean length: 35 mm, SD = 3.2 mm), (2) European ruffe *Gymnocephalus cernua* (*n* = 4, mean mass: 8.3 g, SD: 2.7 g, mean length: 91 mm, SD: 8 mm), (3) Peters’ elephantnose fish *Gnathonemus petersii* (*n* = 4, mean mass: 8.8 g, SD: 2.2 g; mean length: 170 mm, SD: 9 mm), and (4) rainbow trout (*Oncorhynchus mykiss*) (*n* = 5; mean mass: 480.9 g, SD: 161.4 g; mean length: 30.9 cm; SD: 2.68 cm). Full details about the animals are provided in supplementary table S2, Supplementary Material online. Animals were acclimated in re-circulating freshwater tanks (for no less than 72 h) at the Institute of Biological and Environmental Sciences, University of Aberdeen at their optimum habitat temperature prior to Schedule-1 killing under UK Home Office guidelines. A set of tissues (including brain, heart ventricle, gill, liver, spleen, stomach, lower intestine, skin, swim bladder, and skeletal muscle) was sampled from each species, flash-frozen in liquid N_2_, and stored at −80 °C.

### Gene Expression Analysis

Total RNA was extracted from sampled tissues using TRI Reagent^®^ (Sigma-Aldrich) following the manufacturer’s instructions. RNA concentration and purity were determined using a Nano-drop 1000 system (Thermo Scientific) and by gel electrophoresis, respectively. Reverse transcription of 1 µg total RNA was performed using a Quantitect Reverse Transcription Kit (Qiagen) following the manufacturer’s instructions, including a step to remove genomic DNA. Globin gene expression was measured using quantitative polymerase chain reaction (qPCR) for first-strand cDNAs synthesized from the panel of tissue RNAs sampled from spotted gar, African butterflyfish, Peters’ elephantnose fish, Atlantic salmon, Eurasian ruffe and three-spined stickleback, using a Stratagene Mx3005P system (Agilent Technologies). We performed 15 µl reactions including 5 µl first-strand cDNA (100× dilution of stock), 7.5 µl Brilliant III Ultra-fast SYBR Green (Agilent Technologies) and 500 nM sense/antisense primers. All qPCR primers used in the study are provided in supplementary table S3, Supplementary Material online. Where possible, at least one primer in a pair was designed to cross the boundary between two exons. Cycling conditions were: 1× cycle of 3 min at 95 °C, followed by 40 cycles of 20 s at 64 °C, finishing with 30 s at 55 °C. This was followed by a DNA dissociation analysis, where single peaks were observed in all assays. All samples were run in technical duplicate, along with duplicate no-template controls (NTC) (i.e., 5 µl water in place of cDNA) and -RT controls (i.e., RNA that was not reverse-transcribed replacing cDNA). The threshold fluorescence level was set at 3,000, in the linear phase of amplification for all assays. Samples with a cycle-crossing threshold (Cq) value >35 were considered to have no expression.

Expression data from all species was analysed in Genex 5.4.3 (MultiD Analysis) and normalized to four reference genes: *ACTB* (Bower et al. 2008)*, RPS13* and *RPS29* (Macqueen et al. 2014), and *RPL8* (newly designed in this study), before being placed on a relative scale comparable across different globin genes within a species. The *RPS13, RPS29*, and *RPL8* primers were designed in regions highly conserved across a broad phylogenetic representation of ray-finned fish (Macqueen et al. 2014), incorporating a small number of degenerate sites to account for limited sequence variation across lineages. Thus, these primer pairs were explicitly designed to work in any ray-finned fish species. *ACTB* primers were not originally designed for this purpose (Bower et al. 2008), but nonetheless are highly conserved across ray-finned fish lineages.

## Results and Discussion

### Phylogenetic Analysis of Nonblood Globins Spanning Ray-Finned Fish Evolution

We performed searches for *Mb*, *Cygb*, *GbY*, *GbE*, *GbX*, and *Ngb* genes across all major jawed-vertebrate lineages, including 20 ray-finned fish species—13 previously unexamined in terms of these globins. Three of the included fish species did not experience the tsWGD, i.e., spotted gar, bowfin *Amia calva*, and Adriatic sturgeon *Acipenser naccarii*. For the included teleosts, two Anguilliform species were sampled from Elopomorpha, along with representatives from several families within Osteoglossiformes. Finally, our searches included two salmonids along with one species of Esociformes, a sister group to salmonids that never experienced ssWGD (Macqueen and Johnston 2014; Lien et al. 2016).

**Fig. 1.—:**
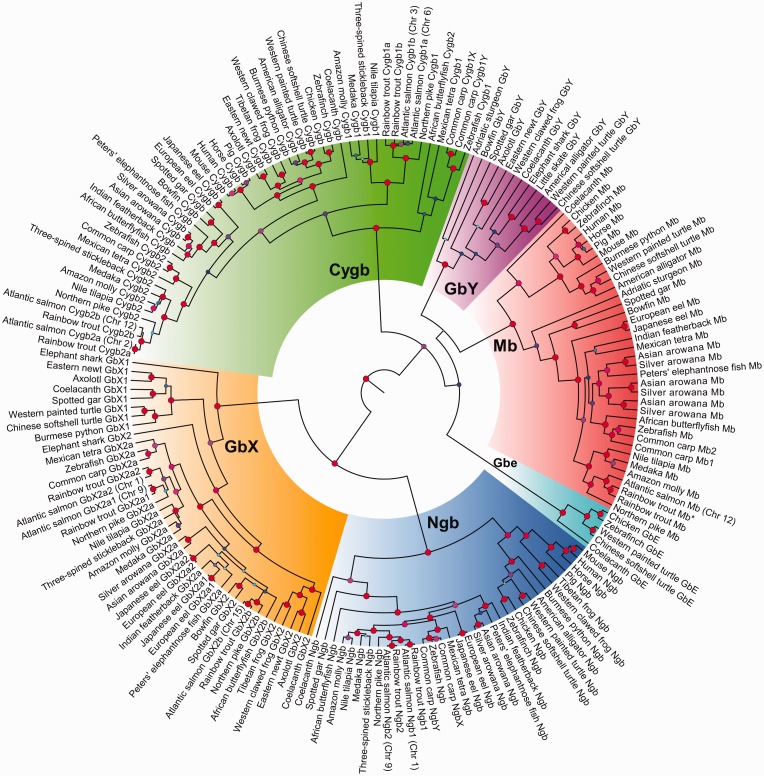
Bayesian phylogenetic analysis of “nonblood” globin gene family members spanning jawed vertebrate evolution (194 aligned amino acid sites; supplementary dataset S1, Supplementary Material online). Posterior probability support values for every reconstructed node are provided on a quantitative color scale (see provided legend in the top left corner; red = maximal support) and also indicated by the size of circles. The chromosomal locations of salmonid-specific gene duplicates are provided. Mb2* highlights a putative *Mb* pseudogene from rainbow trout that codes a truncated amino acid sequence (see main text). Accession numbers for all sequences are provided in supplementary table S1, Supplementary Material online.

A Bayesian approach incorporating a relaxed molecular clock model was used to establish phylogenetic relationships among the resultant sequences (fig. 1). The root of the tree was maximally supported (i.e., posterior probability value: 1.0) and split a monophyletic group containing Ngb and GbX from a large grouping containing separate Mb, Cygb, GbE, and GbY clades (fig. 1). While the divergence of Ngb and GbX was also maximally supported (posterior probability value: 1.0), along with the crown of all individual vertebrate globin family member clades, there was statistical uncertainty surrounding the branching arrangements of Mb, Cygb, GbE, and GbY (fig. 1). Nonetheless, the overall branching of different globin family members, including the basal position of the Ngb–GbX clade, was consistent with previous studies that did not incorporate a molecular clock model (Hoffman et al. 2011, 2012a,b; Schwarze and Burmester 2013; Opazo et al. 2015; Schwarze et al. 2015). Finally, branching patterns within each defined globin clade other than GbE (which, as previously reported, was restricted in its phylogenetic distribution; see Hoffman et al. 2011, 2012a,b; Burmester and Hankeln 2014; Schwarze et al. 2015), were indicative of novel evolutionary diversity in ray-finned fish that we go on to describe in the following sections.

### GbY Lost in Teleost Ancestor—Yet Retained during Nonteleost Fish Evolution

GbY has previously been identified in reptiles, amphibians, coelacanth, platypus and elephant shark, but not teleosts, placental mammals or birds (Fuchs et al. 2006; Patel et al. 2008; Hoffman et al. 2011; Schwarze and Burmester 2013; Burmester and Hankeln 2014; Opazo et al. 2015). Likewise, we did not identify a *GbY* gene in previously unstudied teleosts from Osteoglossiformes, Elopomorpha, or Protoacanthopterygii (i.e., northern pike and salmonids) (fig. 1). However, in all three studied nonteleost ray-finned fish (a sturgeon and two holosteans), hitherto unknown *GbY* genes were identified based on their presence within a maximally supported vertebrate GbY clade (fig. 1). Even though the branching of these early branching ray-finned fish was paraphyletic, these data offer strong support for the conservation of functional *GbY* genes across the last 400 Myr of ray-finned fish evolution (after Near et al. 2012) and a probable single ancestral loss of *GbY* in the common teleost ancestor.

### Analysis of Mb Clade Reveals Triplicate Paralogs in Arowanas

The Mb clade recovered in our main phylogenetic analysis diverged into clades for ray and lobe-finned fish with maximal posterior support (fig. 1). Within ray-finned fish, early branching lineages that did not experience tsWGD branched outside teleosts (fig. 1) as expected (Near et al. 2012; Braasch et al. 2016). Moreover, single Mb sequences from the included elopomorphs branched as a monophyletic group near the base of teleosts (fig. 1). The next group of Mb sequences split euteleosts (i.e., Acanthopterygii and Protoacanthopterygii) from a group containing Osteoglossiformes and two Ostariophysi members (fig. 1). However, African butterflyfish (Pantodontidae) branched more closely to Ostariophysi than other members of Osteoglossiformes, suggesting a branching artifact (fig. 1). Within Osteoglossiformes, there was evidence for three distinct Mb copies in two arowana species (Osteoglossidae) (fig. 1). Interestingly, while we failed to identify multiple Mb copies in other osteoglossiform lineages, a single Mb sequence from elephantnose fish (Mormyridae) branched as a sister clade to one of the arowana Mb duplicates with moderate posterior support, potentially indicating an ancestral duplication in the Osteoglossidae-Mormyridae ancestor (fig. 1).

**Fig. 2.—:**
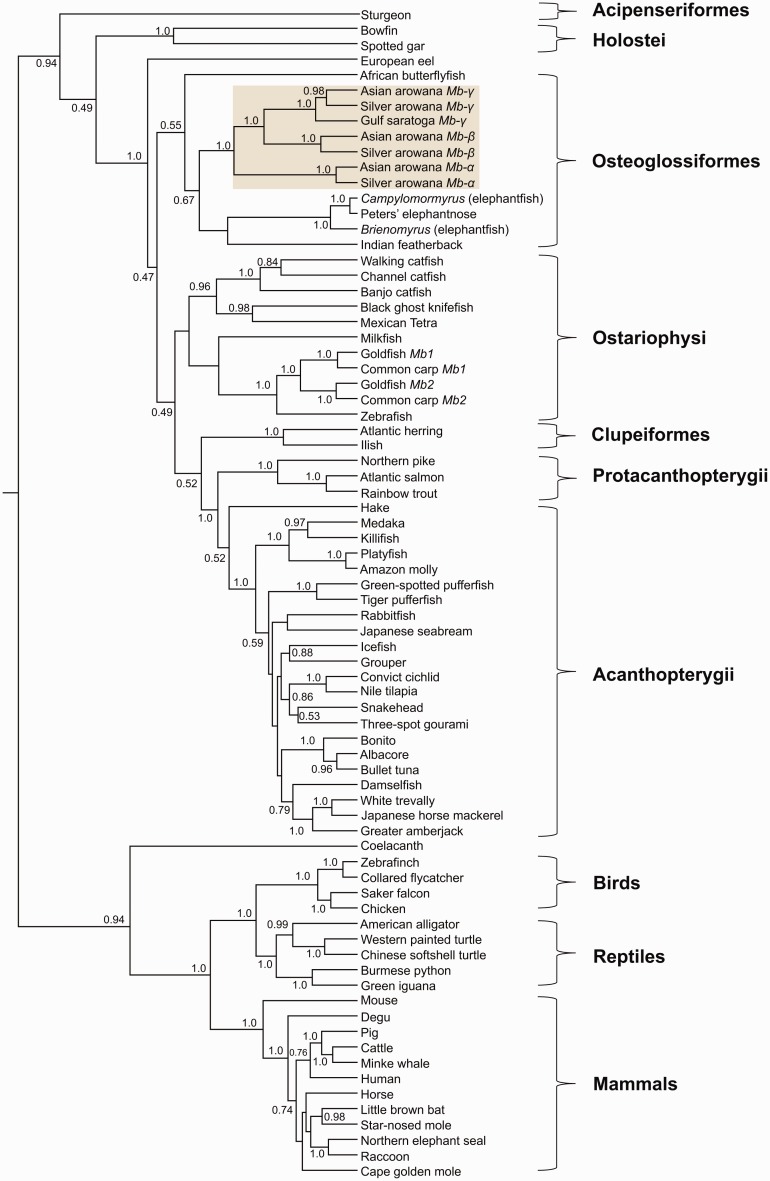
Bayesian phylogenetic analysis of vertebrate *Mb* sequences (465 aligned nucleotide sites; supplementary dataset S3, Supplementary Material online). Monophyletic clades matching to major established taxonomic groups are highlighted. For deeper reconstructed nodes in the tree, all posterior probability support values are shown. For shallower nodes, only posterior probability support values >0.5 are shown. The light brown shaded box highlights newly discovered triplicate *Mb* paralogs in the arowana lineage. Accession numbers for all included sequences are provided in supplementary table S1, Supplementary Material online.

To improve resolution around these novel features within the teleost Mb clade, we attempted to increase the available phylogenetic signal via a nucleotide-level analysis with broader representation of species, potentially offering more informative characters (fig. 2). Indeed, the resultant tree added clarity to branching patterns within teleosts, including the divergence of *Mb* paralogs within Osteoglossiformes (fig. 2). Specifically, we recovered maximal support for a clade of Mb paralogs including all the studied arowana species, which, in contrast to fig. 1, did not include Mb from other osteoglossiform lineages (fig. 2). These genes have been named *Mb-α*, *Mb-β*, and *Mb-γ* to avoid confusion with lineage-specific Mb duplicates conserved in carp and goldfish, named *Mb1* and *Mb2* (Fraser et al. 2006). The included arowanas are all from the Osteoglossinae subfamily. The crown of Osteoglossinae is represented in our analysis (i.e., divergence of silver and Asian arowana, which each retain an ortholog of the three *Mb* paralogs; fig. 2) and dates to ∼90 to 110 Ma (Lavoué 2015). Thus, the triplicate *Mb* paralogs of arowana have an ancient origin within Osteoglossidae (expanded in next section), though it remains to be established whether they exist in Arapaiminae (i.e., arapaima spp.), the other subfamily of Osteoglossidae that split from Osteoglossinae ∼140 Ma (Lavoué 2015).

In addition to the increased resolution in the phylogeny of early teleost lineages, other established ray-finned fish groups branched in their expected phylogenetic positions with respect to established species relationships (Near et al. 2012) (fig. 2), leading us to conclude that nucleotide alignments provide stronger phylogenetic signal than amino acids for considering branching patterns *within* vertebrate globin gene family members. Finally, with respect to ssWGD, we found no evidence for two salmonid-specific *Mb* paralogs in the Atlantic salmon genome (Lien et al. 2016), but evidence of two *Mb* copies in rainbow trout (fig. 1), one that has evidently been pseudogenized by a frameshift mutation deleting eight bases in the first exon and 29 bases from the second exon, leading to a truncated amino acid sequence that is unlikely to be functional.

### Origin and Evolution of Novel Mb Paralogs in Arowanas

**Fig. 3.—:**
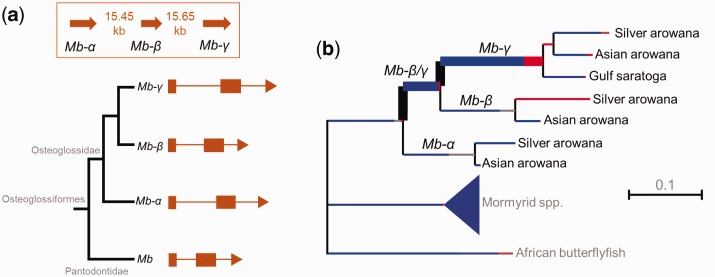
Evolution of *Mb* paralogs in the arowana lineage. (*a*) Genomic organization and intron–exon structure of tandem *Mb* duplicates in the Asian arowana genome. (*b*) Summary of the results of a branch-site test of selective pressure performed for *Mb* genes sampled across Osteoglossiformes. The thickness of each branch shows the strength of statistical support for episodic positive selection, while colors shown along branches represent the proportion of codon sites that fit into different modeled site classes (red: positive selection; blue: purifying selection; grey: neutral evolution). The mormyrid species are the same as included in figure 2. For more detailed information about the results of the branch-site test, see supplementary table S4, Supplementary Material online.

The existence of Mb paralogs in arowana is intriguing given that to date, teleost *Mb* duplicates have only been discovered in cyprinid fish (Fraser et al. 2006). Considering the broad opportunity for the generation and retention of gene duplicates in teleost evolution, for example during tsWGD and ssWGD as well as by local duplication mechanisms, along with the high frequency of retained teleost duplicates for other globin family members (e.g., this study), the frequent absence of Mb duplicates suggests it is often not advantageous (or even deleterious) to maintain more than a single *Mb* gene. We sought to gain more understanding of the circumstances surrounding the unusual retention of arowana *Mb* paralogs. Interestingly, all three genes are located in close tandem proximity within a ∼35 kb region of the Asian arowana genome (Bian et al. 2016) (fig. 3*a*). This confirms they arose by a distinct mechanism to WGD, for example, unequal crossing-over in a germline cell population. Each of the three *Mb* paralogs retains a conserved and highly compact intron–exon structure, common to *Mb* genes of most teleosts (Macqueen et al. 2014) including other osteoglossiform lineages (fig. 3*a*). In our phylogeny (fig. 2), *Mb-α* is the sister group to the clade containing *Mb-β* and *Mb-γ* and this pattern is reflected by the closer physical proximity of the two latter genes (fig. 3*a*). Thus, it is likely that an ancestral (*Mb* gene duplicated to form *Mb-α* along with a *Mb-β/γ* “protogene” that later duplicated again to form separate *Mb-β* and *Mb-*γ genes.

At the protein level, the arowana Mb paralogs share no more than 69% amino acid identity to one another, indicating high potential for functional divergence. To reconstruct the ancestral selective pressures involved, we employed a codon-based probabilistic *d*_N_/*d*_S_ test for positive selection (Kosakovsky Pond et al. 2011) along the osteoglossiform *Mb* phylogeny (fig. 3*b*, supplementary table S4, Supplementary Material online). Interestingly, there is evidence for positive selection on the ancestral branch leading into *Mb-β* and *Mb-γ* (corrected *P = *0.011), as well the ancestral *Mb-γ* branch (corrected *P = *0.026), but not along the branch leading to *Mb-α* (corrected *P = *1.0) or, with just one exception (see below), elsewhere in Osteoglossiformes (fig. 3*b*; supplementary table S4, Supplementary Material online). The branch-site test indicates that ∼20% of codons experienced positive selection on the ancestral *Mb-β* and *Mb-γ* branch in a background of strong purifying selection (fig. 3*b*; supplementary table S4, Supplementary Material online). For the ancestral Mb-γ branch, the test indicates that ∼5% of codons experienced strong positive selection in a background of moderate purifying selection (fig. 3*b*; supplementary table S4, Supplementary Material online). There was also evidence for ongoing positive selection in Silver arowana *Mb-β* (fig. 3*b*; supplementary table S4, Supplementary Material online). The only other branch inferred to be under positive selection was for the *Mb* gene of a *Campylomormyrus* elephantfish (supplementary table S4, Supplementary Material online; all other branches, corrected *P = *1.0). Together, this data suggests that the retention of *Mb* duplicates in arowana was at least partially driven by positive selection on the ancestral genes to *Mb-β* and *Mb-γ*. However, these data only represent an interesting starting point. It will be important now to establish actual functional consequences, as done for the *Mb* paralogs of common carp (Helbo et al. 2012), ultimately linking such data back to lineage-specific aspects of arowana physiology. In this respect, it will be important to test whether the arowana *Mb* paralogs are differentially expressed under situations that change demands on oxygen transport or aerobic metabolism.

### Teleosts Retain Two GbX Copies from tsWGD

**Fig. 4.—:**
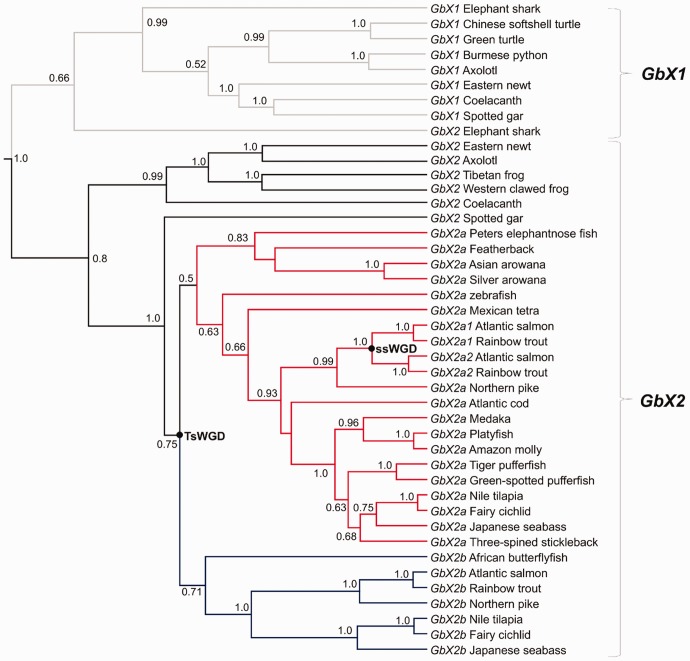
Bayesian phylogenetic analysis of vertebrate *GbX* sequences (520 aligned nucleotide sites; supplementary dataset S3, Supplementary Material online). Posterior probability support values >0.5 are shown for each reconstructed node. The position of ssWGD and tsWGD events are highlighted; putative teleost-specific *GbX* paralogs retained from tsWGD are highlighted by red (*GbX2a*) and red (*GbX2b*) branches. A supporting analysis of synteny around vertebrate *GbX* genes is provided in figure 5.

Branching patterns within the GbX clade recovered in our main phylogenetic analysis revealed a complex evolutionary history (fig. 1), consistent with a recent proposal that the common ancestor to vertebrates possessed four copies of *GbX* following WGD and/or local gene duplication events, which were differentially retained among different vertebrate lineages (Opazo et al. 2015). In our tree, ray-finned fish split into two major clades, one containing just teleosts, the other teleosts plus lineages that did not undergo tsWGD (fig. 1). However, the deep branching patterns within one of these clades were poorly statistically supported (fig. 1). Therefore, we conducted an additional phylogenetic analysis of *GbX* nucleotide sequences to gain better resolution surrounding the evolution of GbX in fish (fig. 4).

In the *GbX* phylogeny, teleosts split into two sister clades that, in turn, were sister to *GbX2* of spotted gar (fig. 4), consistent with the hypothesis that they originated during the tsWGD event. One group, named *GbX2a*, contained *GbX* genes from Osteoglossiformes, Ostariophysi, Protacanthopterygii, and Acanthopterygii, whereas the other, named *GbX2b*, contained genes from Osteoglossiformes, Protacanthopterygii, and Acanthopterygii (fig. 4). There was also evidence of divergent retention of the two putative tsWGD paralogs in different osteoglossiform groups, as African butterflyfish (Pantodontidae) branched within the *GbX2b* clade, whereas other families branched within the *GbX2a* clade (fig. 4).

**Fig. 5.—:**
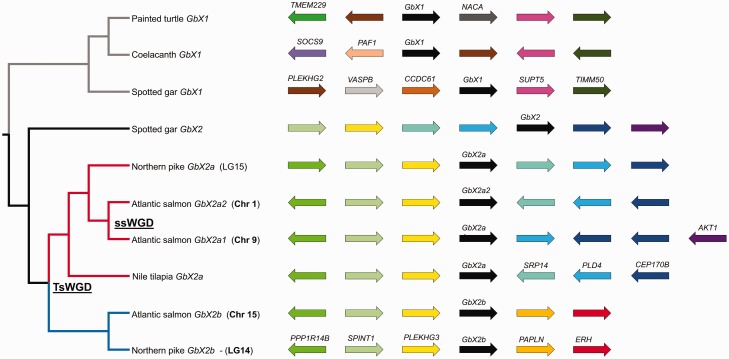
Synteny analysis of *GbX* genes from different vertebrate lineages, including *GbX2a* and *GbX2b* genes from teleosts. Each gene is shown as a colored arrow. A phylogeny is provided depicting the relationships of included *GbX* genes (after fig. 4). The position of ssWGD and tsWGD events are highlighted on this tree and putative teleost-specific *GbX* paralogs are highlighted by red (*GbX2a*) and red (*GbX2b*) branches. The chromosomal locations of *GbX* paralogs from Atlantic salmon and northern pike are provided.

To add weight to our phylogenetic findings on the origin of *GbX2a* and *GbX2b*, we examined synteny in the genomic neighborhood of these genes in comparison to *GbX1* and *GbX2* (fig. 5). *GbX1* and *GbX2* appear on different chromosomes and do not share synteny in their immediate genomic neighborhoods (fig. 5). However, *GbX1 and GbX2* orthologs from different species resided in regions of conserved synteny (fig. 5). Moreover, we observed evidence of double conserved synteny comparing *GbX2a* and *GbX2b* of teleosts with *GbX2* of spotted gar. These data also suggest that *GbX2a* and *GbX2b* arose in the common teleost ancestor, likely via the tsWGD event considering the scale of the duplicated region.

Finally, salmonid-specific paralogs of *GbX2a*, but not *GbX2b*, each coding full-length protein sequences, were recovered in our phylogenetic analyses (figs. 1 and 4). Both genes were located on different chromosomes in regions maintaining double-conserved synteny to the single *GbX2a* gene of northern pike (fig. 5), consistent with an origin through ssWGD. This conclusion is reinforced by the fact that *GbX2a1* and *a2* are each embedded within large duplicated blocks that share extensive collinearity among ssWGD paralogs (Lien et al. 2016).

### Salmonid-Specific Ngb Paralogs

In our main phylogenetic analysis, the vertebrate Ngb clade diverges into two main groupings of tetrapods and ray-finned fish, with branching patterns matching expected relationships barring small exceptions, including the presence of coelacanth within the ray-finned fish clade (fig. 1). Within the ray-finned fish group, spotted gar branches as the sister group to teleosts, as expected (fig. 1). However, notably, African butterflyfish branches outside all other teleosts (posterior probability: 1.0), with remaining Osteoglossiformes lineages forming a separate monophyletic group (fig. 1). While we did not find any evidence for two Ngb duplicates retained from tsWGD in any single species, the strong support for the position of African butterflyfish might be interpreted as the retention of the alternative Ngb tsWGD duplicate comparing this species to other teleosts.

As mentioned, our main phylogenetic analysis recovered two Ngb paralogs (tentatively called *Ngb1* and *Ngb2*; each coding full-length proteins) in salmonids, conserved in both Atlantic salmon and rainbow trout (fig. 1). Based on the fact that these duplicates branched as a sister group to Ngb of northern pike, which did not undergo ssWGD, and are located in verified duplicated ssWGD regions within the Atlantic salmon genome (Lien et al. 2016) in close proximity to *GbX2* paralogs (i.e., on Chr. 1 for *Ngb1* and Chr. 9 for *Ngb2*), we conclude their origin was via ssWGD.

### Complex Evolution of Cygb Clade in Ray-Finned Fish

While *Cygb* exists as a single gene in tetrapods, cartilaginous fish and nonteleost ray-finned fish (Hoffman et al. 2011; Storz et al. 2013), teleosts conserve two Cygb paralogs (*Cygb1* and *Cygb2*), that likely originated from tsWGD (Fuchs et al. 2005). In our analysis, while most teleost species retained two *Cygb* genes (or more in salmonids, see below), only one *Cygb* gene was recovered in elopomorphs, in addition to all osteoglossiform species barring African butterflyfish (which retains two *Cygb* genes coding full length proteins). However, in our main phylogenetic analysis, the branching of different Cygb clades did not match expectations of tsWGD (fig. 1). Specifically, several poorly supported groupings were recovered separating teleost Cygb1 and Cygb2 sequences into paraphyletic groups, with tetrapods branching as the sister to a group of ray-finned fish Cygb sequences, including both teleosts and lineages that did not experience tsWGD (fig. 1).

We performed an additional nucleotide phylogenetic analysis to gain better resolution surrounding the evolution of *Cygb* (supplementary fig. S1, Supplementary Material online). This approach was partially successful, as tetrapods and teleosts diverged as monophyletic groups and holostean fish branched as the earliest ray-finned fish clade (supplementary fig. S1, Supplementary Material online). Moreover, this tree included monophyletic *Cygb* clades for Elopomorpha and Osteoglossiformes that branched with strong support outside *Cygb1* and *Cygb2* clades largely represented by Clupeocephala lineages (supplementary fig. S1, Supplementary Material online). Taken literally, these data do not support an ancestral divergence of *Cygb1* and *Cygb2*, despite the fact that tsWGD is shared by all teleosts. One possible explanation for this finding comes from the suggestion that independent rediploidization outcomes (and hence paralog divergence) may have arisen frequently in the major different teleost subdivisions (Martin and Holland 2014). However, even this suggestion is weakened by the unexpected branching of a second *Cygb* gene from African butterflyfish within the *Cygb2* clade (supplementary fig. S1, Supplementary Material online). Overall, we conclude that the phylogenetic signal within the ray-finned fish *Cygb* clade is too weak to resolve the evolution of this globin family member with respect to tsWGD.

However, as mentioned, novel *Cygb* paralogs coding full-length proteins were identified in salmonid genomes, for both *Cygb1* (tentatively named *Cygb1a* and *Cygb1b*) *and Cygb2* (tentatively named *Cygb2a* and *Cygb2b*) as well for *Cygb1* in common carp (tentatively named *Cygb1X* and *Cygb1Y*) (supplementary fig. S1, Supplementary Material online). The distinct nomenclature is suggested to reflect the lineage-specific origins of these *Cygb* paralogs. For salmonids, the genomic locations of *Cygb1a* and *Cygb1b* (on Chr. 2 and Chr. 12, respectively) and *Cygb2a* and *Cygb2b* (on Chr. 6 and Chr. 3, respectively) are embedded within verified duplicated regions of the Atlantic salmon genome retained from ssWGD (Lien et al. 2016). Taken with their branching as respective sister groups to the single copy *Cygb1* and *Cygb2* genes of northern pike, which did not undergo ssWGD, our data suggests the retention of four unique salmonid *Cygb* genes owing to ssWGD.

### Great Diversity of Globin Family Member Expression across Ray-Finned Fish

**Fig. 6.—:**
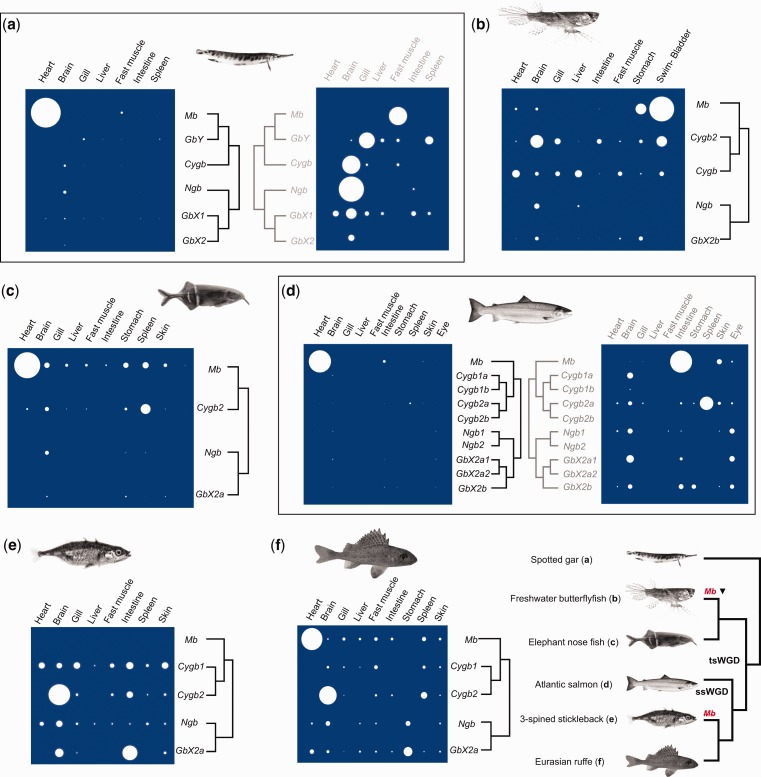
Summary of mRNA level globin family member gene expression analysis spanning ray-finned fish evolution. For each included species, across-tissue expressions of all globin genes identified in our study are shown, along with a tree to depict globin family phylogenetic relationships. The area of each circle represents the mean expression level from different biological replicates (*n* = 4 or 5 per species). Each study species is shown, along with a phylogenetic tree of all included species, in a panel at the figures bottom right. The tree also shows two independent events in evolution where the ancestral condition of high *Mb* expression in heart muscle was lost via either downregulation of mRNA-level gene expression (‘*Mb ▾’*) or by pseudogenization (*Mb*) (after Macqueen et al. 2014). For spotted gar and Atlantic salmon, data is shown with and without inclusion of *Mb* expression data in heart, due to the obscuring effect of high expression on other visualized data. Individual data for all species and genes, including means +SD is provided in supplementary figs. S2–S7, Supplementary Material online.

The final goal of our study was to characterize globin gene family member expression phenotypes spanning the evolutionary history of ray-finned fish. We used qPCR to measure mRNA expression levels of all identified nonblood globin genes across an overlapping panel of tissues sampled from six species (from four distantly related lineages) acclimated under normoxia, including spotted gar as an outgroup to tsWGD (summarized in fig. 6; data for each species provided in supplementary Figs. S2–S7, Supplementary Material online). As part of our study design, we included two pairs of lineages where the ancestral vertebrate condition of high cardiac *Mb* expression (Macqueen et al. 2014; Opazo et al. 2015) was independently lost by distinct mechanisms (fig. 6), allowing downstream impacts on the expression of other globin genes to be explored. While the measured expression levels are not directly quantitatively comparable across species, it is informative to compare (i.e., across-species) the between-tissue expression levels of different globin family members quantified within each species. However, our data are representative of entire tissues, which limits inferences at the level of specific cell types. Moreover, an inherent limitation of comparing gene expression between species is the potential for cofounding effects of ontogeny, though all species included in our study were sampled at adult stages. Despite such provisos, our study nonetheless highlights an overall remarkable diversity in tissue globin expression in different fish lineages (fig. 6), which we describe and breakdown below.

### Evolution of Globin Expression in Heart Muscle

In all species with red hearts (spotted gar, elephant nose, Atlantic salmon and European ruffe; see Macqueen et al. 2014), *Mb* was the most abundantly-expressed (by large magnitudes) globin in heart and other tissues (fig. 6; supplementary figs. S2, S4, S5, and S7, Supplementary Material online). This result is unsurprising and reflects the large mRNA population required to maintain a high abundance of oxygen-bound Mb protein in myocytes, which provides red pigmentation in heart muscle. Our data also independently confirms previous findings (Macqueen et al. 2014) that the pale-hearted African butterflyfish and three-spined stickleback have low and zero respective *Mb* mRNA levels in heart muscle (fig. 6; supplementary fig. S3 and S6, Supplementary Material online). A follow up question of interest was whether other globin family members might compensate for such losses of cardiac *Mb* expression?

In this respect, while the two pale-hearted species do not show a major upregulation of other tissue globin genes in heart (fig. 6), sticklebacks, which have lost the *Mb* gene entirely (Macqueen et al. 2014), expressed a level of *Cygb1* in heart similar to that observed in several other tissues (fig. 6*e*; supplementary fig. S6, Supplementary Material online). Conversely, in their close relative—European ruffe, which expresses high levels of cardiac *Mb*, *Cygb1* was not detectable in heart, despite being expressed in several other tissues (fig. 6*f*; supplementary fig. S7, Supplementary Material online). Given that the level of *Cygb1* heart expression in sticklebacks is minor compared with the expression of *Mb* in red-hearted species, it is unlikely to compensate for the classic role of *Mb* in facilitating oxygen transport into myocytes, as suggested following loss of *Mb* expression in Anuran (amphibian) hearts (Xi et al. 2007). Instead, the frequency with which high *Mb* expression has been lost in heart muscle during teleost evolution suggests that loss of classic *Mb* oxygen transport functions are widely tolerated among teleosts, including sticklebacks, for reasons that remain poorly characterized (Macqueen et al. 2014).

Nonetheless, it remains plausible that higher *Cygb1* expression in sticklebacks vs. red-hearted relatives compensates for loss of *Mb* enzymatic functions, for example NO decomposition/production or ROS scavenging (Flögel et al. 2001; Hendgen-Cotta et al. 2008; Helbo et al. 2012), which were lost completely at the point of *Mb* pseudogenization. Equally, such roles could be compensated for by the low observed expression of *Ngb* in stickleback hearts (fig. 6*e*; supplementary fig. S6, Supplementary Material online), which was also observed in several other species, including ruffe (fig. 6). In this respect, such enzymatic roles are a recognized function of both Cygb1 and Ngb in zebrafish (Corti et al. 2016*a*). Interestingly, the pale-hearted African butterflyfish expresses higher levels of *Cygb* in heart than *Mb* (fig. 6*b*; supplementary fig. S3, Supplementary Material online). However, this is unlikely to represent compensatory expression, given that a functional *Mb* protein-coding gene is conserved in this species (Macqueen et al. 2014) and expressed at notable levels in several tissues (fig. 6*b*; supplementary fig. S3, Supplementary Material online) (see section below). Moreover, given that that the closest relative of African butterflyfish included in our study, Peters’ elephantnose fish, also expresses low levels of *Cygb* in heart, this might simply represent an ancestral condition in Osteoglossiformes or indeed all teleosts. However, it is notable that *Cygb* expression was not detected in spotted gar heart, (fig. 6*a*; supplementary fig. S2, Supplementary Material online), a finding in common with a cartilaginous fish, the elephant shark (Opazo et al. 2015).

Clearly, future work is required to better understand the functional implications of cardiac *Mb* loss in different species. In our view, the current evidence does not support a major compensatory remodeling of expression of other globin family members in heart, which fits with the idea that this gene can be dispensable under specific physiological, ecological and environmental circumstances (Sidell and O’Brien 2006; Macqueen et al. 2014).

### Link between Mb Expression and Air-Breathing in Teleosts?

While African butterflyfish does not express high *Mb* levels in heart, it does show remarkably high levels in swim bladder—higher than any other tissue globin across all examined tissues (fig. 6*b*; supplementary fig. S3, Supplementary Material online). For illustration, the level of expression is within 1 Cq (qPCR cycle threshold) of that observed for *RPL8*, which codes a highly expressed ribosomal protein. While unconfirmed at the protein-level, it would be surprising if such high mRNA levels did not have physiological significance. This species is an obligate air-breather and uses a physostomous swim bladder as its primary respiratory organ (the ABO, or “air-breathing organ”; Graham 1997). Unfortunately, we did not originally sample the swim-bladder from the other species. Hence, to clarify if high *Mb* expression is associated with air breathing specifically, we measured the expression of *Mb* in the physostomous swim-bladder of rainbow trout, which is not used for respiration, along with other tissues for comparison (supplementary fig. S8, Supplementary Material online). In trout, *Mb* is expressed at similar levels in the swim-bladder and brain (supplementary fig. S8, Supplementary Material online), whereas in African butterflyfish the level of *Mb* in ABO is 75 times higher than in brain (supplementary fig. S3, Supplementary Material online). Moreover, the *Mb* mRNA level in trout swim-bladder is approximately 10 Cq values higher (∼1,000 times more lowly expressed) than *RPL8*, in stark contrast to African butterflyfish (see above).

The African butterflyfish fills its ABO by gulping air from the atmosphere, before gases are exchanged with blood via a thin respiratory epithelium (Graham 1997). It is possible that Mb facilitates oxygen transport (e.g., from the ABO to blood), although this is counterintuitive given that Mb binds to oxygen more strongly than Hb. Alternatively, high Mb expression in ABO may perform enzymatic functions, for example in regulating NO or ROS (Flögel et al. 2001; Hendgen-Cotta et al. 2008; Helbo et al. 2012). Clearly, understanding the potential role of *Mb* in air breathing will require additional work beyond the scope of this study.

### Evolution of Globin Expression in Brain

Our data allow us to consider the evolution of globin expression in the vertebrate brain, where several different genes are thought to have key functions (Burmester and Hankeln 2014). All studied fish expressed a diverse repertoire of brain globin mRNAs, but with notable differences among species (fig. 6). In spotted gar, *Ngb* and *Cygb* mRNA were each abundant and there was also expression of *GbX1* and *GbX2* (fig. 6*a*; supplementary fig. S2, Supplementary Material online). However, *GbY* was not detected in the gar brain (fig. 6*a*), which contrasts with the situation in elephant shark, which also expresses *Cygb* and *GbX* in brain, but lacks a *Ngb* gene, which was probably lost in a cartilaginous fish ancestor (Opazo et al. 2015).

*Ngb* was always less abundant in teleost brains (fig. 6), possibly reflecting an evolved change from the ancestral state for ray-finned fish. Nonetheless, we observed high diversity in the brain expression of different globins among teleosts (fig. 6). While *Cygb* genes were most highly expressed in Acanthopterygii species (fig. 6*e*, *f*; supplementary figs. S6 and S7, Supplementary Material online) and African butterflyfish (fig. 6*b*; supplementary fig. S3, Supplementary Material online), *GbX2a1* was most abundant in Atlantic salmon brain (fig. 6*d*; supplementary fig. S5, Supplementary Material online). In elephantnose fish, *Mb* was most abundant in brain and other tested globins were still present at relatively high levels (fig. 6*c*). Notably, in all species but elephantnose fish, *Mb* was the least expressed brain globin (fig. 6). The elephantnose belongs to Mormyridae, an osteoglossiform family that evolved an extraordinarily large brain demanding 60% of all O_2_ consumption—a value that stands far and above all other vertebrates (Nilsson 1996). Thus, the high levels of *Mb* in the elephantnose brain may facilitate O_2_ transport to mitochondria or perform enzymatic functions (e.g., to scavenge ROS). Elephantnose fish also has a high tolerance to hypoxia (Nilsson 1996), which matches our observation of high *Mb* expression across tissues beyond brain and heart (fig. 6*c*).

Further work is needed to understand the biology underlying such interspecific differences, but our data presumably reflects lineage-specific evolutionary variation in brain physiology, including differences in metabolism or hypoxia tolerance (Williams et al. 2008; Avivi et al. 2010).

### Evolution of Globin Gene Duplicate Expression

Our study, including the first complete overview of tissue-globin expression in spotted gar and Atlantic salmon, allows us to consider divergence in the expression of globin duplicates following two different WGD events. It has been demonstrated elsewhere that *Cygb* paralogs of zebrafish are differentially expressed, with *Cygb2* being more abundant in neural tissues (Fuchs et al. 2005). This differential expression pattern is observed in several species within our study, including from Acanthopterygii (fig. 6*e* and *f*; supplementary fig. S6 and S7, Supplementary Material online) and the African butterflyfish (fig. 6*b*; supplementary fig. S3, Supplementary Material online) and may have evolved in the teleost ancestor. However, in comparison to spotted gar, most teleosts express both *Cygb* duplicates more broadly across tissues (fig. 6), except for Atlantic salmon, where both *Cygb1* genes are restricted to brain (fig. 6*d*). This is also the case for teleost-specific duplicates of *GbX2*. Additionally, while in spotted gar, the single copy *GbX2* gene is lowly expressed under normoxia and brain-restricted, in teleosts, *GbX2* genes tend to be more broadly expressed across tissues (fig. 6). Interestingly, in Atlantic salmon, the only tested species retaining *GbX2* duplicates from tsWGD, there was evidence of divergent expression among tsWGD paralogs (fig. 6*d*). Specifically, while *GbX2a* and *GbX2b* had overlapping expression domains in tissues, *GbX2b* was expressed in additional tissues relative to its paralog (fig. 6*d*).

In Atlantic salmon, there was also evidence for either quantitative divergence in expression of salmonid-specific paralogs restricted to the same tissues (i.e., *Cygb1a* vs. *Cygb1b*), or extreme asymmetric divergence in expression (i.e. *Cygb2a* vs. *Cygb2b, GbX2a1* vs. *GbX2a2*, and *Ngb1* vs. *Ngb2*) with one paralog broadly expressed across tissues and the other undetected (fig. 6*d*). However, each salmonid paralog that was not expressed (i.e., *Cygb2b, GbX2a2*, and *Ngb2*) codes a full-length protein (evidenced in supplementary dataset S2, Supplementary Material online), have complete intron–exon structures (not shown) and are also retained in rainbow trout (fig. 1). Therefore, they are evidently functional genes that have evolved specialized expression patterns, perhaps restricted to tissues/cells outside our investigation, or only induced under specific physiological conditions.

Clearly, more work will be required to better understand the functional divergence of teleost globin genes beyond this rudimentary tissue expression data. In this respect, we feel the recent discovery that zebrafish GbX plays an important role in NO generation in red blood cells (Corti et al. 2016b) will be particularly worthy of further investigation with respect to the three duplicated copies of *GbX2* retained in salmonid fish, where circulatory physiology is of great current interest.

### GbY Expression in Spotted Gar

Finally, it is worth briefly comparing the expression of *GbY* in spotted gar with previous reports in elephant shark (Opazo et al. 2015), which may provide clues into the early evolution of this gene’s functional role. We found the highest levels of *GbY* expression in spotted gar gill, followed by spleen (fig. 6*a*). However, in elephant shark, *GbY* expression was highest in intestine (a site where *GbY* was not expressed in spotted gar; fig. 6*a*) and found at much lower levels in spleen and gill (Opazo et al. 2015). While the relevance of these differences among lineages remains unclear, this data adds knowledge to a highly limited body of evidence concerning the functional role of *GbY* in vertebrates (Burmester and Hankeln 2014).

## Conclusions

We have successfully demonstrated novel evolutionary diversity within the globin gene family of teleosts, including with respect to gene duplication and WGD events. We also provided evidence for a number of novel globin tissue expression sites. We hope that our findings and broadly encompassing expression data will stimulate ongoing attempts to characterize how diverse globin gene repertoires and tissue expression patterns have contributed to the unmatched natural physiological diversity of fishes.

## Supplementary Material

Supplementary data are available at *Genome Biology and Evolution* online.

## Author Contributions

Designed research: DJM. Performed experiments: MDG. Both authors analyzed and interpreted data, designed figures/tables and wrote the manuscript.

## Supplementary Material

Supplementary Data

## References

[evw266-B1] Altschul SF, 1997 Gapped BLAST and PSI-BLAST: a new generation of protein database search programs. Nucleic Acids Res. 25:3389–3402. 9254694 10.1093/nar/25.17.3389 PMC146917

[evw266-B2] Avivi A, 2010 Neuroglobin, cytoglobin, and myoglobin contribute to hypoxia adaptation of the subterranean mole rat *Spalax* . Proc Natl Acad Sci U S A. 107:21570–25175. 21115824 10.1073/pnas.1015379107 PMC3003035

[evw266-B3] Bian C, 2016 The Asian arowana (*Scleropages formosus*) genome provides new insights into the evolution of an early lineage of teleosts. Sci Rep. 6:24501. 27089831 10.1038/srep24501 PMC4835728

[evw266-B4] Bower NI Li X Taylor R Johnston IA. 2008 Switching to fast growth: the insulin-like growth factor (IGF) system in skeletal muscle of Atlantic salmon. J Exp Biol. 211:3859–3870. 19043058 10.1242/jeb.024117

[evw266-B5] Braasch I, 2016 The spotted gar genome illuminates vertebrate evolution and facilitates human-teleost comparisons. Nat Genet. 48:427–437. 26950095 10.1038/ng.3526 PMC4817229

[evw266-B6] Burmester T Weich B Reinhardt S Hankeln T. 2000 A vertebrate globin expressed in the brain. Nature 407:520–523. 11029004 10.1038/35035093

[evw266-B7] Burmester T Ebner B Weich B Hankeln T. 2002 Cytoglobin: a novel globin type ubiquitously expressed in vertebrate tissues. Mol Biol Evol. 19:416–421. 11919282 10.1093/oxfordjournals.molbev.a004096

[evw266-B8] Burmester T Hankeln T. 2014 Function and evolution of vertebrate globins. Acta Physiol. 211:501–514. 10.1111/apha.12312 24811692

[evw266-B9] Corti P Ieraci M Tejero J. 2016a Characterization of zebrafish neuroglobin and cytoglobins 1 and 2: zebrafish cytoglobins provide insights into the transition from six-coordinate to five-coordinate globins. Nitric Oxide 53:22–34. 26721561 10.1016/j.niox.2015.12.004

[evw266-B10] Corti P, 2016b Globin X is a six-coordinate globin that reduces nitrite to nitric oxide in fish red blood cells. Proc Natl Acad Sci U S A. 113:8538–8543. 27407144 10.1073/pnas.1522670113 PMC4968712

[evw266-B11] Delport W Poon AF Frost SD Kosakovsky Pond SL. 2010 Datamonkey 2010: a suite of phylogenetic analysis tools for evolutionary biology. Bioinformatics 26:2455–2457. 20671151 10.1093/bioinformatics/btq429 PMC2944195

[evw266-B12] Drummond AJ Ho SY Phillips MJ Rambaut A. 2006 Relaxed phylogenetics and dating with confidence. PLoS Biol. 4:e88. 16683862 10.1371/journal.pbio.0040088 PMC1395354

[evw266-B13] Drummond AJ Suchard MA Xie D Rambaut A. 2012 Bayesian phylogenetics with BEAUti and the BEAST 17. Mol Biol Evol. 29:969–1973. 10.1093/molbev/mss075 PMC3408070 22367748

[evw266-B14] Flögel U Merx MW Gödecke A Decking UK Schrader J. 2001 Myoglobin: a scavenger of bioactive NO. Proc Natl Acad Sci U S A. 98:735–740. 11136228 10.1073/pnas.011460298 PMC14657

[evw266-B15] Fraser J, 2006 Hypoxia-inducible myoglobin expression in nonmuscle tissues. Proc Natl Acad Sci U S A. 103:2977–2981. 16469844 10.1073/pnas.0508270103 PMC1413783

[evw266-B16] Fuchs C Luckhardt A Gerlach F Burmester T Hankeln T. 2005 Duplicated cytoglobin genes in teleost fishes. Biochem Biophys Res Commun. 337:216–223. 16199220 10.1016/j.bbrc.2005.08.271

[evw266-B17] Fuchs C Burmester T Hankeln T. 2006 The amphibian globin gene repertoire as revealed by the *Xenopus* genome. Cytogenet Genome Res. 112:296–306. 16484786 10.1159/000089884

[evw266-B18] Gernhard T Hartmann K Steel M. 2008 Stochastic properties of generalised Yule models, with biodiversity applications. J Math Biol. 57:713–735. 18509650 10.1007/s00285-008-0186-y

[evw266-B19] Götting M Nikinmaa M. 2015 More than hemoglobin—the unexpected diversity of globins in vertebrate red blood cells. Physiol Rep. 3:e12284. 25649247 10.14814/phy2.12284 PMC4393193

[evw266-B20] Graham JB. 1997 Air-breathing fishes: evolution, diversity, and adaptation. New York: Academic Press.

[evw266-B21] Hardison R. 1998 Hemoglobins from bacteria to man: evolution of different patterns of gene expression. J Exp Biol. 201:1099–1117. 9510523 10.1242/jeb.201.8.1099

[evw266-B22] Helbo S, 2012 Functional differentiation of myoglobin isoforms in hypoxia-tolerant carp indicates tissue-specific protective roles. Am J Physiol Regul Integr Comp Physiol. 302:R693–R701. 22170621 10.1152/ajpregu.00501.2011

[evw266-B23] Hendgen-Cotta UB, 2008 Nitrite reductase activity of myoglobin regulates respiration and cellular viability in myocardial ischemia-reperfusion injury. Proc Natl Acad Sci U S A. 105:10256–10261. 18632562 10.1073/pnas.0801336105 PMC2481313

[evw266-B24] Hoffmann FG Opazo JC Storz JF. 2011 Differential loss and retention of cytoglobin, myoglobin, and globin-E during the radiation of vertebrates. Genome Biol Evol. 3:588–600. 21697098 10.1093/gbe/evr055 PMC3156568

[evw266-B25] Hoffmann FG, 2012a Evolution of the globin gene family in deuterostomes: lineage-specific patterns of diversification and attrition. Mol Biol Evol. 29:1735–1745. 22319164 10.1093/molbev/mss018 PMC3375472

[evw266-B26] Hoffmann FG Opazo JC Storz JF. 2012b Whole-genome duplications spurred the functional diversification of the globin gene superfamily in vertebrates. Mol Biol Evol. 29:303–312. 21965344 10.1093/molbev/msr207 PMC3245541

[evw266-B27] Hoogewijs D, 2012 Androglobin: a chimeric globin in metazoans that is preferentially expressed in Mammalian testes. Mol Biol Evol. 29:1105–1114. 22115833 10.1093/molbev/msr246 PMC3350324

[evw266-B28] Jaillon O, 2004 Genome duplication in the teleost fish *Tetraodon nigroviridis* reveals the early vertebrate proto-karyotype. Nature 431:946–957. 15496914 10.1038/nature03025

[evw266-B29] Jones DT Taylor WR Thornton JM. 1992 The rapid generation of mutation data matrices from protein sequences. Comput Appl Biosci. 8:275–282. 1633570 10.1093/bioinformatics/8.3.275

[evw266-B30] Katoh K Standley DM. 2013 MAFFT multiple sequence alignment software version 7: improvements in performance and usability. Mol Biol Evol. 30:772–780. 23329690 10.1093/molbev/mst010 PMC3603318

[evw266-B31] Kawada N, 2001 Characterization of a stellate cell activation-associated protein (STAP) with peroxidase activity found in rat hepatic stellate cells. J Biol Chem. 276:25318–25323. 11320098 10.1074/jbc.M102630200

[evw266-B32] Kosakovsky Pond SLK, 2011 A random effects branch-site model for detecting episodic diversifying selection. Mol Biol Evol. 28:3033–3043. 21670087 10.1093/molbev/msr125 PMC3247808

[evw266-B33] Kugelstadt D Haberkamp M Hankeln T Burmester T. 2004 Neuroglobin, cytoglobin, and a novel, eye-specific globin from chicken. Biochem Biophys Res Commun. 325:719–725. 15541349 10.1016/j.bbrc.2004.10.080

[evw266-B34] Lavoué S. 2015 Testing a time hypothesis in the biogeography of the arowana genus *Scleropages* (Osteoglossidae). J Biogeogr. 42:2427–2439.

[evw266-B35] Lien S, 2016 The Atlantic salmon genome provides insights into rediploidization. Nature 533:200–205. 27088604 10.1038/nature17164 PMC8127823

[evw266-B36] Macqueen DJ Kristjánsson BK Johnston IA. 2010 Salmonid genomes have a remarkably expanded akirin family, coexpressed with genes from conserved pathways governing skeletal muscle growth and catabolism. Physiol Genomics 42:134–148. 20388840 10.1152/physiolgenomics.00045.2010 PMC2888561

[evw266-B37] Macqueen DJ Garcia de la Serrana D Johnston IA. 2013 Evolution of ancient functions in the vertebrate insulin-like growth factor system uncovered by study of duplicated salmonid fish genomes. Mol Biol Evol. 30:1060–1076. 23360665 10.1093/molbev/mst017 PMC3670735

[evw266-B38] Macqueen DJ Johnston IA. 2014 A well-constrained estimate for the timing of the salmonid whole genome duplication reveals major decoupling from species diversification. Proc Biol Sci. 281:20132881. 24452024 10.1098/rspb.2013.2881 PMC3906940

[evw266-B39] Macqueen DJ Garcia de la Serrana D Johnston IA. 2014 Cardiac myoglobin deficit has evolved repeatedly in teleost fishes. Biol Lett. 10:20140225. 24919701 10.1098/rsbl.2014.0225 PMC4090546

[evw266-B40] Martin KJ Holland PW. 2014 Enigmatic orthology relationships between Hox clusters of the African butterfly fish and other teleosts following ancient whole-genome duplication. Mol Biol Evol. 31:2592–2611. 24974377 10.1093/molbev/msu202 PMC4166920

[evw266-B41] Near TJ, 2012 Resolution of ray-finned fish phylogeny and timing of diversification. Proc Natl Acad Sci U S A. 109:13698–13703. 22869754 10.1073/pnas.1206625109 PMC3427055

[evw266-B42] Nilsson G. 1996 Brain and body oxygen requirements of *Gnathonemus petersii*, a fish with an exceptionally large brain. J Exp Biol. 199:603–607. 9318319 10.1242/jeb.199.3.603

[evw266-B43] Opazo JC Butts GT Nery MF Storz JF Hoffmann FG. 2013 Whole-genome duplication and the functional diversification of teleost fish hemoglobins. Mol Biol Evol. 30:140–153. 22949522 10.1093/molbev/mss212 PMC3525417

[evw266-B44] Opazo JC, 2015 Ancient duplications and expression divergence in the globin gene superfamily of vertebrates: insights from the elephant shark genome and transcriptome. Mol Biol Evol. 32:1684–1694. 25743544 10.1093/molbev/msv054 PMC4476154

[evw266-B66] Patel VS Cooper SJ Deakin JE Fulton B Graves T Warren WC Wilson RK Graves JA 2008 Platypus globin genes and flanking loci suggest a new insertional model for beta-globin evolution in birds and mammals. BMC Biol. 6:12-20. 18657265 10.1186/1741-7007-6-34 PMC2529266

[evw266-B45] Quinn NL, 2010 Genomic organization and evolution of the Atlantic salmon hemoglobin repertoire. BMC Genomics 11:539. 20923558 10.1186/1471-2164-11-539 PMC3091688

[evw266-B46] Roesner A Fuchs C Hankeln T Burmester T. 2005 A globin gene of ancient evolutionary origin in lower vertebrates: evidence for two distinct globin families in animals. Mol Biol Evol. 22:12–20. 15356282 10.1093/molbev/msh258

[evw266-B47] Schwarze K Burmester T. 2013 Conservation of globin genes in the “living fossil” *Latimeria chalumnae* and reconstruction of the evolution of the vertebrate globin family. Biochim Biophys Acta. 1834:1801–1812. 23360762 10.1016/j.bbapap.2013.01.019

[evw266-B48] Schwarze K Singh A Burmester T. 2015 The full globin repertoire of turtles provides insights into vertebrate globin evolution and functions. Genom Biol Evol. 7:1896–1913. 10.1093/gbe/evv114 PMC4524481 26078264

[evw266-B49] Sela I Ashkenazy H Katoh K Pupko T. 2015 GUIDANCE2: accurate detection of unreliable alignment regions accounting for the uncertainty of multiple parameters. Nucleic Acids Res. 43:W7–W14. 25883146 10.1093/nar/gkv318 PMC4489236

[evw266-B50] Sidell BD O'Brien KM. 2006 When bad things happen to good fish: the loss of hemoglobin and myoglobin expression in Antarctic icefishes. J Exp Biol. 209:1791–1802. 16651546 10.1242/jeb.02091

[evw266-B51] Storz JF Opazo JC Hoffmann FG. 2011 Phylogenetic diversification of the globin gene superfamily in chordates. IUBMB Life 63:313–322. 21557448 10.1002/iub.482 PMC4399706

[evw266-B52] Storz JF Opazo JC Hoffmann FG. 2013 Gene duplication, genome duplication, and the functional diversification of vertebrate globins. Mol Phylogenet Evol. 66:469–478. 22846683 10.1016/j.ympev.2012.07.013 PMC4306229

[evw266-B53] Tamura K Stecher G Peterson D Filipski A Kumar S. 2013 MEGA6: molecular evolutionary genetics analysis version 6.0. Mol Biol Evol. 30:2725–2729. 24132122 10.1093/molbev/mst197 PMC3840312

[evw266-B54] Trent JT Hargrove MS. 2002 A ubiquitously expressed human hexacoordinate hemoglobin. J Biol Chem. 277:19538–19545. 11893755 10.1074/jbc.M201934200

[evw266-B55] Vázquez-Limón C Hoogewijs D Vinogradov SN Arredondo-Peter R. 2012 The evolution of land plant hemoglobins. Plant Sci. 191:71–81. 22682566 10.1016/j.plantsci.2012.04.013

[evw266-B56] Vidotto M, 2013 Transcriptome sequencing and de novo annotation of the critically endangered Adriatic sturgeon. BMC Genomics 7:1008–1025. 10.1186/1471-2164-14-407 PMC3691660 23773438

[evw266-B57] Vinogradov SN Tinajero-Trejo M Poole RK Hoogewijs D. 2013 Bacterial and archaeal globins—a revised perspective. Biochim Biophys Acta. 1834:1789–1800. 23541529 10.1016/j.bbapap.2013.03.021

[evw266-B58] Weber RE Vinogradov SN. 2001 Nonvertebrate hemoglobins: functions and molecular adaptations. Physiol Rev. 81:569–628. 11274340 10.1152/physrev.2001.81.2.569

[evw266-B59] Wheelan SJ Church DM Ostell JM. 2001 Spidey: a tool for mRNA-to-genomic alignments. Genome Res. 11:1952–1957. 11691860 10.1101/gr.195301 PMC311166

[evw266-B60] Williams TM, 2008 Running, swimming and diving modifies neuroprotecting globins in the mammalian brain. Proc Biol Sci. 275:751–758. 18089537 10.1098/rspb.2007.1484 PMC2596902

[evw266-B61] Wyffels J King BL Vincent J Chen C Wu CH Polson SW. 2014 SkateBase, an elasmobranch genome project and collection of molecular resources for chondrichthyan fishes. F1000Res. 12:191. 10.12688/f1000research.4996.1 PMC4184313 25309735

[evw266-B62] Xia X. 2013 DAMBE5: a comprehensive software package for data analysis in molecular biology and evolution. Mol Biol Evol. 30:1720–1728. 23564938 10.1093/molbev/mst064 PMC3684854

[evw266-B63] Xia X Xie Z Salemi M Chen L Wang Y. 2003 An index of substitution saturation and its application. Mol Phylogenet Evol. 26:1–7. 12470932 10.1016/s1055-7903(02)00326-3

[evw266-B64] Xi Y Obara M Ishida Y Ikeda S Yoshizato K. 2007 Gene expression and tissue distribution of cytoglobin and myoglobin in the Amphibia and Reptilia: possible compensation of myoglobin with cytoglobin in skeletal muscle cells of anurans that lack the myoglobin gene. Gene 398:94–102. 17560742 10.1016/j.gene.2007.01.040

[evw266-B65] Xu P, 2014 Genome sequence and genetic diversity of the common carp, *Cyprinus carpio* . Nat Genet. 46:1212–1219. 25240282 10.1038/ng.3098

