# Supplementary material for: Evolution and Expression of Tissue Globins in Ray-Finned Fishes
Source: Genome Biol Evol. 2016 Nov 9;9(1):32–47. doi: 10.1093/gbe/evw266 (PMC5381549; doi:10.1093/gbe/evw266)
Supplement: Supplementary Data [file evw266_Supp.zip › GBE-160801-R1-Supplemental_Information.pdf]

Supplemental Information

Evolution and expression of tissue globins in ray-finned fishes

Michael D. Gallagher, Daniel J. Macqueen

Contains:

Fig. S1, S2, S3, S4, S5, S6, S7 and S8.

Tables S2, S3 and S4.

Dataset S1.

Additionally, Table S1 and Datasets S2, S3, S4, S5 and S6 are attached to this paper as further online supplementary material that is contained out-with this document.

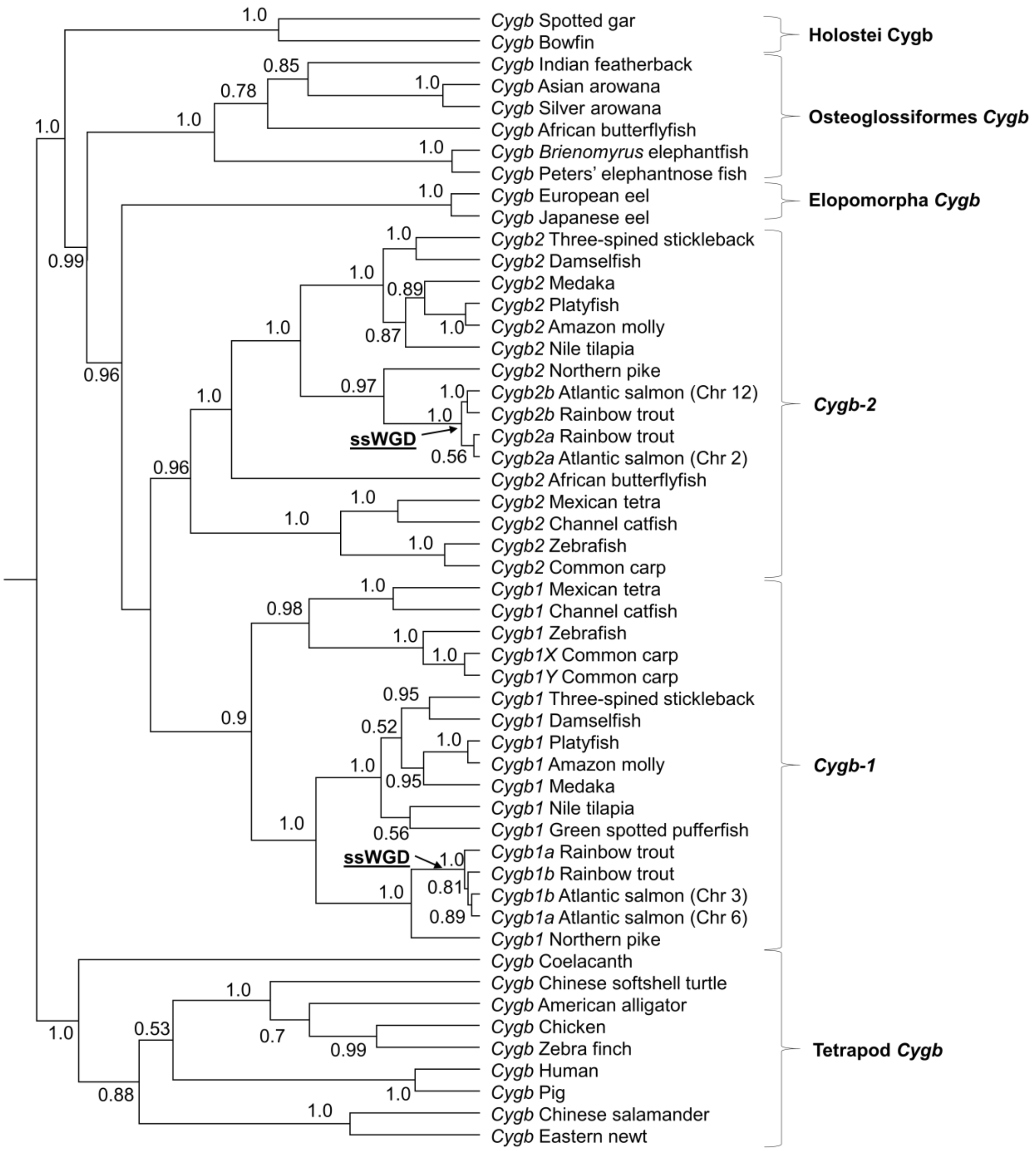

Fig. S1. Bayesian phylogenetic analysis of vertebrate *Cygb* sequences (512 aligned nucleotide sites; Dataset S5). Taxonomic groupings are shown to demonstrate monophyletic branching of distinct clades. Posterior probability support >0.5 is shown at reconstructed nodes.

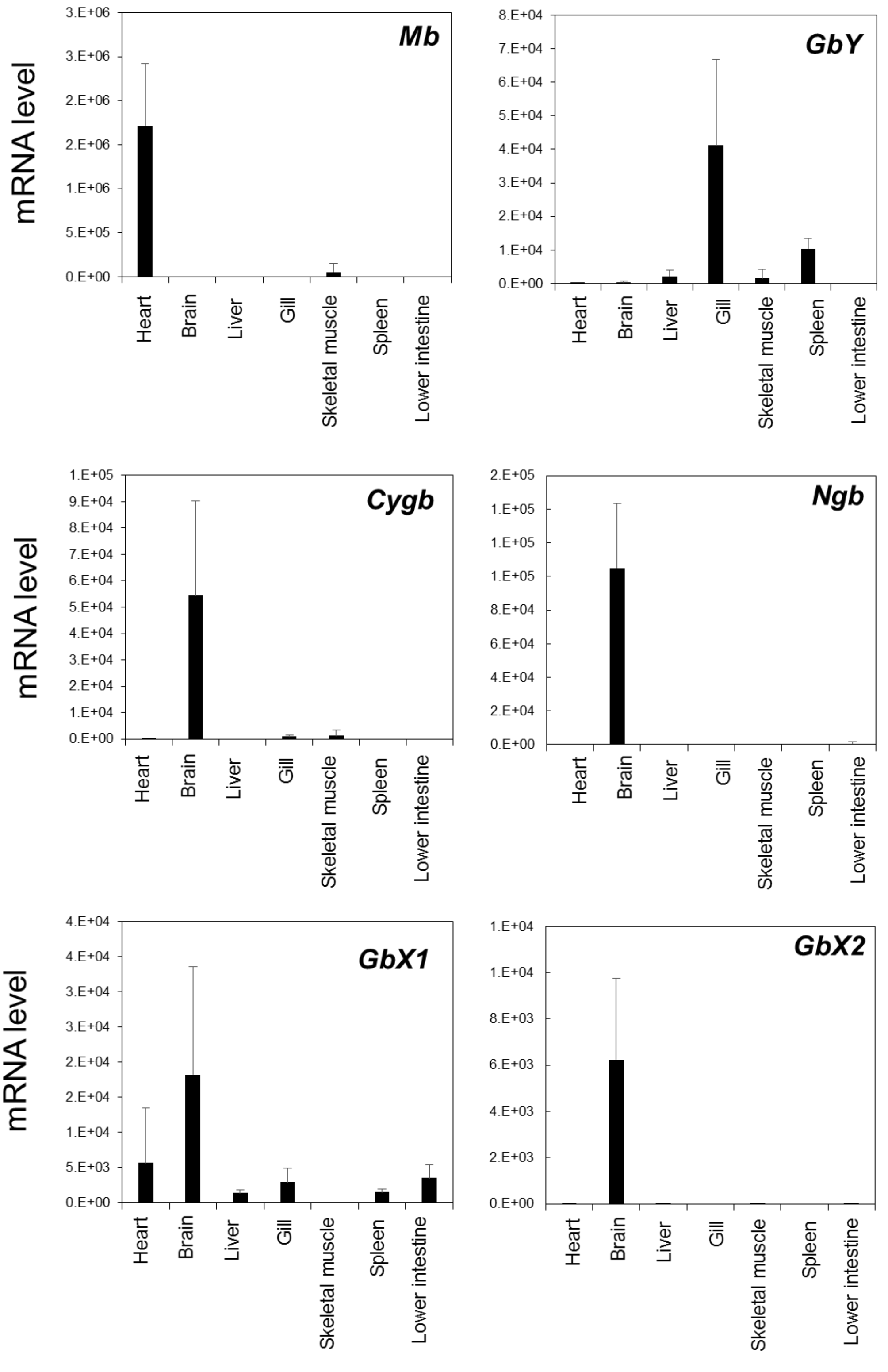

Fig. S2. Bar charts showing a breakdown of globin gene family member mRNA expression levels (relative expression level quantitatively comparable across tissues and genes) summarised for spotted gar in Fig. 6. Data shown is mean +SD.

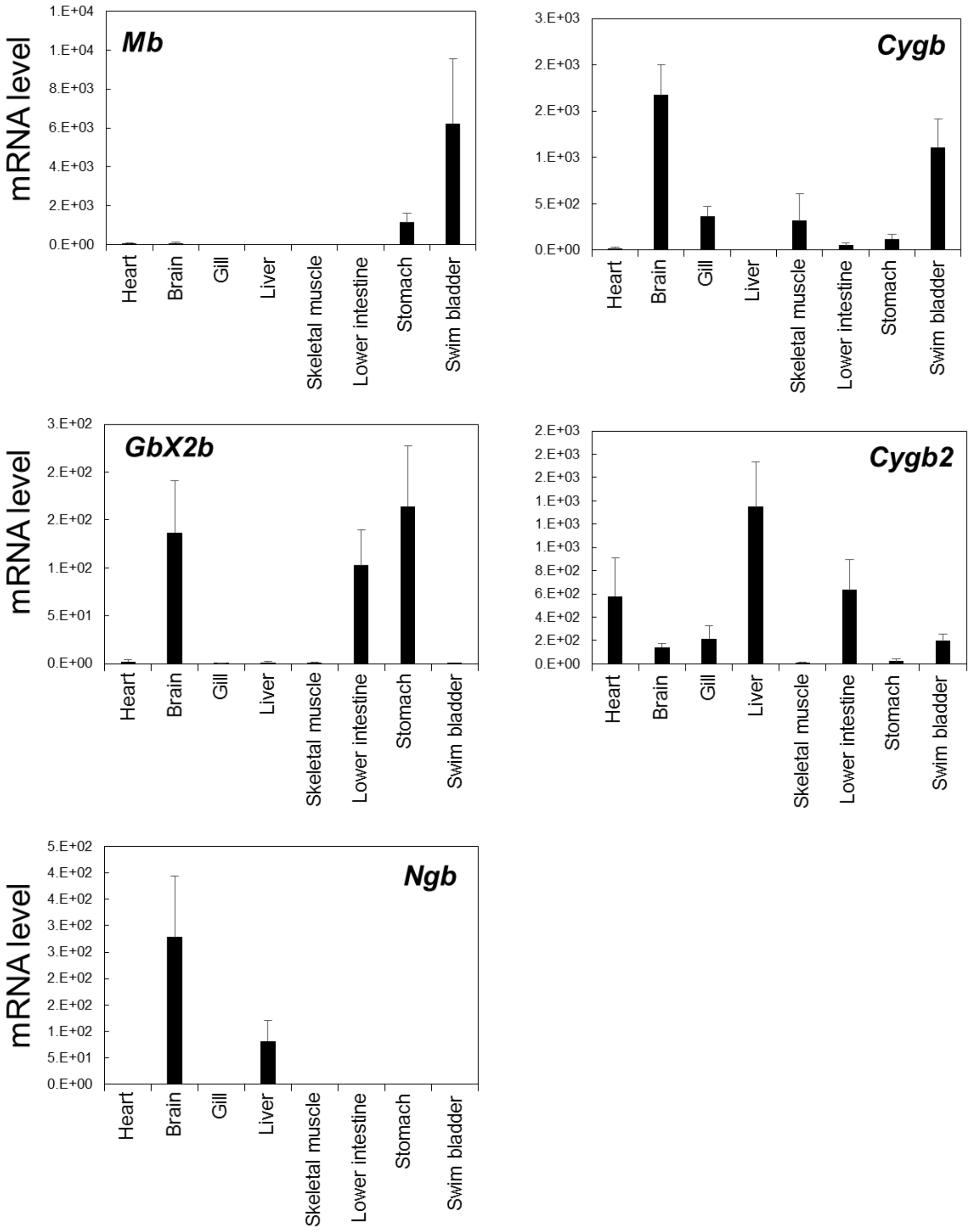

Fig. S3. Bar charts showing a breakdown of globin gene family member mRNA expression levels (relative expression level quantitatively comparable across tissues and genes) for African butterflyfish in Fig. 6. Data shown is mean +SD.

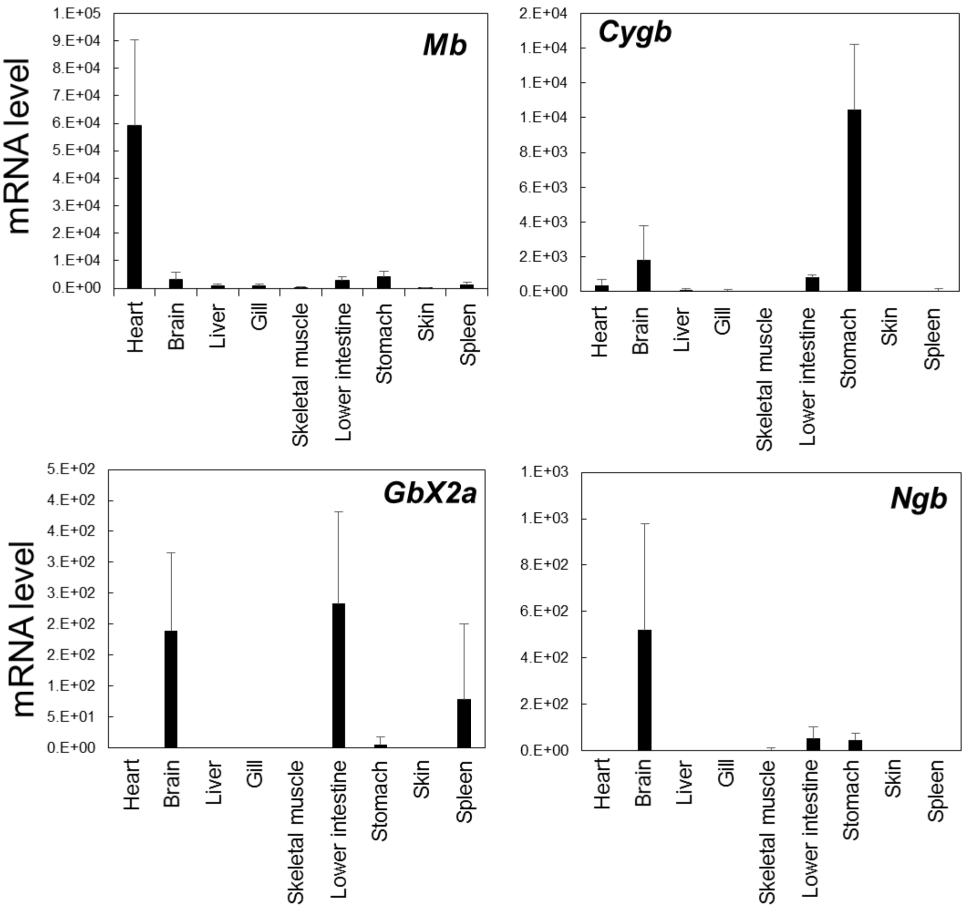

Fig. S4. Bar charts showing a breakdown of globin gene family member mRNA expression levels (relative expression level quantitatively comparable across tissues and genes) for Peters' Elephantnose fish in Fig. 6. Data shown is mean +SD.

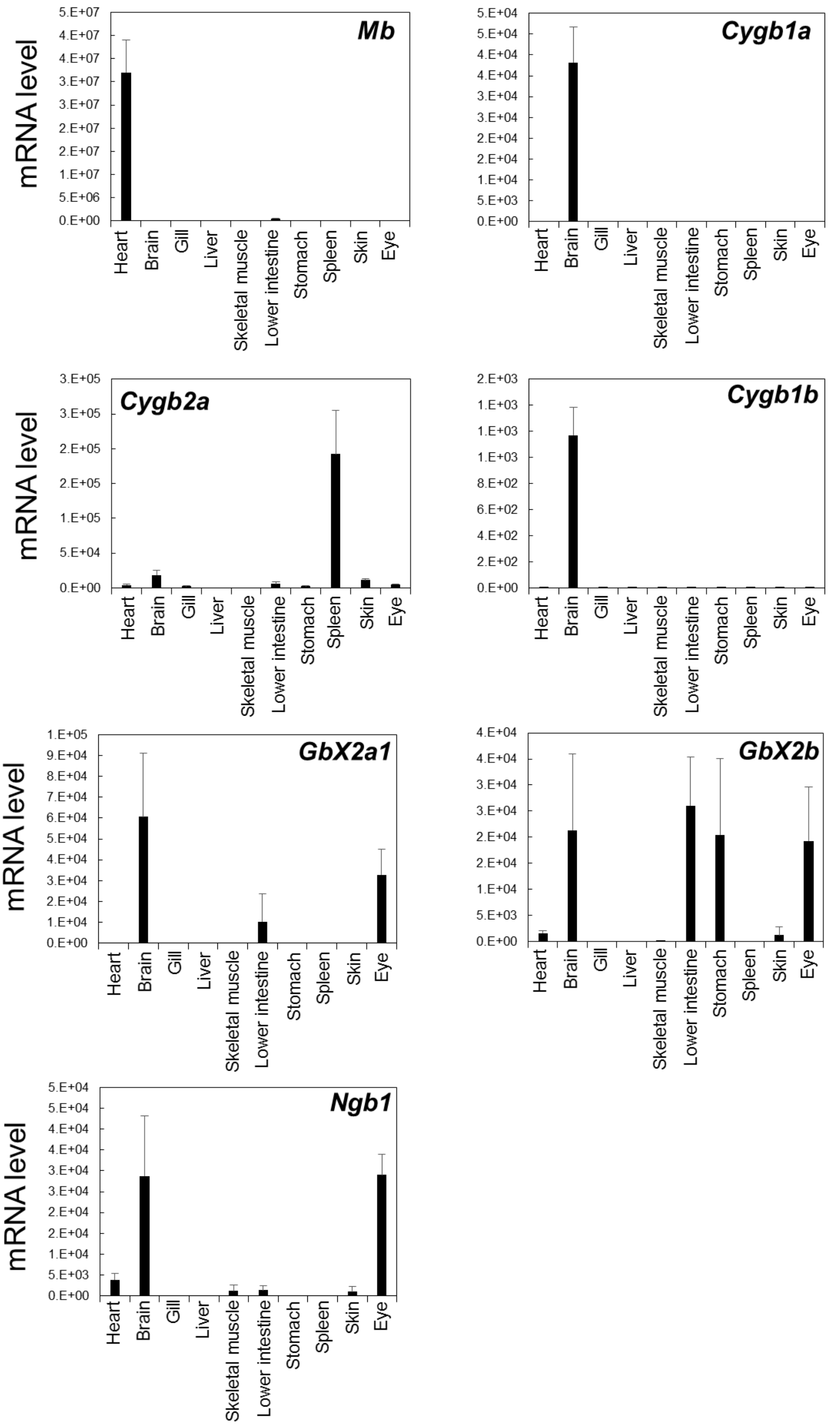

Fig. S5. Bar charts showing a breakdown of globin gene family member mRNA expression levels (relative expression level quantitatively comparable across tissues and genes) for Atlantic salmon in Fig. 6. Data shown is mean +SD.

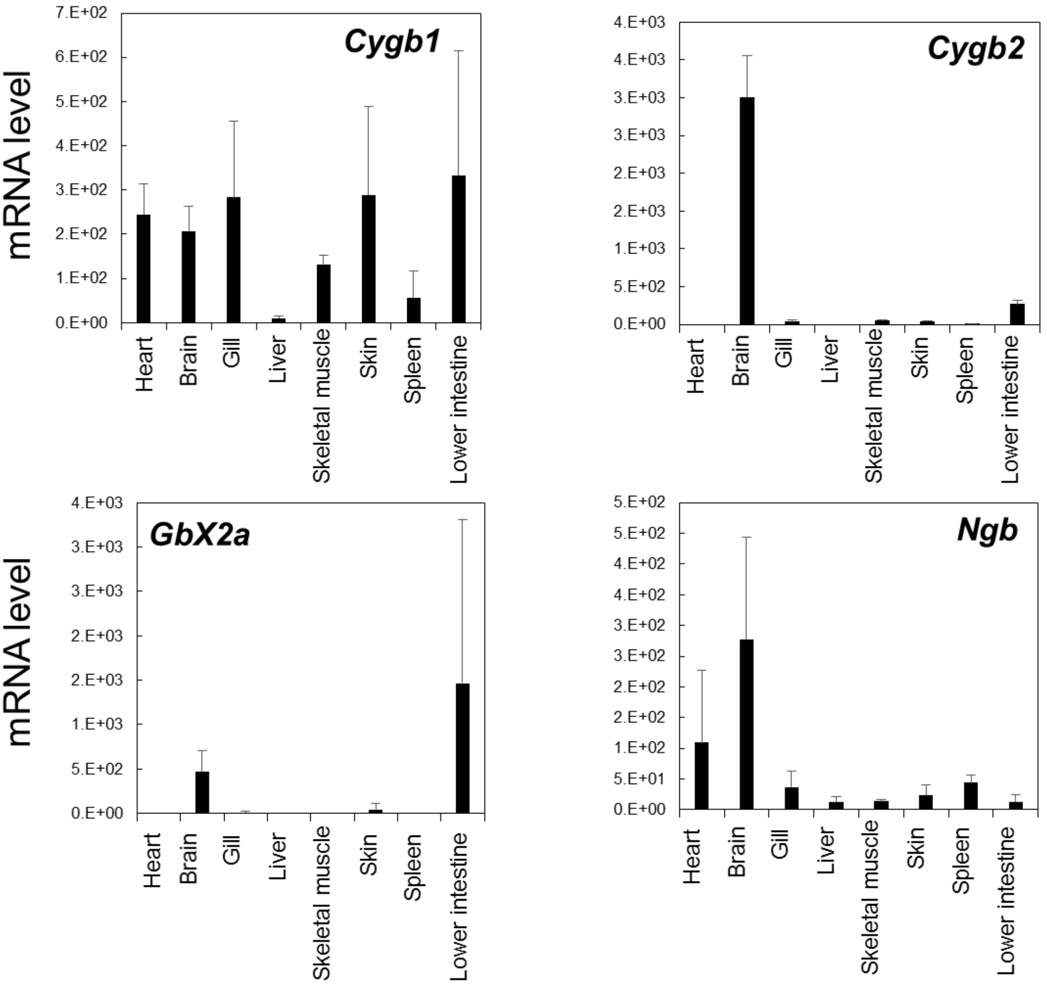

Fig. S6. Bar charts showing a breakdown of globin gene family member mRNA expression levels (relative expression level quantitatively comparable across tissues and genes) for three-spined stickleback in Fig. 6. Data shown is mean +SD.

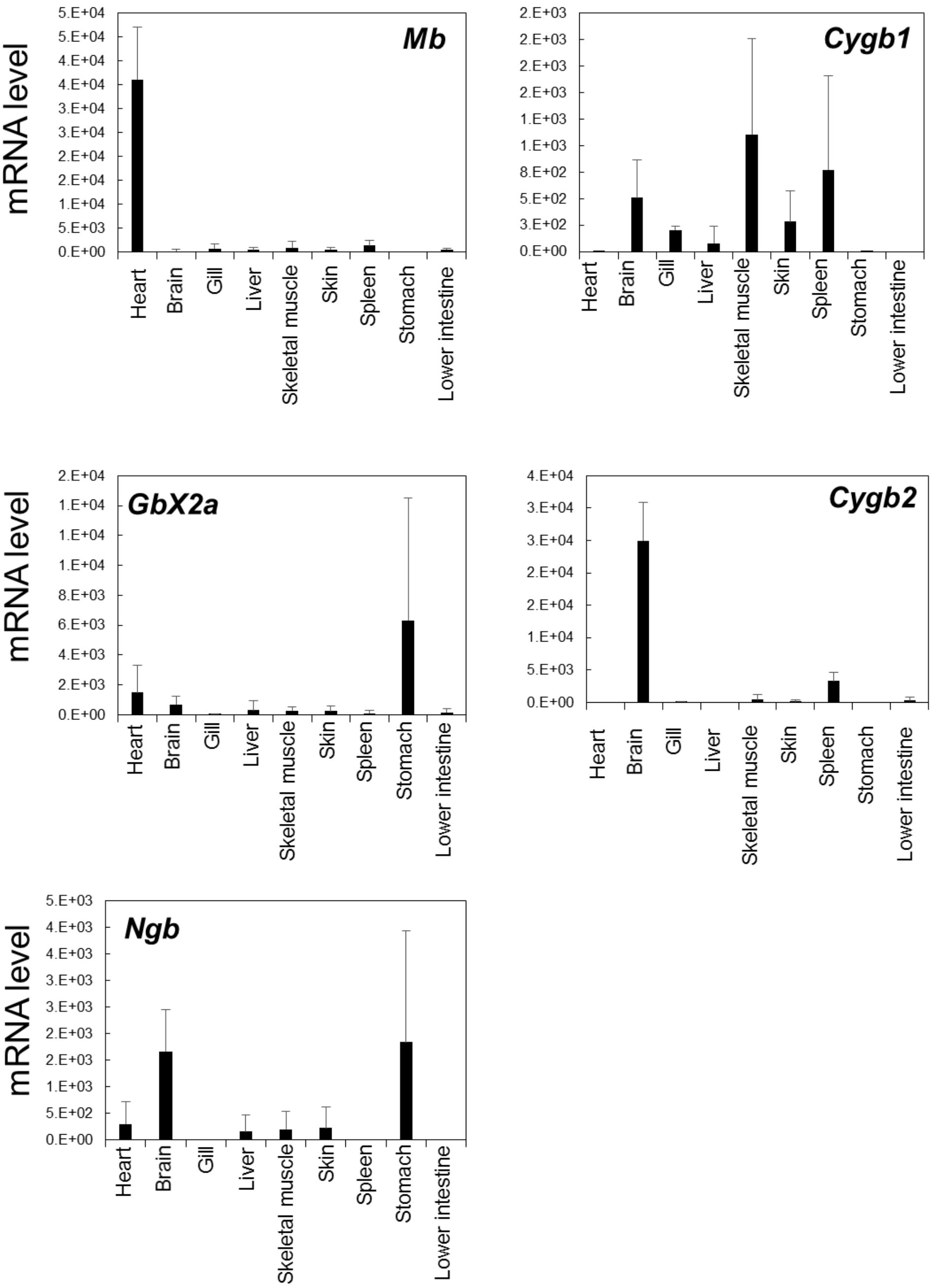

Fig. S7. Bar charts showing a breakdown of globin gene family member mRNA expression levels (relative expression level quantitatively comparable across tissues and genes) for European ruffe in Fig. 6. Data shown is mean +SD.

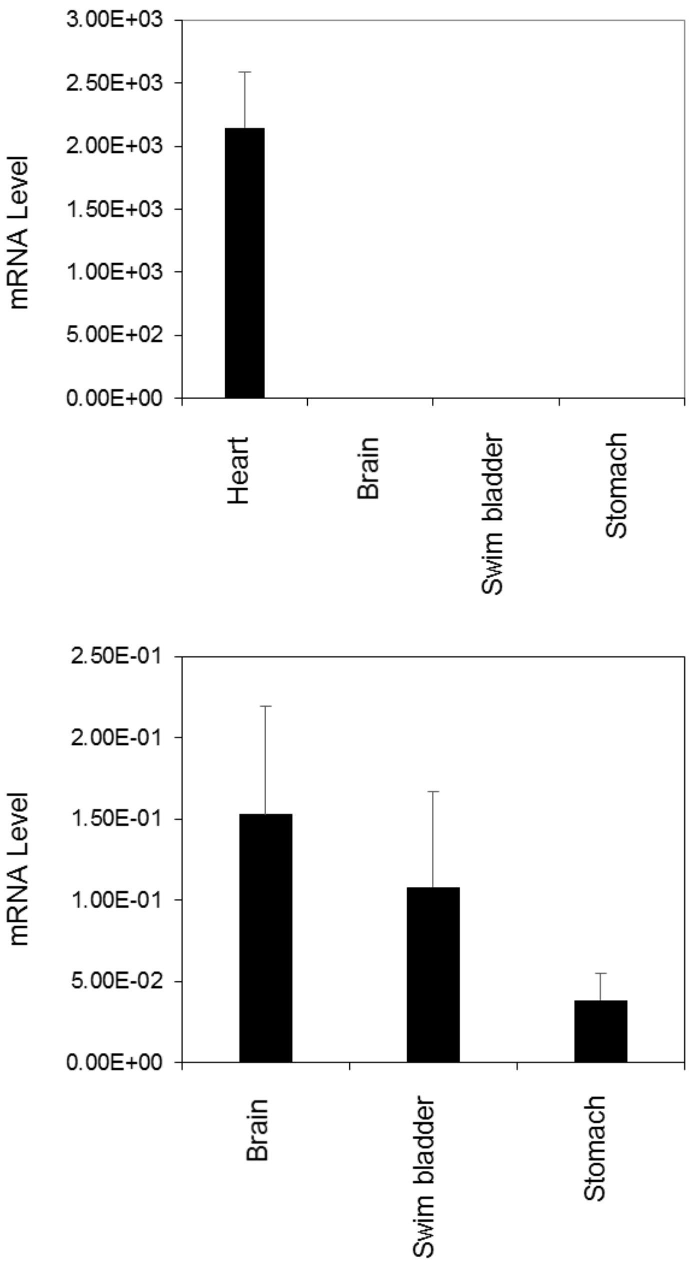

Fig. S8. Relative *Mb* mRNA expression level in tissues from a gill-breathing teleost, rainbow trout *Oncorhynchus mykiss*, providing evidence of low *Mb* expression in the swim-bladder. The same data is shown in both plots, except that heart is not shown in the lower panel.

Table S2. Details of fish species and individuals sampled in the study

Family	Species	Common name	Source	Sample date	SL (cm)	Weight (g)
Mormyridae	*Gnathonemus petersi*	Peters' Elephantnose Fish	Discountfish.co.uk	13/08/2014	13.5	9.9
"	"	"	"	"	13.1	7.0
"	"	"	"	"	14.7	10.7
"	"	"	"	"	10.0	3.1
"	"	"	"	"	13.7	10.4
"	"	"	"	"	12.3	6.0
Percidae	*Gymnocephalus cernua*	European Ruffe	Carpco.co.uk	28/08/2014	8.2	5.6
"	"	"	"	"	9.5	10.5
"	"	"	"	"	10.0	10.8
"	"	"	"	"	8.5	6.4
Gasterosteidae	*Gasterosteus aculeatus*	Three-spined stickleback	Discountfish.co.uk	27/08/2014	3.7	0.4
"	"	"	"	"	4.0	0.5
"	"	"	"	"	3.4	0.3
"	"	"	"	"	3.0	0.2
"	"	"	"	"	4.0	0.5
"	"	"	"	"	3.5	0.4
"	"	"	"	"	3.8	0.4
"	"	"	"	"	3.8	0.4
"	"	"	"	"	3.5	0.3
"	"	"	"	"	3.5	0.3
"	"	"	"	"	3.4	0.3
"	"	"	"	"	3.5	0.4
"	"	"	"	"	3.5	0.4
"	"	"	"	"	4.0	0.6
"	"	"	"	"	3.2	0.3
"	"	"	"	"	3.3	0.3
"	"	"	"	"	4.0	0.6
"	"	"	"	"	3.1	0.2
"	"	"	"	"	3.1	0.3
"	"	"	"	"	3.3	0.3
"	"	"	"	"	3.2	0.3
"	"	"	"	"	3.3	0.3
"	"	"	"	"	3.2	0.3
"	"	"	"	"	3.0	0.2
Salmonidae	*Oncorhynchus mykiss*	Rainbow trout	Gift from Prof. Chris Secombes	29/07/2015	33.0	506.0
"	"	"	"	"	30.5	434.8
"	"	"	"	"	26.5	234.5
"	"	"	"	"	31.5	561.0
"	"	"	"	"	33.0	667.0

Table S3. Primers used in qPCR experiments.

**Species**	**Gene**	**Accession / Source**	**Sense Primer 5'-3'**	**Melting Temp (ºC)**	**Antisense Primer 5'-3'**	**Melting Temp (ºC)**	**Product Size**
Spotted gar	*Mb*	ENSLOCG00000007868	GGAGCTGAGGTTCTGACCCG	62	CAGCATCTCCCCCAGCTTCTTCACC	71	152bp
"	*Ngb*	ENSLOCG00000011029	CATTAATAAGGTGATGCTGGTGG	59	CCCAACGGCAGCGAATGACTG	68	147bp
"	*GbX1*	ENSLOCG00000014709	TCCCTTGACAGAGTCCCAGAAG	61	GTTCCAAGGCAATCTGCTCCA	62	274bp
"	*GbX2*	ENSLOCG00000012798	TAATGTTTGTCAGGTTGTTTGA	54	ATCACTCTGAAGTTGAGTCCATGAGC	63	123bp
"	*GbY*	ENSLOCG00000007868	GTCGTGATAAGGCTGTTCACTGA	60	CGTCAAACAGCAGCTTGAAGTTG	63	255bp
"	*Cygb*	ENSLOCG00000017762	CCCTTCCTCTCTCCGTACCCCA	66	TCACACGGCGGAACTGGACAGCT	71	189bp
Acanthopterygii	*Ngb*	ENSGACG00000007827	GTCATGTTTTCCAGGCTGTTTG	60	CACCCACCAGAGCAAAAGACTG	62	253bp
"	*Cygb-1*	ENSGACG00000012736	TGATGATCCAGGMCTCSTGGG	63	CTGCTTGGABGAGGGGAARYT	63	100bp
"	*Cygb-2*	ENSGACG00000019283	GGGKCASGTCTACAARAACTG	60	SSAGACCTGGAGCCAGCCCAC	68	431bp
Three-spined stickleback	*Mb*	Macqueen et al. 2014	GAGGTCGTCGGTAACGTCTTGG	63	GCGGCACCCATGTCGGCG	71	103bp
"	*GbX*	ENSGACG00000006350	TTGCCAAAGTTGGGATTATTATGTT	62	ACCTCTCCTTCAGGATGGGCTGCAC	71	298bp
Eurasian ruffe	*Mb*	HF546556	GAGGCAGACTACAACGGCAATGG	66	GCACCATGGGCAGAAACAGCTGC	71	146bp
"	*GbX*	HF546552	TTCCGAGAGGTGGAGGACCTGGA	69	CCCGTAGACCGTGTGCCCTGAGCT	72	67bp
Atlantic salmon	*Mb*	AGKD04001111.1	GCCAGTGGAGGCTGACTACAAC	61	GATGCCTGCGAACTTAGGGAAC	63	102bp
"	*Ngb1*	AGKD04000099.1	CACTACAACACAAACTGTGGCA	57	TGTGTACCCCTGACCCAGACTG	62	245bp
"	*Ngb2*	AGKD04000102.1	TTCACTACAACACAAACTGTAGCC	57	AGTGTACCCATGACCCAGACTA	56	157bp
"	*GbX2a1*	AGKD04000154.1	GGAATTATTATGTTCGTCAGGTTA	56	TTGTAATGGTAATGGCTCATCCCT	62	224bp
"	*GbX2a2*	AGKD04000190.1	CGAGTGATGTCCTTTATTGAGAAG	58	CCACAGCCAGAACCTCCAG	58	72bp
"	*GbX2b*	AGKD04000341.1	TAATCAGATGGAACGACTGGACC	61	GATTGGCTGGACGGCTCG	62	216bp
"	*Cygb1A*	AGKD04000540.1	CACTCATTATGACTTTAAGCATGAGG	60	GGATTAGCTCAGATCAGGGGGAG	63	232bp
"	*Cygb1B*	AGKD04000381.1	CTCATTATGACTTTAAGCATAAGA	59	TTAGCTCAGATCAGGGGAAAGCC	64	227bp
"	*Cygb2A*	AGKD04000065.1	TCACAAACACAGAAGGTCAGAGA	57	CTCTCTTCCTCCGTTTCTTTGTT	59	76bp
"	*Cygb2B*	AGKD04000051.1	GAGAGCGAAGGGAGAGAGTGGT	62	CGGGGGAGGTAGGGAGCA	63	155bp
Peters' elephantnose	*Mb*	Unpublished	CCGCCGTTGAGAAGGACTAC	60	AGCTTCTGCGTGTCGGGATAC	62	81bp
"	*Ngb*	Unpublished	TGATGCTGGTGATTGATGCG	61	CGGCTTGATGCTTCCTTCCC	64	92bp
"	*GbX*	Unpublished	CCGCTACAACGCTCCTCCG	64	TCTGGGGTCCACTTCTCCTTC	62	93bp
"	*Cygb*	Unpublished	CTACGAGAACTGTGAGGATGTGGG	63	CTTGTGTTTTATGGCGTGTGC	59	232bp
African butterflyfish	*Mb*	Unpublished	ACCGAGGTTCTAATCCGTTTG	59	TGTTCAGGGGAATCTTGTGTATG	59	228bp
"	*Ngb*	Unpublished	TCTCTCCAGCCCAGAGTTCTTAG	60	CACGATGCTGTACATGTTGAGCC	63	255bp
"	*GbX*	Unpublished	GTCATCCAGGAGGACATCGCC	64	TCTCCAACTGATCCAGTCTGGC	61	204bp
"	*Cygb*	Unpublished	GAACTTCCCTTCAGCCAAGCAG	63	CTGAGGATCTTGAAGTACATGGGC	62	230bp
"	*Cygb2*	Unpublished	GAGACCCAGGAGCCAGCGG	65	CGCACAGCATCTGGAAATAGTG	61	192bp
Targets any teleost fish	*RPS13*	Macqueen et al. 2014	GGAYAAGGAYGCYAAGTTCCG	>56	GGAYTCGTACTTCCAGTTGGG	>56	ca. 105bp
Targets any teleost fish	*RPS29*	Macqueen et al. 2014	CAYCAGCAGCTCTAYTGGAGTCA	>62	CAGCTTVACRAAGCCRATGTC	>58	ca. 160bp
Targets any teleost fish	*RPL8*	This Study	TYGACAARCCCATCCTGAAGG	>62	GTTWCCACCACCGAAGGGATG	>62	ca. 122bp
Targets any teleost fish	*ACTB*	Bower et al. 2008	TGACCCAGATCATGTTTGAGACC	61	CTCGTAGATGGGTACTGTGTGGG	61	ca. 146bp

Note 1: 'Unpublished' primer accession numbers from transcriptome data donated by Prof Peter WH Holland FRS (Department of Zoology, University of Oxford). See Dataset S2 below for more details.

Note 2: The primers listed as 'Acanthopterygii' were designed to be conserved across a full range of Acanthopterygii lineages.

Note 3: Primers designed in previous studies - Macqueen et al. (2014) or Bower et al. (2008)

Table S4. Results of d_N/d_S based branch-site test across the osteoglossiform phylogeny

**Branch**	**Mean d_N/d_S**	**d_N/d_S NEG.**	**Pr. NEG.**	**d_N/d_S NEU.**	**Pr. NEU.**	**d_N/d_S POS.**	**Pr. POS.**	**LRT**	***p*-value**	**Corrected *p*-value**
Campylomormyrus elephant fish *Mb*	0.5609	0.0000	0.9579	0.0000	0.0197	126.58	0.0224	11.453	0.0004	0.0068
Ancestral *Mb-β/γ*	0.5230	0.0325	0.8071	0.0369	0.0058	7.0841	0.1871	10.460	0.0006	0.0110
Ancestral *Mb-γ*	0.6490	0.2328	0.9504	0.0345	0.0000	103.24	0.0496	8.7495	0.0015	0.0263
Silver arowana *Mb-β*	7.3510	0.8940	0.0000	0.875	0.0000	7.7310	1.0000	6.8670	0.0040	0.0700
Asian arowana *Mb-γ*	0.4540	0.0000	0.8630	0.000	0.0030	4.7110	0.134	3.1240	0.0390	0.5790

Note 1: Corrected *p*-value = 1.0 for all other branches in the tree.

Note 2: 'Mean d_N/d_S' is the alignment-wide d_N/d_S estimated under MG94 x REV, allowing variation among lineages (but not codons)

Note 3: 'd_N/d_S NEG.': estimated rate class where $d_N/d_S \leq 1$ (purifying selection); 'Pr. NEG.': estimated proportion of sites fitting class.

Note 4: 'd_N/d_S NEU.': estimated rate class where $d_N/d_S \leq d_N/d_S \leq 1$ (neutral evolution); Pr. NEU.': estimated proportion of sites fitting class.

Note 5: 'd_N/d_S POS.': estimated rate class where d_N/d_S NEU < d_N/d_S and otherwise unconstrained (allowing positive selection); Pr. POS.': estimated proportion of sites fitting class.

Note 6: 'LRT': likelihood ratio test statistic for hypothesis 'd_N/d_S POS.' = 1 (null) vs. 'd_N/d_S POS.' = unrestricted (alternative).

Note 6: 'Corrected *p*-value' after application of Holm-Bonferroni correction.

Dataset S1. Transcript sequence data obtained from Prof. Peter WH Holland FRS (Department of Zoology, University of Oxford) used in the study for primer design (sense strand). Protein-coding regions of each globin gene family member are underlined.

(a) The full globin gene family repertoire found in the osteoglossiform species *Gnathonemus petersii*

>Mb_Gpe_TR5525|c0_g6_i1

AATGATAACAATCAGAAACAGCTGATAAACACGGGGAATCCACATGGGGTTAACGAGGGGGTGTGGCACATGGGAGGAGC
GGACGAGCGGGGCAGGACAACATGCATTGCAACACTAACTTTTCACCCTACTGTCCCAGTGGTGTTTGGCTCATGGTACGAC
CATGTGAAAGGGTGGTGGGAGAAAAAGCAGACATACGCCAACATCCACTACATGTTCTACGAAGACCTCATTGAGGACACG
GCCCAGGAGATAGAGAAGATCTGCTCCTTCCTGAACATCTCACCAACGCAGGAAGAGAAAGACCATGTTAGAAGAGAGGTG
GGATTCGATGCCATGAAGAAGAATGAAATGACCAACTACTCCACCCTTGGTGTATTTGATTTCAAAATATCACCATTCATGCG
AAAAGGAAAAGTTTCTGACTGGAAAAATCATTTCACTGTGGCTCAGAATGAGCGGTTTGATGAAGTCTATCAGAAAAAGATG
GCAAACACTACCCTCAGGTTCAGGACAGAAATATAGCTTGCAGTGTAAGAATAAAAGCCATACTCAGGCTCATGTACCTGTG
CTGTTCAAAGGGCTTCCACCAGATGGAGCTAAAGATTTCTAGAAGCAGGATCAGCCTGAATGAACATCACAGGCAAGATAC
AAGATACTTTAATTGTCACATACATAGTTATTCAAGTACATCATGTAGTGAAATGTACCCTGAACGACCCTTAGACTGTGCAA
ATCAAATCAAAAATACAAGTAAGAATAAGTCAACAGTTAAGCCAACAGTCATCCGGAAGGCAATCCTCTATTGACACATGTT
CAACTTGAGGGCTCTAAGAACAGGCCATGGTTTGAAATCACACATATGCTTGTTAAACACCAAGCCTTCGTTATCTGTAATTT
CTGGATGAGGTCAGCAGGGGGCGTCAGAGCACCGCCTAGAATTTCAGCGCGCGCTCTCAGATGTGTTTCACCATAATGGCCA
CCAGGGGGAAGCTCATTGTATTAAAACAAACTCCATTCTCAATTACATGTGGAATGGCCTGGGAGCGTGCCCCTGTACATGA
CTGAGTGAGAGTATGTGGTTTCCTCCAGGCACGGGGGCTTTTTAACTGTTCACATATGCCCCAGAATATATACTTCGCAGAAA
TAGTACTAAATTATTTCCCATTTAAATGATTCGAGTGAGACTGCGAAATCCACATCCATATTGAGCATCTGCAATGGGGCTCC
CTGGAATAATTGTATCTGCACTTAGATACCAACTCCAAGTGGCATACTTTGGCTGTATCATTAAGTTCCCATCAGCAAAGTTA
TGATTAACGAGATTGCTGAGAGTCTGTTTTTCTCGATAGTGCCAAAGACTATCTTTGGTCAGTGTCCTTTCCCCTTTGCATAGG
CCGAAAGACTGTCTGAGATCCTCTGTCTAGATGCCGTCGAACTGCTGTTATCTTTCTATCTGTTTATCCCTATCGTCTCCCCAC
ACCCTTCCCTTCCTTACATTGTCGCGGTGGAAAGGCAACGCATCTGATATCTTTGTTTAGCCTCCCCTGGGACGAGGGACCCT
TTTCCCCTAAGTTATATACAGCCCATGATCAGTCATGTGATGCAAAAATCCACCGTCAATGTTTAACCGTTAATGGCAATGAT
TTACTTTGCAGATAAATGATTTGCTGCAAGCGGTATTGGATGAAAAAGTATCATTTTTTGAGGGAGATGAGGCTGGTCCATCC
TTTGTTTTAGTGACGACATTCTTGTCCCCTCAATGAACACGATGCTCAGTGTAGGAGTTGGTGTCCATATCAAAAGAGCAGAG
TCAGAGAGAGAGGAATTCTACGCCACTTAATAAACCCAAGATACTTCAAAACATGAATTATGCACCATACAGTTTTCCGTTG
TAGTAAAAATGTTTTGCAGTGTTTCTGTAAAAAAAGAGCAGAAAAGTGTTTTTCTTTTCTTTTTTTAAATTACACTTTGATCTT
CATTGTTCTGATACATGCTTATCCCCTGAAGCCGGATTTTCCACTTTGTTTGATGGCTTTGCCAAAGATGCGGATTAAAAGCC
AGACTGCTTGGGCTCTGCTGACGCCCATATATTTTATTTAACATTTGGAGCTTGAATTTTGAACCTAAGTCGAGAGGGAAATT
GTATGGAACTAAACTTATCTGTGACCGAATGCATTAGGAAGACTGGAACAAAGTGAACAGCTGCAGAAAAGCCATGAGAGA
GCGAGGCGAAGTGAACAACAGACAGAAGAGAGTGAGGCCAAAAGGAGAGGCCAGAGAATGATACAGATAGAGAGGTAAA
TTCCTTTTATAGAGTTTCACCTCTCTTGTGGAAACCTGAAGAGGCTTCCATTGAAAGCTGTCCCCTTTTTCTCCTCAAAACGAG
GACATCACGATCTTCCCTCTATCTATGACCATTTTCGTCATTTTTTGTCATTTGTATGCTTTGGCAACATTCCCTCCTGGTCACG
CTAGCAGTCTGGCTCGCCTTGGACCGATAACCTCTAATCACTCCCCAATGTCACTGGGCCACACATGAGCCACGACCTGCCAT
TTGTCGAATGTGTCAGCAGTCACACATGGAAAGTTCACCGTTTTGTACGTGGGGTAGCTTGTGACCAAGAACATGAGGCAGG
TGAAGTGATGAATTATTTAGTCCGAGACAAAATGTTAGGGCCGCAAACCTGTTTCGAAGCAAAACGTACAGTTTGGAATCTG
AAAACAAGAGTCAATGTACTTAAGTTGAGTGCAACTAGTCCTGGTAGTTTAAATGAAGAACCAAATAAAAAAATAAGATGA
ACTAAAAGTATGAGACATCCATTAAAAAAAAAAAAAGTGTGGAACAATGCTGTAATAGTATCAGGGAGGGAGGAAAAGAGG
TGGAGTTAATTCTTAAGACAGGAAAAGGGGGGAGAGAGAGAGATTGATGGTTGGGCTGTAATTCATGAGTGAAGTTGAGGA
GTATAAAAGGTAAGGTTTGGGACTTGGAACAGTACACTGAGGCATTAAACAATTCACATACAACTTAACTCGGGACGTCACC
TGATTATCTTGTACGATGTCTGACTTTGACCAGATTCTGAAGTACTGGCCCGCCGTTGAGAAGGACTACAGAGGATTTGGCGG
CGAGGTTCTGAACCGTCTCTTCAAGGAGTATCCCGACACGCAGAAGCTGTTCCCCAAGTTCGCTGGGCTTTCGCAGAGCGAG
CTGGCCGGCAACCCAGGAGTGGCGGCCCACGGCGAGACCGTGCTGAAGAAGCTAGCGGAGCTGTTGAATGCCAGGGGGAGC
CACGGCCCCATCCTCAAGCCGCTGGCAACCAACCATGCCAAGACACACAAAATCGCTCTCAACAACTTCAAGCTGATCACCG
AGGTCCTTGCGAAGGTGATGGCTGAGAAAGCCGGGATGGACGCGGCTGGACAAACGGCCCTGCGGAACGTGATGGGAGTGG
TCATCGCAGATATAGACTCTGTCTACAAGGAAGTCGGTTTCCAGGGTTAAAGAAAAAACCCCCACCATAAACAGAATTGCAG
ACTTTGTGAAAATTAATAGCAACTTACAATAGCGCTTAATCGTCTAATAGACATAAATGATAAATAACTCAGGCGCCACACT
ATATGGTAACTGTACTGCAAGGCTATTAACTAAGAGCTGGTAAGAACATGTTTAATGCTAGTAGCTATGATTCTTGGTGAAA
GACTCAGAGGGGAGAGAGAGGGTCTGACCGGAGACATGACTTGCTTTCACATTTCTAAAGGGGCCTTATATAGACAACAATT
TATTATTTTCACTATTTCTTCTAACGCAATCTTTCCTCTTGATTCTAGCTATGTAAGCTTGATTGCTGTTTTCATAAAGAATCAA
CTTGTTTTTGCTGTGTTCTGAGGATCACAATTAAAGGGAAATTATATACATCAAAAAAAAAAAAAAAAAAAAAA

>Cygb_Gpe_TR17354|c0_g1_i1

CCCGTCAGTAATGTGTCGAGGGAGAGAGAGAGGGGGTAGGTAGTGGGTTGGTTTGTTATACAGGAATACTCACTGCTATCTC
TGTCAGGTCCTCAAAACCATAAGACACAAGTTCAGAGAAGAAGAGCTCATTCTGAGCCCAAGAGTGGAATAAAGGACAGAC
AGAGGACAGAGTCGGAGGAGAAGGGTGCCTAGGGGTGAGTGTGGCTCACCCTCCTGATCAGCTGTCACCTCCGTCCCACGTG
GGTTCTGCTTTCTTAATTCCCCTCACAGCCGCTGTTGCTGCTGCTGCCGCTGGCAAGCTCTGTCTTGGATGGAGGAGGTGCGA
GGAGACGGGGCTATGGAGCAGAGGGAGCGTGCCGACCAGCTGTCTGAGGCGGAGACGGGCATGATCCAGGACACATGGGG
ACGCGTCTACGAGAACTGTGAGGATGTGGGAGTCGCTATCCTCATCAGGTTTTTTGTGAACTTCCCCTCGGCCAAGCAGTACT
TCAGCCAGTTCCAGGACATGGAAGACATGGAGGAGATGGAGCGGAGCACTCAGCTCCGGAAGCATGCCCAGCGCGTCATGA
ATGCCCTCAACACCGTGGTCGAGAACCTCCACGATCCAGACAAGGTGGCCTCCATCCTCAACCTGGTGGGCAAGGCACACGC
CATAAAACACAAGGTGGAGCCCATGTACTTCAAGATCCTCAGCGGCGTGATTCTGGAGATTCTGTCTGAGGACTGTCCCGAA
TCCTTCACCCCGGACGTGCAGAGGGCCTGGACCAAGCTGATGGGCCTGGTGTACTGGCATGTGACTGGGGCCTACACCGAGG
CCGGCTGGGTGCAGCTCTCCAGCTCTGCCGTGTGACTGGGGAACCGGGTCCTGCTGCACCCCCATGTGGCCATCCTTGACCAA

TGCTACTGATTTGTTTTAAAGCCTGCGGGCGTCTTTCTGGTGCCCGTCCGCAAAGGGACTGGACTGGGTGGGAGTGTGTAGGA
CTTAGGCAGGCAGAGAGGAGAGTCTGGAGATTGTCTGGAAATAGTATATAAAAAAAATGCCAACCGCTTTCGAGGGCCTTTT
CCTGCTCTGTGCCGAGGCACCTTCACTTGTAAATGTAACTGCCAAGTTGTTGTCCGTGTCGTTTCAGATCTGTGAGTGTGTTAA
TAGGATTGCCCTCCTTAGAGATTTACTGGTATCCACCTGGACCCTCGATTCTATGCCCCGGGGGGTGTGGATTCCCTCCCCCC
TCATGCCTCTCCTGCTCCGTCAGTCTGCACGCGCTTGTGATTTCCTTTCGCGCTCCAGAAATATATGCACACATACACACACC
ACAGAAAAATGGCATATGGGATAATACAATATGGCGCGTTACCGTGACGATGCACCACTTCACTGCACCAGTGAGGAGTGA
GGACACTCTGAAGCATGTGCACGGCTCAGTCCACGTAGACGACACACGCAGCAAAGCAAAGATCCATCAGCTGAAGGTCAC
TGGAAACATATCAGACCTTCTCCACACATATGCATTCGTTTGTACTTTCATAAGTAAACACATTCTGGAGTCTTTAGAAGGCA
CTAGTTTAAGGCACTCCGTTTGTTTCATAACCTTTGGTGTTGAAATTTTTTTTTTACATCTATTGAAACAGAACTAAGAGAATA
ATTCAAATTTGTTCCTGCTGTGGTTTCCCCAAACAAGCTGTCAGACTAAACAGAGCGGACTGCAGAGGCCAAATAGAGCCAG
TCTCTGCTGACCCCGAGCCTATTCCTTTGATACCGCGAGCAGGATGCTCTCGTTTTATCAGGAGCTGCATCCCTGCTCTCCTCT
GTATTGTCATTTAGTACTAACGAGGACTTCATGAGTCCGTCCGGCCACCAGCATCTCCCTGCTGGCTGTGCATGAGATACAGA
TGCTTGGCCATTTGTTTGTGTCTCCCCGTTCTGCTCTGATATTACTGGGAGTGGTGAGGGACCAGGGGGGGATTTTAAACGTA
ATTTGTGCACCTCTTCCTCCTCCACAAAGTAAGAAAAAAAAATCCCCTGAAGCTCAAGGACAGCAATTTATTTTTTATTCCGCT
TTATTTTTATCGGCACCTGTGCGCAAATTGCACACGATTGCCGTACAGACACGGTGAAGTTAACCTGCTTCCCAGTTCCGGGG
AGGAGTCTTTTTCAACTATGATGTTGTTAAATCAAACCCCAGCTGTCATTGGCCAGGCATCAAGTGGACTCCGCCCTCATTCT
ATGGCCCCTCCTATTTTTTCCAATATCCTCTTTATATAACTTTTATAGCAAAGCGATGTTTATACTCCAGCTGTTTCTGACTGTT
TCAATGAATTGTTTATTGCGGTGGGCAGTTAATATTCACAAAATGGCGATGTACTGTTCTTTCTTCAAAGCGCAAATCCTGTC
ACGTGATGCTTCTGGTACTGTGCCCCGCTCACCTGTCGACAGAGACACCCACGCTGCCGTTTACAGATAGAAAGGGTGCATC
CTCCCCTCCACCATGTATTTCGGGAAGCCAGCTTGAGTCCTTCACATCAAAGGGAGCGGAGGATGAGGGACAGAAAACCGGC
GGGTCAGCATTGCCACCTGTCACCTACCATCCACCCGCTCGTGAACACTGGGATATCCTGTTTGCCGGGAATCTTGCTCCCTT
ATGAGTGTCTCTAGAGTGGGGGGTAACCTTACACTGGTGGGGGGTGGGGGGGATGGACGCGTGATGATTTGCAGAATATGTT
CATAACGTGAAAAACATGAGAAATAAGGAATATTCAGTGTTAACTATTGGTCTGTGGTTCCCCCTCCTACTGGCAAAATCTA
CTGAGGGACCTTCTCCCCCCCCCACAAAATGAGGTCATGGGCTGAAAAGTCTGGGAAGCCATGCAGTCACATGTCTTCAAGC
TGGGGTAACACAAATTACACACAAATTACACACTTGCTAGCTGTTATCATTCAACATCTGTTGGCAGGAAATGTTCTCTAGGC
GTGTTTGTTTTTACCCAGATGATCATTGTGAATTGTTTATTTTAAAGTCTGGACTTTATATATTTTTTAAATATACAAAAAATA
CAGATTATCAATATGTGCTATATATAATAAGATTATACTCTGATTCTGATATATATATATATATATAAATCTATGGTATATAC
AAATTATTGTACATTGCTGGTGGAGGACAATGTCAATTTTGTGCTGTTATAGCATGTTTTCATGATCTAAGACAGACTTATAC
CTTAGCAAACCTGTCACAAATAATCATGACTATCAATAAACATGTCAGGG

>GbX_Gpe_TR216201|c0_g1_i1

CGTGGGAGCAGATCGCAGATCATGGGCTGCGCGATATCGGGACTGGGTCTAACGCCGAAGTCGGGGAGGGGAATGAGGCAC
GCAGAGCCCGCAGCCGCGAGTCTGAGTCCGGAACAGATCGACATGATCCAGGAGTCCTGGAAAGTTATCCAGGAAGACATC
GCCAAAGTCGGGATTATCATGTTCGTCAGGTTGTTTGAGACGCATCCTGAATGCAAAGACGTCTTCTTCCTGTTCCGAGACGT
GGAGGACCTGGAACGGCTGAGGACTAGCAAGGAGCTGCGAGCTCACGGACTCCGGGTGATGTCTTTCATTGAGAAGTGCGT
GGCCAGACTGAACCAGCTGGATCGCCTGGAGCAGCTTGCCCTGGAACTGGGGAGAACACACCACCGCTACAACGCTCCTCCG
AAGTACTACGGGGTAGGACTCCTACTGCTGACGAGGAGGCTTCATTTAATCATGCAGAATCTGTGGGATGGACCCCATTCAC
AGCAAAGCTCCACAGTACAATAAAAACCCTCTTTTAACATAAGACCACACCACATTACCGTAGCTTATGAAATCGACATTGA
GGGTTTCCCTCCCTCAAGCCAGTCGCCTGGGCTCTTTCAGTACGTGGGGGCGGAGTTTATCTGTGCCGTCCAGCCAATCCTGA
AGGAGAAGTGGACCCCAGAGCTGGAGCATGCATGGCAGAGTCTCTTCCTGTACGTCACTGGGATCATGAAGCGAGGGTACC
AGGAGGAGGAGGAGGAGAATTACAGCACCTCCGTGGGAGCCTCCAAGCGAGAGAGACCG

>Ngb_Gpe_TR20833|c0_g1_i2

GGGGGGGGGGGAGCTGGTAAAGGTAAGGTCAGCCCTGATCACCCGCAGCTCAGCGCAGCAACCTGCAGAGGGAGCTGTGGG
CCTCCGCATCTGCGGAGGAGAGCGAGCGAATGCTCCCGCTTCCCTTCACTGGAACAAAGCGGCAGAGAGTGAGAGGGAGAG
AGACGGCAGCCCAAGTGACACAGGAGCCCCAGGGCTGCCTCCCTGCCTCATTTGTGGCATCGCCGAGTCCGACTGCACAGGT
GCCAGTCTTGCCACTGATGGACCGGGAGTGAGGATCCTCTGAACCAGCATCCAAAGGAGTCCGAGCAGAAGTTGCCCCCCGC
GGGGGTCCGTCTCCGGGGCGTATTATGGGTCGGCGGTTTGCGTGAGAGCCGGTCCGTGTCGATTCCGTAGCATCCAAAGGCC
GACGTCCAGGGGGGGGGGCGGCTCCGCTCAGCACCGACAGCATGGAGAAGTTGACTGGGAAAGATAAGGAGTTGATCCGAG
ACAGCTGGGAAAGTCTCGGGAAGAACAAAGTGCCGCACGGGGTCGTCATGTTCAGCAGGTTATTTGAGTTAGATCCTGGGCT
TCTCAGCCTGTTCAGCTACAACACCGAACATGAGTCTGCACAGGATTGTCTCTCCAGCCCTGAGTTTCTGGAACACGTTACCA
AGGTGATGCTGGTGATTGATGCGGCTGTCAGTAACCTTGACGACCTCCACTCTCTGGAGGACTACCTGCTGAACTTGGGAAG
GAAGCATCAAGCCGTCGGGGTTAAGACACAGTCATTTGCCGTGGTGGGAGAGTCTCTGCTCTACATGCTGCAGCGCAGCCTG
GGTGCGGCCTACACAGCCGCCCTGCAGCAGGCATGGCTGAACATGTACAGCATCGTGGTGGCAGCCATGAGCAGCGGCTGG
TCCCAGAATGGTGAGCGTGGCAGCGACTGAGCCTTCCAAGTCAAGGAATCCACCAATCAACCAGTCAACGTGGAGACACGT
CGTAGTTGTAGCTTTTACCGTTGAAAGCATTTTCATTTTTCTATGCGCCACGGGGTCTGACTGTGGGAGGAGTTGATGGAGAG
ATGAAGTGCGGGAGAACTGTTGGCGAGTGGTTTTCAATGGCAGGGACCCAGCTAGCACATAACGTTCTCGCATAACGTTCTC
GCATAGCGTTCTCACGCTGCCGATACGCCG

(b) The full globin gene family repertoire found in the Osteoglossiform species *Pantodon buchholzi*

>Mb_Ptd_TR106175|c0_g1_i1

TGGGACAAAGTGAAGCTAGAGGTTTGAAAGGTATACACAAGACTTCACCTGGGACAGCATTGTCTGACTGCTTAACGACCAT
GGCCGACCACGATCTGGTTCTGAAATGCTGGGGAGTAATTGAAACAGACTATGCAGGATATGGAACCGAGGTTCTAATCCGT
TTGTTCAAGGAACACCCAGAGACACAGAAGCTCTTGCCGAAGTTTGCCTCGGTCCCACAGAGTGAATTGGCGGGAAATCCGG
CAGTGGCTGCTCATGGCGCTATGGTTCTGAAGAAGCTGGGCGAACTGCTAAAGGCTAAAGGCAACCACTCTGCCATTATAAG

GCCCCTAGCCACCAGCCATGCCAACATACACAAGATTCCCCTGAACAACTTCAGGCTGATCAGTGAAGTTCTTGTGAAGGTG
TTGGCTGAGAAGGCTATACTAGATGGAGCAGGGCAGGATGCTCTCCGGAGAGTGATGGGAGTGGTCATTGGTGATATTGACA
TCGTCTACAAGGAGATTGGTTTCAATGGGTAGATAGTACCCTTTGATTTGAGACTGAAATGCTTTGACATCTCGCTTTCTGAA
ATGGCAGTGTCTCAAGCACTATGCGTTTAATTTCTACAAGAGTAATGACTGCATGCTGCATAGTTCATACTTAGTTCTATTAG
ATACACCTGTATCTAAAGCAATATTTGAACGTAGGAGTCAGTTTATATCATTATCTATTAAATCAACATTTGAGCATGAATTT
TTGGTTATATATCATTCTAAAATATATCATTATTTACAGTGTATCTGATTGATTTTTGGCATTATCATTGTTGATGCAATGAAT
TCTTTGGCTTATGATTTGTGATTAAAGTATTTCAC

>Cygb_Ptd_TR392|c3_g1_i1

GGCCAAGCCGGGGGGGGGTGGGGGGGGGGGGATTGGCGAGAGCGGGGGGGGGGGGGGGAGCAAGTAGAAAGAAAGAAG
GAGAGAGCACATCAAAGGGAAGGAGAGTAGGCTTTCCATTTGGAGCAGTTGCTGGTGCCGTCTCGCCTCGGGCAGGAATCTA
CAGTGGATGCAGAAGGTGCTGGCAGACATGGAGCACAGAGAACGAGCTGACCAGCTGTCTGAGGCAGAGAAGGCGATGGT
GCAAGGCACCTGGGGCCGCGTGTACGAGAACTGCGAAGACGTGGGCGTCTCCATCCTCATCAGGTTCTTTGTGAACTTCCCTT
CAGCCAAGCAGTACTTCAGCCAGTTCCAGGACATGGAGGACGCTGAGGAGATGGAGCAGAGTGTGCAACTCCGCAAGCACG
CACAGCGAGTCATGAACGCCATCAACAGCGTGGTAGAGAACCTGCACGACCCTGACAAGGTTGCGTCCATACTGGGACTGGT
GGGGAAGGCGCACGCCATCAAACACAAAGTGGAGCCCATGTACTTCAAGATCCTCAGTGGCGTGTTGCTGGAGGTTCTAGCT
GAGGACTTCCCTGACTGCTTCACCCCAGATGTGAAGAGGGCCTGGGCCAAGCTCATGGGCCTGGTCTACTGGCATATCACAG
GGGCATACATGGAGGTGGGCTGGGTCGAGCTCGCCAGCTCTGCCGTGTGATCACGGAGTCTGAAGTGGCCCACTTCGACAGG
CCATCTTGGACCAGCCTGACTTGCTATTTACATTGGAGGGAATTAGTGTATCGCTGTAGCTCTTCTACATGGGGCCAATGATA
AAACAGAGAAGTGAGCAGTGAGGTGTAAAAGGTGGGCTGATGTTTGGTTGAGGGTTGAGTTGGAAAGCGATCAATAGATCT
CTCGGAATTTGGGCAACCTGCAATTGCTTATCCCACATTCCCACTGTTGGGATCAATTTCTGTATTCTAAAGTAACATAGCAT
GTATTTTTAACTGTTAAGCTGTTGTTTGTGTTATATATGGCTAGTGGGTTAATGGATGTCTAAGTTCCTAATCAACACTTTGGT
GTGATTAATAAGGATATGGGGACCAATCTCATATTTGTATCTGACACATTTTGTCCGTTATGTTTACTATATATATTCTCTGTG
TCTTTTTGGTAAGCATCATTATTACACTGTCTTGTTAAAGGATTTTGTCACTGTCACTTAATGAAATGTCTGGAAAAGTTTGTG
ATTGAGTATAACCTTGGTACGCATACAAAGTACAGTAGGCTGTCTGTAGATAACTAACAATGCCTCAAATCACAATATAAAC
TATATTTACACTAAGCAACTAGGACACATTGCAAAGAGAATGTGTGGTGTTTTGTAAGCAGCAAAAAATCTCTATGTTTAAT
AGATCCAGGAGGATTATTTGATCAAATATTGAAAAAGAAAACACCAAAAGGATCTTTTAAAAAAAACACTTGCTGCCCAAA
AAAATCAAATCATATTTCTACTGCTTTTTCCACTAAGTAATTCTTTTATCATAAATGTATGTTCCTTGCATTTTCTGCAGAATTT
TAAGGCCACAATCCCTGCCCTCCAAACCTTCCTCTTTTTCATTTAACACTAATGAAGCAGTAATGAGTCCCAGGGGTCATCCC
TCCACATTCATCTTCTCTCCATGTACATGGAACCAGATGCCTGCTTTGCTTGTAATGGTTGCCGAGTGGAGAATTTAATATGG
TTTGCACTTCTCCCTTTTGGTAGTTAAAACCAAGAGGTTGAAGTTTATGTGAGGCTTGCTTGTCAAGCACTGAATGATATAGT
TAGACATGTGCATGCACACCCAGGCAGCATTCCAAGTTCTAGTATGTCCATCTCAGGGCTGGGAATATAATCCAACAGGGTT
CTGAGATATTCTACATTAGCTTTGTCATCAGCTCAAACATCTCATGTATTCACTACTTATATCTTATACAGCCTTTGTAGCAAG
CAGCACAAGGATGTATGTACTCGACACTGACTGTTTTGACAACCTGTAGGTTAAGCACTGCTGTGAGCCGTTGATATAACACT
ATATACATAAATGGCAATGTACTGTTCTTCTAATATATTCTTCTAACATATATATATATATATACACATATACGTACATCATGG
CTAATGGCCTCACCCTGTATTCGGATCAAAGGCAAAGAGGCAAACAGTATCTAGTTGGAGATAAAATTAATCACTTGGTCTT
CTGCCGAATACCTCTTATCCGGTTGGCTGGCACTCATTTTGAATGTACTTCCTAGCAGTTGGCAAATACACAGAGACGAAATC
TAACAACACTGCTCCATTATCATGGACATAATAAGGAAATCCCATAATGATTCTATGTAGTGCATAATGAATGCCTATTTATA
TATTATGTAGCACTGTAGAGTATCCGATGGCACAGGAACAGCACACACAGAATAGCAAAGCTGCAAGCTCCTTAAATACACA
GTTACATAACACACTGCTATATGTCAATACTCCAGTCAGCAGGAGATGTTCAGCAGATTTCCACTCCATCGCCATCTCAGAAA
TTAGTTTGTGACATTTAAAAACATGGACATTATATTTTTGTAACTTTACAAGCTCTATACTATATATATATATAAATATAAATA
TATATTACCAAGGACAACACCTGTCTCCAGGACGCACCCGTGGAGCAAACCCTTGTGAATATATTATGACGCTGAAGATTTT
ATGGAGTTCGACGCACTTTCTTAAAAAAAGAAAAAGAAACTAAAAAGCTTCTGGATATTATCACTAACGGCTTTGTAGGAAG
CACTTTTAATGTTTTCTCTCACAAAGATTTGAACAACAAAAAATGTGCATATATTTATTCTGTAATTTCAGTGACAATTTAATT
GTAAAGGTAATGTTAAATCAATGTATAGTGATTATGCGTCTGGTATAGCCTTCTTAATAAAAAGTATCAAATGAAATAAAGG
GTGCTAAAATATATATTTGTGACTAACATTGACCTGTATTCCCTAGCAGGTAATTGAGGGCAAATGTTTAATGGAATGTGGTG
GAAAAAAGCTTTGATATTCTGAGTATTGTAGTGTGATGCATATCTTCTAAATTGAGATTGTGATTTTGGGAAAATGCAATGTT
TGGGAATGTCTGATGTGTATGAATTAGTAATTATAAATTTAATATATATATTTATACACATGCGATACATTTGATATCAAATT
CATATAATCTGATGGCTACATTTACTCATTAATGCAAACGCCCATAGAAATTAACTGAATTCAGGAAGTTAGTTCCCATGTCA
ACCACAAGGGGGCGACATTGAGACACTTATTTCCCTCTCCTGTCCTGTTAAATAGTTACTGCAGTTCATCAATGTCAGCTGAT
CTAGCCTACATAGCTTGCACGTTGGTGTAAAATGCAATGAAGCTCTTTCTGGGGCAAGACTGGATAGGATGGGATTATTTAA
GAGCTGAGGGCGCACATAAAGGGTTATGACAGGAACAAGGTTGTGTAACTATTATACAACAGTGAATGTATGCTGCCTAACA
GAAGCATTTTTGGTAAGTAATGCAGGTATATATCGGCCTCTTCAGTATCGCAATGATAAATTTGCTTAGGTAGCAACATTGTT
ATTAAAACCTTACGTTGTTGGCTTTGTTTGCGTATGGCCTGATGGTACCTTAGGAGATGTAATGTGTCAGCCCTAATGCCAGT
TTGCCA

>Cygb2_Ptd_TR62729|c1_g1_i1

TGGTTTACTAATGGTTATAGTTAGCGTAGGTGATAAAACAATCACACGGTCTTCTCACCATGCAGGACATGTGTATGGAGTGT
TGAGTGTATCTTGTGGCCTGTGTAGGTTCTTCCAGCGCTTCCCCTCCGCCCAGCAGCTCTTCTCCCAGTTCCGGGAGACCCAG
GAGCCAGCGGAGCTGCGGCAGAGCGTGGAGCTGAGACGACACAGCCGCAGGGTGATGGGGGCACTACAGGGCGTGGTGGG
TGCTCTAGAGGAACCGGGCAGGATGGGCTCTGCGCTGGAGCAGATGGCACACTCTCACGCTGTACGGCACGGAGTTCAGCCG
CACTATTTCCAGATGCTGTGCGGGGTGATCCTAGAGGTGCTGTGTGAGAAGCACCCAGAGAGCTTCAGCGTGCAGGTCCGAG
CGGCGTGGAGCAGGGTGATGGAGCTGCTGCTGTGGCATGTGGAGCAGACCTACACACAGCTGGGCTGGAGCCACTAAACAT
CTGCTCCTTCACAACACTGTGTGTGTGTGTGTGTGTGTGTCCTGCAAATTGGATGCTC

>Ngb_Ptd_TR2814|c0_g1_i1

GGATGATTGCTTGCCTGGATCACGGGAACAAAAGCGACAGTGGCAACGAAAGGAGCGTGAAGGAGAGAGACAGCAGCCGG
AGTGACAGATGAGCGCTGGGGCTGATTCCTCTCTTCACTTGCACTGGTGACTCGAGTGGCTTAGCACACGGCACTTGAGGGTT
TCTGTCAGGTAGAGAACTAAAGTGCAACCTTATTCATCAAGTTTCCTGGAGGACCCTCCAGGTGTAATATGGGTTTTGGGGAT
CTACAGGTCCTATTTTAGTCCCAGAAATTTGCAAATCCTGGAATAAAAAGCATGGAGAAGTTGACAGGAAAGGAGAAGGAG
CTAATCCGTGACAGCTGGGAGAGTCTTGGGAAGAACAAAGTCCAGCATGGGATCGTGCTGTTCACCAGGTTGTTTGAACTGG
AGCCAGGTCTACTCAATCTCTTCCGCTACAAAACCAACTGTGACTCCCCCCAAGAGTGTCTCTCCAGCCCAGAGTTCTTAGAA
CATGTGACTAAGGTGATGTTGGTAATTGATGCTGCTGTAAGTCACTTGGATGACCTGCACTCCCTTGAGGAATATCTGCTCAA
CCTAGGCAGAAAACATCAAGCAGTTGGGGTCAAGACCAAGTCTTTTGCTGTGGTGGGCGAGTCGCTACTCTACATGCTCCAG
CAGTGCCTGGGCCCTGCGTACACCACCTCCCTGCAGCAGGCCTGGCTCAACATGTACAGCATCGTGGTGGAGGCCATGAGCA
GCGGCTGGAAGCGGAATGGCGAGTGTGACAGTGACTGATCTTCGGATAGTAAGCTAGCTAGCAGGGAAAGAGTATTGTAGG
GCACAGCCTGGCTGGCCTTGCGGGCAGGCATTACAGCAGCTGGCAGGTTTTGGTGTATGCGGCAGGGACTGATATAGGCATA
TGCTGAAGTCCAGCAAGTCTGGCTGGCAGCAGATGGTATTTACATTGTACATGTCATGGCTGTATTGATCTAGGCAAAGGTTC
CTCTCCCATTTCTAAATCTATCTTCATCACGCTGGCCCAAATCGTGATACCATGAGTGGTATTTTAATTGTTCCTGAGCCTTTT
TCGCAGAAGATATGCTGTGCAATACGTACCGCTGTTGTCTCTGGGACACATAATTGTGAGCAGCTCCAACTTTCAACTTTGTG
TTTTTATGCTTCAAGCAGTGGTGTTTAGTCTTCTCATGAAGTGCTTTACTTTTACTCTTGTAGCTTTAAGTATTCTTGTAGCTAT
AAGTATATACTGAAAATTTTAATTGCTGTTGGGTATACTCATTGTTATGAATGCTACCTGCACTTTGCTTGCTTGTAATATCAG
TAGTTATTAAACTAATTAGCGTGTTGTTATTATGCTAAAAGTAATGGTGGGCCAGTATTTGCCACAGAATGCTTTTCAGTAGA
AAACATAGTATTTTATTACCTAAAAATTATGTAAAATACTCTGTGGCAATATTAGCCATTTAAGGTCCTTAAGTAGCCGTTCC
ACCTTGACAACTGTGAAACAACTGAAGAGATGCTTTGTTGTTCTCCACAAACCAACACAGCTTAGATTGGTAAATATTAATTG
TGTTCATTTGTCCAAAAATGTGATTATTTACCTTGAGGCACACAGTGTAAAAATTGCCTTTCTAGCTTTTATGAGTAATCTATG
ACTGTTAAAATATCCACTGAGCGCCTTATGTTGAAAGGCTACAGAGACGCACACGCATCACTTTATCACACTCCTGCTGACTG
TTATTTCACTTTGTTTAGAAAGACTGTTATCACTTTACCAGGGGGTATACTATTATACATCACATTCTTTTTTATGTTGATCAG
TTACTATTATTGTTATCTATAATACATGGGCGTGTCCCCATTTTTTATAATTAGAAATTGAATACTATGTCACTATGTATTTAT
TATTCTAGCACATTTATGATCAGCTGGGTGAAAGCAAAGGCATCTCAGTTGCTATATTAATTCATAACCAACATAAATGGCCA
TCTAAGGTATTTCAGGTTATATTCTGACCACAAATACAATCTACTTTTCATTTATTTCTTGTTGTGGAACTACTTTTATAAAGA
TTATTTTTGTATCTTTGAAATGAATGCAATTTGTTTTGTCTTTATTTAGGTGTTTGGTCAGACCTGAAGGGAGGTCAATACGAA
GCACATACTTCTGTGCACCACTCTGAAAAATACCCTTTAATGAAGTTTTAAAATGTCATATTAAATTACAGCAATTTTCCCTG
CTTTAATTACAGTGATTTGGTGTGATTACTTTATCACCATATTTGCTTTTTACGTGAAGCTTTAAAAATGACAACCCGTACACA
GGAGTTTAACTACATCCTTAGTCTGTAACTCCTCAGAGCTCTTCTGACTGATGTGTTATTAGCAGTTTTTACCCAAAATTGGTT
CTGCTCCAATTTAGTAACATGGATTGGAGCTTTTAGAGACTTTTCCGGGTGAACACATGCTCAGATATTTTGTAGGTAAATGC
TTCAATGTCTACCTCATCACTGTAGCTGCTATGGGATAGTTAGCCAAGTATTCTTCTGAGCATCGTTGGACCAAAAAAAACAG
CAAATACAAATAAATACAACAACGCAAATTCTTATCAGGCAGCTTGTGAAATGGATGTGTACCACTGAG

>GbX_Ptd_TR77111|c0_g1_i1

CGCCTGAGGAACACACCGGCACGGAGGCGCATCTCAGCCACACACACATCACACTGCTCAAAGACTCGTGGAAAGTCATCC
AGGAGGACATCGCCAGAGTGGGCATCATCATGTTTGTCAGGCTTTTTGAGACCCATCCTGAATGCAAAGATGTGTTCTTCCTC
TTCCGGGACGTGGAGGATCTGGAGAGGCTGAGGACCAGCAAAGAGCTGCGAGCTCACGGACTCCGAGTGATGTCCTTCATTG
AGAAGAGTGTGGCCAGACTGGATCAGTTGGAGAGGTTAAACCAGCTGGCAGTGGAACTGGGGAAAAGCCACTACCGCTACT
CCGCCCCACCCAAGTACTACAGGTACGTTGGTGCGGAGTTTATCGCAGCTGTCCAGCCCATCCTGAAAGAGGACTGGACCCC
AGAAGTGGAGGAGGCATGGAAGGCTTTGTTCCTGTACATAACCCAGTTCATGATGCTGGGATACCAGGAGGAAGAGAAAAA
CCAGCAGAGCAGCCCGGCCACATTAAAGCCGGAGAGACTGGAGAGGGCAAACACAGCTCTGTAACAAGCCAAGGCTGGAG
ACCCCAGTTTGGACTGTGTGCCCAAACAAAAAAATCAAGCTCAGGTAGATCAGAGTGCAGCCTCAGCTTTTTACATCATGGG
TGTAAAACCACCACAAGCCCTCGGCTGTGAGTCCACTTAAATGAAACGAGTTAAACGAGTAGTTATGGTGGCTTTTCTACATT
CTGATAGATGACCATTAACAGAATTGTGTGTACAAAGGGCCACTTAAGCAGCAAATGATAAACAATCAGAGGAACAATGTA
CAAACAAACCGATGAAGAAAACCTTAATAAACATTTTTATGCTAGTTTATCAAATAATGATGCGGCTCAGAACGTCCACACA
AGCCACAGCGAAGCTAAAATTTACTTTAAAGTATCTCCATCATCACCAGCTCGGCCTCGGGCAATAATTACTGACAGAGGTC
ACAAAAGGTCACAGGACAGCAAGCCAGCAAGTTCCACTTTTATTGCAACCCAAATGCCACCAAACTTGTGCAGTTTAATTCC
TTGTTAATGTGAACATGCCAGACACCACGGCTGGATGTGGACCGTACGGTGCAGACTTGAAACATACACGCCATAATTCCAC
TAAACTCAGAAAGGTGGCCTTCTTGAGAAAGCTAATCTTTATTTTCAGAGTCATAAAAACAAAACTTGCGTTATAGGGAATT
CATCAACGACAGGTTTGTAAACTAGACGTGATCAACGAGGCGGCAACTCTACCGTTTTGACAAAAATCCAGTCGTTGAATCA
CCTGATACTTTCTGGTCATAAAGAAACTCAAAGGCCCTGAGGCCTAGGATTGAGACTCTGCTGGCACCCAGACCTGGACAAC
AAGACTAAAAACAAGCAATTAGCTCTTACACAGTTCACCCAATTTTAATGCGTGGCTCTCTAACAGGAGCACATATAAACCA
AAACGTACCAATGCGATTTAAACGGAGGAAGCGGACCAGATCAATTAAGTTCCCTTGTGTGTCACCCAGCACACGCCCACTA
CATCGGTGGGGGGGGTTCCTTTTCACGTGACTCTCAGAAATCCCTTTCAGGCCACTTTTGGTCACCGTGTAGTTTTTGCAGCC
ACATTCGAGTCCCATTAACCCGTCAGAACTGCACTGTGTGACGGTCAGCTTCAGTGACGAGAGCAGCGCCGCAGTGTCCATC
GTTCTGAGAGCAGCGTATGACGGCTGGGTGGAGGATTCCTGTGTGCGTGTGTGTGTATGCGTGTGTGTGTGTGTGTGCATGTG
TGTGTATGCGTGTGTGTGTGTGTGTGCATGTGTGTGTATGCGTGTGTGTGT

(c) The full globin gene family repertoire found in the Osteoglossiform species *Osteoglossum bicirrhosum*

>Mb_Obi_TR81226_c0_g3_i1

TTTGTATTGTGAGACAATACAGTGGGAAGGGGGGAAATGGACTGATAGGTGGGACGTAATACATGAGGGAAGCTGGCTGCC
TATATAAAGTAATGCTGTGGAGCCGCAAAGACTAACTCAACTTTTGGAGTTGGAAGGAATCGCACATCTTCACTTGGGACTG
CATTCATTTGGCCGCTACGATGTCCGACTACGAGAAGATCCTGAAGAACTGGGATGCTGTTGAGGCTGACCCGAATGGAATC

GGCGGAGAGGTCCTGTACACTCTCTTCAAGGATTACCCAGACACCTTGAAATACTTCCCGAAATTTGCTGGTATCCCACCCAG
TGATCTGGCCACCAACGCACTGGTGGCGCAACATGGTGGGGTGGTGGTGAAGAAGCTAACCGAGCTGCTGAAGGCCAGGGG
AAACAATGCGAGCATCCTCAAGCCCTTCGCCACCAGCCATGCCAAAACACACAAGATCCCCACCAGCAATTTTAAGTTGATC
ACGGAGGTCATTGTGAAGATCGGCGCAGACAAGGGGGTACTGGACGCAGCTGGGCAAAATGCCTTTAGGAATGTGATGAGC
TCCATCATTGCTGACCTGGACACCTACTACAAGGAGCTGGGTTTCCAGGGATGAACGTAACCCCGCTAACCGAAAGCGCCAC
AGCCACGACTAACGACAACTTTAACCTTCCCTAGACCTTGAATTTTTAATAGAATGTCTGCAGCGGTCATCTTTTTAAATCTTT
GGGCACAATGATATTTCAGTTCTACTGCTGTACTTTTCACTTTGCCAAGTGGTGGTTACATAAGCGGGATTAGCTATATGATT
TTATACGAGATTGCAATATAAAGAAGAGGCTAACGTGAGAGAGGGGTATTTCCGAAGGGAAGGAAGATGGGAACTGCTTTT
TAATCTTAAAACGGATTTGAAAATTCATCTATTTTTTTTTTTTTTTTTTTTTTTTGGGGGAACCCCAAACCTTTTTATTTATCA
AGCT

>Cygb_Obi_TR65882_c0_g2_i1

GAGAGAGTGTGAGGGTTGGGTAGGTGGGTGGGTGACACAAGAGTATTCGCCGTTATCCCTGTCAGGTCCTCAAAAGCGCAG
GACACAAGTTCAGAAGGAAGATCGGTTCTGAGCAGGAGAGCACGGTTTTGAGGAGGGCGCTGTTAGAAGCAAGTCAAGAAA
GGGAGAAAACATCAAGGGGAGAGGAGCAGGCGGAGGTGGGGGTGCTGTTCTCTTTCTGGATCTCTTGTCTTCTCCTGTCTCC
GCGTGGCTGATTTTTTTTTTTTTTGTTCAGCCACGTTGCTCCCCTCAGTGCCACTGCTAGGAGGGGGGGGGTCGCACATGGAGA
AAGTGCCAGGAGACGTGGGTATGGAGCAGAGGGAACGTGCGGACCAGCTGTCCGAACAGGAAAGGGGGATGATCCAGGAC
ACCTGGGGACGGGTCTACGAAAATTGCGAAGACGTGGGCGTCTCAGTCCTCATCAGGTTTTTCGTCAACTTCCCCTCCGCCAA
GCAGTACTTCAGCCAGTTCCGGGACTTAGAGGACGCAGAAGAGATGGAGCGAAGCCTGCAGCTGCGCAAGCATGCCCAGCG
GGTCATGAATGCCATTAACTCCGTTGTAGAGAACCTCCAAGACCCCGATAAGGTGTCCTCCATCCTAGCCCTGGTGGGAAAA
GCGCACGCTGTCAAGCACAAAGTGGAGCCTATGTATTTTAAGATCCTCAGCGGAGTGATCCTGGAGGTGCTGGCTGAAGATT
ACCCCGACTCCTTCACTCCGGAGGTGCAGAGGGCCTGGGCCAAGCTCATGGGCCTGGTGTACTGGCACGTGACAGGAGCCTA
CACAGAAGTTGGATGGGTTCAGCTGTCCAGCTCCGCCGTCTGACCCGGGGGGCCAGACCCAGGCCACTCCAGCAAAGGCTGT
GCGGGAACCGGCACGGCCACACTGTTTTTCTGCTGGAGGAAACAGGCATCTGCTGACAGGCCAGTCCATGCGGGAGGAGAA
GCGGAAGCAAATTGTGAACAGGTGGTTTGGTTGGATGGTTGCAGACAGGGAGGAGCTGTGCACCTTACGGGAAATTTTAAA
AAGAAATATCTACTGCTGGTATGTTCTTTCTCCCAGCCAGGTTCAAGGGCCTTCCCTTCTTCCCACCGTAAGATCTTTGGGCTT
AATTGTAACCTTTGGGTTGTTGTCTTTTTTCTGTATGGTCTGTGAGTGTATTTGTAAATACGCCCTCATTCCAAATCTCTGTGA
GGTCCATTTCTGCAAAGGGTATTACAGATTTGGCTACGGAGACTGGATCCGTCGTTCGCCTGAACACACATTTCCCCCGTCCC
CCTCACCAGCACATCACTCTCTATCTTTCATGCACCGTAAACACACATACAGATAAGCACATTTCACACAAACAAATTCCTCC
TGGAGGCAAAGAAAATTCTCAACGCGATGGAGAGTGACATAATATTACCGGTTACCGACAGTGGGTTATCTTATTTGATCAT
AGAGCAGCTAAGTTTTCGTCACATGACTATGTACAATGACCCGGACGACTCCGAAATAGTTTTCTGACAGTCCTTGACCAAA
ATACTTTTAGCTGAGCGATGATTCATTAGTGACACGGCAACAGCTCGCATCCGTGGAAGGTTAAAAAACACAAGTGTGGACC
CATATCCCATTTTAGCACTTTGTTTTCAGTGAAATCCCAAATAAAATGTGTTTTGTTTACGGGGCGGACGCAATTGGCGTGCT
GTCAATCAAAACAAAGCAAAGTGCTGCAGCCAAAAAAAAAAAAAAA

>Ngb_Obi_TR76519_c0_g2_i1

CTTCACTTGTGGGGAACAAAGCCACAGAGCTGGAGAGAGCCAGGGAGAGAGACAGCAGCCAGAGTGACAGATGAGATCAC
TCACCTTGTGTCGCACCTCTGAATTAGAGAGCACGGGTGGCAGGTGCACCCAGACAACAGAAAGTGTGGATTCCCCAGGGCC
GTCATCCAAAAGGAGTCAGTGAAGAGGATAATCTTCAGAAGAGTATCCAGGTGTATTATGGCTCTGGTTCTGGTCTACCAGC
AAGATCCACGTTGACTCTGCAGCACATTGATGCGATTCTGACCGTCATCTAGAGGGGCTCGCGTAACGCCAAGCATATGGAG
AAGTTGACGGGAAAAGATAAGGAGCTCATTCGCGAAAGCTGGGAGAGTCTTGGGAAGAACAAAGTCCCACATGGGGTTGTT
TTGTTCACAAGGTTGTTTGAACTTGACCCAAATTTGCTCAGCCTGTTCTCTTACAACATGAAGGATGGATCCATGCAGGACTG
CCTTTCAAGCCCAGAGTTCTTAGAGCACGTGACGAAGGTGATGCTCGTGATTGATGCGGCCGTGAGCAATCTGGATGACCTG
AACTCTCTGGAGGACTACCTGATCAACCTGGGGAGAAAACACGAGGCTGTTGGTGTCAAGATCCAGTCATTTGCTGTGGTGG
GGGAGTCCCTCCTGTACATGCTGCAGCGCAGCCTGGGCCCAGCATACACCGCTGCCCTACGCCAGGCTTGGCTCAACATGTA
CAGCTTTGTGGTGGCGGCCATGAGCAGTGGTTGGAGGGAAAATGGTGAGCGTGACAGCGACTAAGCCCATAATCCTGCTGG
GTGTTAATCAACCACTGCTAACATGGGCGGCGGGCGCTTTGTGCCGCTTCACATATGTCCACATCCAGGGCTGCTGCGGGGT
ACCGCTCTGAGGACAGAGAGCAGGGTGAGCGGCAGCCAAAGCGCTTTCGTTCTGATGGCAGCCGTTCAGCAAGCACACAAC
GTTCTCGCTAATGTATTGGAGCTATGTGCCGTCGCTACAGCTTTTATAGGAACGAGGTGTGAATGCTAAGTGCTGTTGGTCGT
GGACACATGCTTCATCTGCAGATCACGTTAAAAATGATAATCAAGTAGAGATGGTTGGGGATTGTATATGCTTATATCTATAT
ACATATATATATATATATATATATATATATAAAACGGACTCATGTACCGGCA

>GbX2a_Obi_TR21316_c0_g1_i1

CGGAAGGGAGCGCGAGACGCGGCGGATCCGGCGGTCGCGCGCCTCGGCGCACAGCAGGTGCACCTCATCCGAGAGTCGTGG
AAAGTGATCCAGGAGGACATCGCCAGAGTGGGAATCATCGTGTTCGTCAGACTGTTTGAGACCCATCCAGAATGCAAAGATG
TCTTCTTCCTGTTCCGACATGTGGAGGACCTGGAGCGGCTGCGAACCAGCAAAGAGCTGCGAGCACATGGGCTGCGGGTGAT
GTCCTTCATCGAGAAGAGCGTAGCCAGACTGGACCACATGGAACGGTTGGACCAGCTTGCCCTGGAACTGGGGAAGAGCCA
CTACCGTTATAACGCACCTCCAAAATATTTTGGCTACGTGGGGGCAGAGTTTATCTGCGCTGTTCAACCAATCCTGAAAGAGA
ACTGGACTACAGAACTGGAGGAGGCATGGAAGACCTTATTCCTCTACATCACCACAATAATGAAGCAAGGGTACCAGGAGG
AGGAGAACCATACCAACAATCTTGCTGCTTCCAGACGGGAGAAACCCGAGAAGATGAATACAGCACTTTAAATTAAAAAAC
CTTCACTTTCCTCTTCACTCCTAGAGACTGAGTGACTAATACTGGAAGAGAACATCAGTCTACCG

(d) The full globin gene family repertoire found in the Osteoglossiform species *Chitala chitala*

>Mb_Chitala_NODE_225554_length_9747_cov_10.196983

CTTTAGTATTCAAAATTTTGTTTGAAGGGGGCAAATGCTTTACCACAGTACTGTATATGAATTAAACATAAAGGCTTTGTAAT
ACAGATGGTATCAAAGTTCTGAGAGGCCTGATTAAGCAATGCAATTTTTTTCAGTTGTAACCACTTACATATCCAGAAAAAC
ATCTGTAATTGTACCTGCAAGTCATATTGAGTGGTACAACTGATGCAAAACAAGAACAATGTCATTGTTTATCAGGCAGTTAT
CACATAGTTAGACATTTTCTTAGCTTTTACAGTTATCAAACTATTTTTAACATTTTTTGTTTATTTTGAACAGGACATATTTAG
GTGCTGTTCAGGTATAAACCTGTATTATTGGGAGCTGGTAATTATTTTCTAATTATCTTTTTTAAAGAGTTATTACCTAATGGA
AGATTTCTGTGCTTTGTTTTCAATACATAGTTTTAACGGTATGTTAAAGTTAAAGTTCATTTGTTGTCCGTCATGGAAAAATGT
ATCACTTAACAACTAGTTAAGATGTTTTGTTATTTCTGTAGCTATTAAATTGTGTTACTTATTGTTTATTTGGTGACGAGTCTA
CGTATTGTAAACTGCTCTGTCATACATAACTTACATATTTTAAAATGTTGCTAATGTAATGCTAAAATGCCCATACTACTTTAG
TTTGTTTCAAAGTGCTGAAATTTTCCTGAAATGGGGTTACATTTACAAATTTCATTGTAGCATTTCTCCTGTTTTGAATACATT
GCTGAGATTGAGGCTTGTCCGTTTTTAAATATTGTTGACATTAATTATGATTATTCCTGTATGCAATCAGTAGAGCGTGTGTAC
CACAATTGATTTGCTTACATAGTAATTTTCCATGTCAGTGGCCACTAGAGGGCTCCTGATGCACAGTGACGGCTTCTGATTTC
ACTCCCCTGCACTGCAGAGACTCGAAGGCACTGTTACTGTGTGCATGTGCATATTCTCAGCCTTTAACCAAGAAGGCTTGCAG
TAGCCAAGTCTGACCCTGTTGAATTTTGCTCTGTTAAAGGCTTCTGTGCACATCCAATCCAAGTGAGTAGTTTAGAGCATAGA
TGCCTTGTTCTGTAATTGTATCTCTGCCAATGTAGTAGATGCCAGTGGGATCCCTGTTCAGTATAGCCAAGTTAACAATCAGT
ATAATTATGAATAAAGAGCTCACCAGGGGATTAGTTTTTCGGATGTTGCTAGATGCTGTGTTTGGTCTATGGCTTTTTCCGCC
CTTATGCTGAGATCCTGCTTGAGGTGTTTTCCCCCTGTAATTCCCTCCAACCCCTGATCTGTTTGTTTGTCTTTGTTCCCCCCTA
CCCAGTCCTTCCTTCCATTGTAACAGTTAGAAACCCTTTGCTCCTCCTGTTCAGTTTATACTATAAGGGTTAATTGTGTGGTTT
ATTTTTTTCTATATGACCACGGCCCAAGTGTTATGTTAATGTCCACCTTTTTGCAGGTAATGATTAACTTGTGAAAATTATACA
CTAATTTTAGTTACAAAGTCTAGTATTGTGAGTTCAAATGGAGCTGGATATGATATGCATGGTATGCTGCAAGTGATATTTAT
AACATCTACACAATAACTACTGAGATCACTTGTACCAGTGTTACATGAGGGGTCTGTAAACTGGATTTAATCAAGTTCCATGT
TGTAAAGTTACCATTCTTGTAGAATTCTGACCCAGGAGGAGGATATTACTATTATAAACTTTATCCTTTAAATGTTTTCCCCAG
TCTGCCTCAACCTGCCCTGCTTTTAAACCTATTAAAAAGAAAATGTGTGTGCAGCAGTCAAATTGTGATGGTGAGGTGAGGA
GAATAATGGAAACCACAATGGCAGAAAAGGTTTCATTCAGCTTTGCGTTGCTTGAACTAATTTGTAGTAAAATAATACCACT
TTTTCATTCCCCCCTCCAAGATGATATTACAGTTCTAATGATTGTTTGGTTTTGTCATTTACACATTTAGTCCCAGTTCTGCAGT
CCAAAAAGAAAAGAAAAGAAAAACCTGACAGGAAACATAAGTCGTTTTTATTTTCATTCTCCTAAATCTCAGGGTATTTGGA
AATATTAAACCAAGTATTTTGCAGCTGGTCTGAGGTTAAGACAAGCAGGTCAGTATCAGAATGGGAGTAAATGCAAGAGTG
AAAAGGACTGTTGAATAATTGAATGCAGAACATAATGGTTGACCAGAATACCAAACCATTCATCAACCCTGATAAAATTTCC
ACAGTAGTCAAAATAGGAAGTATGAGTAGGGGAAACAACACTAAGAGCTAAAGGCACAATATGTGTAATATAACATAGGGG
GAATGTGTAGTTTGAATGTAAAGTGTGGAACAACGGTGTAATTCTATCTGGGAGGGAGGGTAAGAGGTGGAGTTAAATATTT
TAAGACGAAAGAAGATGGGGGAGAGAGAGTGATTGATAGCTGGCATGTGATAAATTGGTGAGGCTGAGTAAGTATAAAAGG
TAAGCTTTGTGAACTGCAAAGGCTAAAAGGTACGCGAGGAGTCTGACACACAACTTCAGTTAGGAATTCATCTGAGTACCTT
GTACGGTATGTATGGACATTGCTGTTTTAATTTTGTGCAAAATTATACAGGTCTGTATGCAAGACTTGTTGAAATTATATATG
GGGAGGTTGAATAGTGGGTAGAAATCTGAGAATTGAGTCAATATGCCAGAGGAGGGATGACACCGTTCACTTGTGTTTTGAA
TTTTATTGACTAATGTACATTTGAAAATAAGACTGTTAAGGACACTTAAAGGTCATCAGACCATGCACTGGGACCAATAATTT
AGAATGCTCTGCTGGCTTTATTTATTCCGAAATTTGTCTTGCATGTTTAACAAGTGGCTCTTCACAGCCAAGCGTTCAAGACG
TGAACAAACTTACATGTATTACTCAGTAAAAAAGCTATAGTTTACATCCTTTCATTTACCTATTTGCAGGGGGGGTTAATGAA
TGGTACAAATGTAGTATTGCACACACAACTTTAACATGCAGGGATATTGAGGTGTGTGTTTATATGTATGCAAAAAGTGCAG
AAGAAGAAAGATCTGTGCTCAGTTCTGGTGTTTTTCTCTAATCTTTAAATTGTCATCTGTACCCTACAATTCTAATGCATTTTA
AGTAGTTAGTACTTATAGTTAGTACTTATTTTGGTGTAAAAGGTTTTGCACAAAAAACAACAAACAATCACCCCCTAAGCCAT
GCAACCTCTGATCTTCTCATAAAGAGCAAGCATATAATAAAGTACATCATGCATTACCTGAGGTGGTTCTCCTGTCCCACATT
TTTCTGCAGAAAATGACTTTTTCAGGTGTGCGTCATTAAGCTGAATACAGTGTTCGGTGATGGTGTGCACGTAATACGCAGCA
ACGAAGGTGTGTGTTCATTACTGTTTTAAACAAAGGTGGTAACTTGTGTTTTACCCTTTCAGAAAAATGTCTGACTTTGATGTT
GTTCTGAAATCCTGGTCCATTGTTGAGGGCAACTATAAAGCATATGGAGCGAAGTTCTGACCCGGTGAGTAAATCAGTTTA
GGAATCTGCTTGGATTCTATTGTGTGTAGGGTTTTTCTCTTTTTCTGCTGATAAAACCGTGGTCCCCAGGTTTTCCTACTCTGCT
TCTCAGAATTTGGATAAGATGTGGTGGTCTATAATGAGCCATTGATGCTTAGTAACCCACTACCTGGTTGGGTTATCAAAAAA
CATGTATACATGCTGCATACATTTTGGTTCTCCCTTAGATGCCTTAGGTTACGATGTATGATGACAACTACAGTAGTTGGTTG
GTCCAAGACTAACAGTCTCTCTGAATGCAGTTTGTTCACAGAACACCCAGAGACACAGAAGGTGTTCCCCAAATTTGTTGGG
ATTGCAGCCTGTGATCTGCCTGGAAATGCTGCAGTGGCGGCCCATGGTGAGGTGGTGCTGAGGAAATTGGGTGACTTGTTGA
AGGCTAAGGGGAAGCATGAGTCCATCCTCAAGCCCATGGCAGCTGACCATGCTAACAAGCACAAAGTCTCCATGACCAACTT
CAAGGTGTGTGGCTGTAGTATTTGAAAAAGCAATAATCGTGTTTGGGTAATACAATTTCCTTCTTGCGTGTAGCTTGTACTTG
TGTCTTTAGCTCTCGTGTCTCATTTAATGTTCTGAATTCTCTCCTCTATGTTCTCACTTCCTCAGTTGATTGGTGACATCATTGT
GAAGGTGATGGCAGAGAAGGCAGGCATGGATGCAGCTGGTCAAGCAGCTTTCAGGAATGTGCTTGCAGCAGTTTTCTGCGAC
ATAGAAAAATACTACAAGGAGATTGGGTTCCAATGCTGAACCACTCTATTACAAATTCTGTGTAAGATTGTAGTAATTAATG
GCAAGATTAAACCTGAAGCTTCATTGTCCTATTGATTTTATGAATAGAAATGGCCATGACCAGAAAAAGTATAGATTAACAC
TGTTACAAGTCATGAGTTGCATCCTTTTCCTTAAGGGGCCTTAAATCAATATCTATGTATGCATTCAAACCACAGTGTTTGAA
GAACTAATTTTTTCCAATGTGTTTCTCATTGTTCTTCCCATTTAACTTTGGTTTATGTTGTCAAGAATCATTTGCTTTAGATGTG
TGTCATTTCCAGCATTACTATTAAAGGTTGATATACCAGTTAATCTGTTTTTTCATCCTTTATCCCGTCACAAAAAACATCAAT
GCAATTTTGTATCTGTGATTATCAGGCCTACAGTACTCCATCACAAAATGAGTGGCTGATGGGAATATTGGTATAGCTGTCAG
TCAATCTGACTGACTGGCTTTTGCCCCAGTATCTTTTTGTGACCATGTAGTCAGGCAATGCCAACAAGCCTGAACTTGAATTG
GGCACCAAACTGACCTGCTCCACGCAGCCATTCTTGCAAATAGGCCTACTGCATATTCATTAACACAGATTAATAGATGATAT
AATCACATGTATTACAATTTGAGGGAATTTATACAAAATGACCATGGAGACAGTTTTGGTTCTTCACCTACCATGTAAGTGTC
AATGGGCAACACGATATGTTCTGCTACATGTCACAATACCACAGCGGGTAGTGTCTAACAAAATAAAAAGGGAGGGTTTGGT
GATGGCATCTACTCTAAATGGGAGGATCACCTTTCCAGCCACATGGTGTTCATCGTGATGGTGGAACATTTGCCTGTCCAGCA
TTTCCATTTGAGATGGTGCAAGGTCTATTCTGGTTCTCACCTTCTTTGCCCTCAGTCATTTCCAAGTGCAGGCCAAATGCTGGT
GACTTATTTTGCATGAGGTTGTCCCTTAATTAAGTTAAGGGTGCTTATAGCCTCTTCTTCCCATGGTAACTGTTAAAAATTTTG
CTGCAGGTCATACATAGGCATGACAGTCCCTGTCTCAGGTTGCTGTTACAGGTTAGCAAATGAAAAGTTGTACACATTTCATT

TCTGTCTCACTGTATCAATTCAACTACATGATGCTGCCATTGTCTTCTTTAGCATTCTTTCATGTGTTCTTAAATTATATGTAAG
ACTAGGGTCCACCACTTGTACTTGTTTTGTATGTGGATTTGTAACAGTTTGGTGCTCTTGTGTCACACTCTTCATCAACACAAA
GCACAGGCTCACAGGCAGTGAATTACTGCCATTTTCAATTGTCTTCAAAGAAAAAAAGTTTACATTCATTGCATTGTTCAGTG
ATGGCAGAAAACAGCATGATATTGAGCAATGTTTGTGTCTGGACACTATATCATCTCCATCCATGTGTGTCCAGCTGTGTTCT
TTATTTCCCCACAGGCACAAATGCAGGAAACCAGGGATTTGCGAATCCCTGCAGTCTTTGGCTGAACGCGAAAGAACATACG
GCACACCCATGATGAGAGCTATCAGTGTGAATTGTTATCTGTACAATAAATAGCCTTGAAAGAAGATGCCAGAATCCAGAAA
TATCACACAGAGAAAAAAAGACAGCATCAAGCACAGTTCCTTGAAGGACTCCAAGTGACCAAATTCAACCTATCTGAAAGTT
CAGGTCAGTCCCTTTGATTAAAAATGTCAGACGTTTTAGACACACATTGATGGAGAAATGTGGCCATATAGATAGAAGAATA
AAAACAAGGCCAGAGAACAGTGAAAGGTTCTACAGCTGAGATGTCATGAAATGTTCAAGTCCAATGAGTGTACATGTTTGTT
AAATTAGAAAAGAAAACAAGCACAGTTTTAAAGCGCTGCATGTCTAATAATCTTGAAAAGAACAGAAGACGATACTAGTTA
GATACAAGTATATTGGTATGTCTTTCTAAACCACTGAAGTCTGGATGTTTCTCAAGAGGCATGTGATGTGTTTTTGAAGACTG
GAAAAGAGCATTTAAATAGGGATTAAGTGAGATCAAATTACATGGTGTGAGGTAGAGTAGGAGGCTAAATATATGAGACAC
AGGAAAAGAAATAATAAAGGAACAGGAACAGGAATGGTGCTGGAGAAAGGAGAAGAGGCACAATGTTGAGACACAAAGTT
TGGAGAGAAACTTATCAAAGATGAACTGTAGAAAAAATAAGAGTGGCTAAAAAAATTAGGTGGCATTGAACCAATTGCAAT
TTTATGGCTCTAAAGCATACAAGTCTCATGTCACCAACACTGCACTGCTTCAGCTGCTTTGAGTGACTCTAAATTGGTCCTTA
CTCCCTTCCTTCTTCAGTTAGATGCTAAATTATTAGTCCAGCAGGTTACCTTCATCCATATATGACCCCACCAAATCCAGAATA
AAATAAAACATTTATCAGGATGGCTTTATTGAGCCCACTGTCAGCTGTATATCTATTTAGATCACTTTGATTTAAATTTGTTTA
TCATATTAATGAAAATTATATCTCTAAAAATTCAGATTCCTGTTGCTACTTCCATCATACACAAGAATAGGCTGTATCTGAAG
GCCATCAGCAAGCTATTTTTGTGACTTTGTAGTCGTCTAGAATCAAAAAGAAAAAAAAAACGTTTTCCATTTAAACAAGAAC
AATTACATAATGCACACAGTGTAATTAGGTAATATGCTTATTGAACCTGGTAGATCTATCTGGGTGTATCAGGTTCAAGCAAG
TATAAAACTAAGGGGATTTTAAATTAACAATATGAAAACGCTATTTGACAATTTATTTCATACCCACATTGTCAAGTTGCTGA
CAATGTGGGTATGAAATAAAGTATAACCTTACAGACAATGGACCCAAGTATGTTACAGAGGGCAGCATGTGACAAAATGAG
AAAGGTAGATATGGTGACCTCAAGTGATTTGTGACATATGGGTGGATCACCTCTCCACCCCCTGGCCTTGGTCATGCTGAGA
GAACTTCTGCTTGTCCAACATTCTTCATTCAAGCCTGTGTGAGGGCTATTCTGGTCCTAACCTTTCTTGCCCTCTGCTCTCAAA
ACTTGTCCTGTTGTTTTTCAGTGCAGGTCAACTACAGTTCAACTTTTTCTAGCAACCTGTCCTTTAATCTAAATAACTTATTATT
TGTAACCAATGCATGTTGTTGTAGCAACCATTAGAAACTAAAAACAAAAGCTGCAGTAATTTAAAAAATAGCCAGACCTATT
CACATTGGCTGTAAGTATAATTGTCCCATTACCCAGGTTAGCTGTGCAGTTTGAAGGACTTCCTGGAAAGCCCTGACCCTGTT
GCACACATTCTTGACACCTCGTTTACCCAGGCTCTGTCCTCTCCCTGTGCAGAGCATTCCCTGTTTCTCCTGAGGACCCTTTAA
GACTTTCCTGTTCCTCTCCACCATCCCCCAAAGCCCTCCCCACTGCAGGCTTATCGGTGACTGCGTACGTGACTCAGGACACA
GCAGCAAGCTTGCCTAGGGGCCTTGTGAACAGTCACAGCCTGTATGACCTGTACCACTATAGCATGTCAATCTACTTAAGGT
GACCACATGACACAGCTGCCTGCTCCTAAAACTAGACTAAAATTCCAGGAATGTTAAAAAAGTAAAGCCAATCATGGAAATC
AATGTACAAACAAAACACATTTCAAAAGAGCTGAGTGTTGCAAATAACTTACAAAACAGAAAATCCAGAAAGCCAGAAAGC
TTATAAAATTATAATAAATTAAGTGTTATTGTTAACAGAAAACTTTACAATGTAACCTCAGAGCCACTGAGTGGAAGTTTCGA
GGGCATAATTGCCACTTGATGCTCTGCCAAGGTAGCAATTGCAATGATGAATGGAAGTATTAATAGTATGTCTGGCTCTCTAT
CAGCACAGACATGACATAGCACACTGTGTTACCCTCTACCAGACAGACCCTGCTTCTTTTACTTTAGGGACAAGGACATAAA
GTCATGTATTGTCCCAATACAAGTGACAAGCATTAATAAAGAAAGAATATATATGCAGTTACAAAAAAAACCCTGAAAAGG
AAACGTAAAACATAATTAAACACAATTTTCCGTAAAACATACTATATTTCAAAAACAGTACAACCATTCCTCAAAAACGTTT
AATTTTGCACACAACCAAGAAACCAGCAGAATCCAATTAAAATCACTTATCTTATGTAGAGGCATAACAGCAGCTTTAAAAT
TTTTGCTTATATTTTTGGTTGCCTGTTGAAGCTGTCATTTCCTCCCCTCCCACCCTCATGATGAAACAACACGTTCAATGTTTGT
GCTTGTCTTTATGTTGTCATCTTTCGTGAGAGCTGCATCCGGCTGTGTTAATTCCTTTATGAAAGTGAGGTGAGACTGAAGGA
AACCAGGGACTCAACAAGTCCCAGCGGGTCTCCACAGAACGTGCAGAAAAACACCAGCGTGGCTGTGATGCTCCCTGACTC
AAACAGTCAAGTGTCTTTATGTTCTTCCCCTTTTGACAAAAACAGATATACACATGGAAGGAACACCCAGTCCACTTAATGG
AACTCATGCTGAGCACATGGAGTCATATATTTTACATCAACAATTATTAATACTTGTCTGATCACAAGCTGATTTGTAGCTAA
GTGGAACGAAGGAGAATAATATCCAGTCGTGTAGAGCAGTGTCACTTTCAAGCAGAACTTGAAAAACAGAGTGCTGATTTTC
CCATGAGTATAGGAATAGACTTGTCTGCCTCACTCCAAAATCTGAGAAAAATGTTTATAATGTGCCGCTCAGATATCTGAAA
GAAGTCAGAACTATTAAAATACAGTTTGGTTCTCCTTGCTTCCACGGGACATGCTGCAATGCTTTCCTCATTTGCTATGTCTAC
ATATTTGTCTGCAAACACATGTGCAGCAGTGCGCTACATTAACAAACACTTTTCACCAGCCTTCATGTTCTATACTGCTGGGC
CAACTCACTGATAGGGCAGGCAGCATGTAGTACATGCACACTCCACACCAACCCTGCACCCCCCCAACTCTCACTTTCTGCCC
CATCTATGTAGGCACTGAGCACTGAATGATTTAGTGCAACCTGACACTGGAACGGGATGTTTGTCCAAAAGATTGCTGATTA
AAAGTACAGGAGAAATTGCTATTGTAACCTTGAGCAACCTTAATTGCTGCAGCAAATACATTCAGCTCTATAAAATGTAAAT
CATGAAATTAGTCAAAAATCAAATAGAATTTAATTTGATATTTGACTCATATTGTATGAATATATGTATTTCGTTTTGGTGTTA
TAAATGTATAAAAATTACAAAACCGACTCAGTCTAAAATAAATAAAACTATCCAGTCCAGAATTATCACGAAACGCTTAAT

>Cygb_Chitala_NODE_440982_length_9268_cov_8.596785

CACTTTAGTTTTGTTTATGGGGTCTGTCTCCAGGCTCTATAGCCCCCCCCCCTTTCTCCATGCTTCTGCCGATGTGTGTTCCTCC
CAGGGGCTGGCACAGCTGAATCGTTCCACTAACAGCAAACCCTGACGCGTGTTTGTGTGTGTTTGTGAGAGAGAAAGAGAGA
GAGAGTGCATGCATGTTTGCATTTTAAAAGGAGAGGAGAAAGAGAGAGACAGAGAGAGCGAGAGAGAGAGAGAGAGTAAG
AGTGAGCAAGAAAGAGAGAGAGATGGATGAGCTGTGAGACAGGCGCTGTTCCAGAGACAGAGAGAGAGAGAGAGAGATGA
GTGAGAGAGAGAGAGAGAGAGAGACAGAAAGAGAGAGAGAGAGAGAGAGGATGAAAGAGAGAGAGAGAGAGAGAGAGA
AAAAGAGAGAGAGAGAGAGAGAGAGAGGAGAGAGAGGGAGAGAGAGAGAGAGAGAGTGAGAGGGAGAGAGAGAGAGAG
AGAGGGAGAGGGAGAGAGAGAGAGAGAGAGAGGTAGAGATAGAGAGAGAGAGAGAGAGAGTGAAGGAGAGAGAGAGAG
AGAGAGACATTTAAAACTGTGATCTCAGATGGAGAGAGAGAGAGAGAGAGAGCGGGGGAGAGAGAGAGAGAGAGAGAGA
AGGGGAGAGAGAGAGAGAGAGAGAGGAGAGGAGAGAGAGAGAGAGAGAGAGAGAGAGAGAGAGAGAGAGAGAGAGAGT
AGAGAGAGAGAGAGAGAGAGAGAGCATTGAGAGAGAGAGAGAGAGAGAGAGAGAGTCAGTCAGAGTGAGAGAATGAACA
AGCTGTGTGTCCATCACTTAAACTGTTACTGTGGAAATTCTGCTAGCTGTTACACCTGCACTGCTGTTTGTCTACAGGACTTCG
CCCTGTCCCCAAGGACAGGTTGAATACAGCGAGATATATTATACACAGCGAGCACGACAGAGAGAGAGAATTGAAATGAGC
AATATTCAGACAGATATAGCGGGACAGAAGGGGATGAGTAAACTAGGCAGCGCTCTCACCTTGTCTGCTCTGGAGGGAAAA

AGCACGATTTTTGACTCCCATAAGGTTTGGTACTGCGGTGAGAGAGAGAGGGAGAGAGGAGGTTTTGGAGTGAAAGAGAGA
GAGCGAGAGAGAGAGGAGGGGGTTAGCGGCCGTTCTCCCGCTCAGAGAGAGGGAGAGAGAGAGAGAGAGAGTGAGAGGG
AGAGAGAGAGAGAGAGAGGGAGAGGGAGAGAGAGAGAGAGAGAGAGGTAGAGATAGAGAGAGAGAGAGAGAGAGTGAA
GGAGAGAGAGAGAGAGAGAGAGAGCAGGGAGAGAGAGAGAGAGAGAGAGACATTTAAAACTGTGATCTCAGATGGAGAGAG
AGAGAGAGAGAGAGCGGGGGAGAGAGAGAGAGAGAGAGAGAGAAGGGGAGAGAGAGAGAGAGAGAGAGGAGAGGAGAGAG
AGAGAGAGAGAGAGAGAGAGAGAGAGAGAGAGAGAGAGAGAGAGTAGAGAGAGAGAGAGAGAGAGAGAGCATTGAGAGAGAG
AGAGAGAGAGAGAGAGAGAGATGACTTCGCCCTGTCCCCAAGGACAGGTTGAATACAGCGAGATATATTATACACAGCGAGCA
CGACAGAGAGAGAGAGATGAGGAGAGCACACACACACACACGCTAGGCAGCGCTCTCACCTTGTCTGCTCTGGAGGGAAAAAG
CACGATTTTTGACTCCCATAAGGTTTGGTACTGCGGTGAGAGAGAGAGGGAGAGAGGAGGTTTTGGAGTGAAAGAGAGAGA
GCGAGAGAGAGAGGAGGGGGTTAGCGGCCGTTCTCCCGCTCAGAGAGAGGGAGAGAGAGAGAGAGAGAGTGAGAGGGAG
AGAGAGAGAGAGAGAGAGGGAGAGGGAGAGAGAGAGAGAGAGAGAGGTAGAGATAGAGAGAGAGAGAGAGAGAGTGAAGG
AGAGAGAGAGAGAGAGAGAGAGCAGGGAGAGAGAGAGAGAGAGAGAGACATTTAAAACTGTGATCTCAGATGGAGAGAGAG
AGAGAGAGAGAGCGGGGGAGAGAGAGAGAGAGAGAGAGAGAAGGGGAGAGAGAGAGAGAGAGAGAGGAGAGGAGAGAGAG
AGAGAGAGAGAGAGAGAGAGAGAGAGAGAGAGAGAGAGAGAGTAGAGAGAGAGAGAGAGAGAGAGAGCATTGAGAGAGAGAG
AGAGAGAGAGAGAGAGTCAGTCAGAGTGAGAGAATGAACAAGCTGTGTGTCCATCACTTAAACTGTCAATGTTACAAAGGA
GCCCTCCTTGCTGTCTCTGTCAGGTCTTCAAAAGCACAGTACAAGTTTGGAGAAGAGGTTGACGGTGAGTTCAGCAAAGGAG
GAATAAAGGGAGGCCATCTGGAACAAATTAAAGGAGGCACAGAGGAGCAACAGATCAGGGGAAGACAGGCAGGTTGGGGG
AGGTGCACTGCTCTCCCTTAGGATCAGCAATCACCTCAGTCTCCGTGCGGCTTTCACAGCCTTGCTGCTCTTGGTGTCGTCGCT
GCTGCCCAAAGACTCCGCCACGGGAACGCTGCATGGAGGGGGTGCGGGGAGATGAGGCCACGGAGCAGCGGGAGCGTGCT
GAACCGCTTTCCGATGAAGAGCGAGTCATGATCCAGGACACCTGGGGGCGTGTCTATGAGAACTGCGAGGACGTAGGCGTCT
CTGTCCTCATCAGGTACTTCACTTGTTTTCTTTTAGCAACATTCTTTCTCTGTTCCACTGGGAGCTGAAAATGCACGTGGGTGT
CATAACGTGATGGTTTGACGGTGAGTACGCTGGCATGACTCCACACTAGACTGCCACGTTAAAAGCACAGTTACATCCATAT
TTGAAGACTCTGAAATGACAGTAGAAGATAGAAAATTTGTGCAGACAAAGAGCTGAAGGTAAAGCCGGCTTAGTATGCAGA
CTGTACTTGGCAGGAAAACATTACTTGCTTCCCATGCCTTCAGTTGACTCTTCTGCCTTCATTGTGATTTGATTTAATATTATA
AGCATACCTAAGAACCCAAATATAGCTGTTAGGAAAAAATTAAACCAGCACTGCTATGTTGCAAGCCTGCATACCTGTGAAC
TGTATAGGTGATTTCTTTGCAGAGCAAAATCTTTTGTTTTTTATTTCCACACATTGCCAAGATGGAATGTGTGCTGTAAAATTA
ATTACATTTAGGTTCTCAGAGAAGTTTAATGCACTGTGTACAAAAATGGATTCACCATAATTGAAAAATAAAAAGAAAAATA
ATGCAGATTGCAGGTTGAAGTATAAAGCCTATGCTTCAGCATGCACAAAAACGTTAGTAATAGCTGGCCGAAGCTGTAGCTG
TAGACGTCGTGTTAAGCCAGCTATGCATTTGCTGCAGACGCAGCAGGATGTCTCGCTCTTCGCACGTAAAGCGAGGTGTTGT
GCGTGGTCGCTCCTGTGCTCTAAATGCTGTGGCACGCGGCAGAACACTAGCGCGTGCTGTACACCGCGGAACCGTTCAGGCA
AACCGGGATTTTCGAAGCTAGGTACGCACACCGCTCTTTTTGAAGTGTGACCAGTGAAGTAAAACAACAGCACGTCTGCGGA
CCATTAGTGAACAGGAATGCGTCTGTGTTTCTGTATTGCTAATACCTGGACACTTATTTACCCGTGAGGAGAAGATGAAAGC
AAGGAGGAAATGCTAGTAACGGCTGTTGACGTAAAACCCTCTTTCGGAGTTCTTCAGTCGATGAGGTCTGAAGTGAGACTCA
GAAGATCGTCATTTATATTTTCCGGGTAATAAGTGGAGGGCCAGACTAATTTCAAATTGATTAAATTTACACATAAAATAATT
GCTACTTTGTCTTCATAAGAATTGAGCACGTGCTTTTCTTACATTTCTTATGTAAGTTATATCTACAGGCAGCCACTTATTAAA
TATTGTACAGCATGTACAATATTTTAAGTAGCATTTTAAAGCAAAATTAAAAGTCACTGATGTACAAGTCACCATTCAAAAAT
GTTTCCATCTTCCCCAAAAGCTGCAGATTAAGATTTTGAAGTGCGTGATGTCTTAACATTTGGGGTTAAAAAGTCACTCTGTC
TTTGAAAAGTTCTGAGGAAACACTGTGTTTGTACAAGTGGGCGTCTGAAGGCACCTTTCGTAGCACTTACATGCATGTCTGCA
TTTAACAGTGCATCATTCACTAGGCCTTATCTGAGAGAAACACATCTTTGACTCATTCTGTTTATCTAGACAATGTATGACTA
GTGCGAGTCACAGTTTTCTGCCCCCAAAAGATGATCAACAGATCTCAACTGAGTTACCAGTGAAAATGTTCAATACACCACC
AAGTAACAGTACTGATGGTATCTGTTAATGTAAAAGGAAGGAACAGTACTATTAATGAGAATAGAAATTAATAAAGCTCAGT
AAAATGTTGATAAAACATTTTGATAAAAATATATTGATTAGAAAGTACCAATAAAAAAAAAAATTAGTGTGGTCTCTGAATAC
ATAGCCACATGGTCATCTCATTCAGCAGGGGTCAACAACCAATTTAGTATTAAATATTGCTCTGTGTAAATCTAGAAACAAA
ATAAACATTTATCTTTTAGTGTTTGCTTTTCTGTGTGTGGGTCTAGGTACAACTGAAGGGACTTTAACTACAGATACAGTAGG
TGTTTGGAATTGTGACAGCAGACTGAGCTAGCCTGCTCTCCTGTATTTCACAAAGGGGATGCGTGAAACTGCACAGTGCCTG
CTATGATGAGACTTGATTTTCCTGACACTGATTGTCTGCCCAGTCGGCTTTTTTGTAGCGCTACAAGTTCCCAGTTGTCATTTT
ACAGAGTATCAGTGGGATCCTGGCCAGATATATGAGAAAAGGATTATTGCTTAGTTTATCAATGTGTTTTATTTATGAGCAGG
CTCAGATTTATAAAAAAAAAATGTTAAGCACCTTGTTGCACCACCCCCTTGTGTTTTTATGACCTTTAACCTTTAACCTTTGAAC
TTTGACCTGCACTTCCATTTCCTGTGTGGCCTAAGTAGCATTGCCCATGATGTATTTATAATATCTGCAGGCTTCATGCCATTG
CCCACAGAGCCCCTGAGCCTGGAGACCTTGGAAGAGCTCCAGCTGGGAGAAGGCCAGTCAGCCTCATTGTAACTCATACCTC
TCTCGGGTAAAACATACACAAGCCCTCCTTTGCACAGAGAATAAGATCATGTCTGCCTTGTGTAGAAGAGATGTGTGAACTC
CCACTTTGCGCTCAGATCCCTGGGGCAGAGGAAGGTGTCCTCACGCTCATTGAAAGAACCCCACGCACCCTCTTCCCCTACAT
TACACAACTCACGGGTCTGCCTTGGGGGAATGTCCACATCCATCAAATGTGTTTGTCTTAGGGAGAAACAGAGGAAATGTTG
AAGACAGGATGTTATAAGCAGGTGGATAGATGGTTACGGCCAACATCGCTGCCGGCTTCTTGGACCAGCCATGCAAGTGTCT
GGTTCTTAGGAAGATAGTAAGACAGTGCTGGAGCATTAAACGAAATTAAGTTTGCCTCACGACAAGTCGCAATAATGTGTCA
GAGAAGATACACAAGGACATATTTCATCTCCATTTAAATTATACTCACATTCTACTGAACAAGGAGAGAATTCAGTATTTATA
AAACATCTTATTTCAGCTTGCCTCTTAAAATCTTGATGGCCACCCCACGAGACTGAGGCAAGGGTGCTGAGGGCCATATCAA
CAAGAAAAAAGAGTGCATATACCAATTACTAGCCATGCTTACTCTATTCGCACTATAATTAACCAAAGGAGGATTAACAAGT
TTCCAATTGCAGTGCGCTAATGAAGTTATTTATATGCAGCGAGGGGTCGTGTCATTTTGTTGAGCGTGCCCTCACTCATTTTTG
TTCTCCAGTTTCCCTTGATGGTTCCTCTGATCTTGAATAAGATTCATGCAAAGTTAAATCAAAGAGCCTTGGGAAGCCAACAG
CTGTTGCCTGCTCTTTTGGATTTAACAACATGCGAGGTTTGCTCGGTTATAAATTAAACTAAACTTGGGAGAGATGAGCGTAA
CCTCCCAGAAATAAGATGAATTGTTTTCCAAGTCCTGCGGTGAGTTCACACAGCTTTGTGGATCGTATCCGTTTAGAAACGTC
AGGAAGTATCTTCCCACTGAAGGATGGGGCTCTTCACAGACATTCATACAAACACAGAAACTTGGCTCTTGGGTTATTGATTT
TACGCAGGCTGAGTGGGACATCTGGAGCTTATGGCCATTCAGGGTTCTGCCTGGAACCACTCCAGTCCCTGTGGGAGCCTGT
CACGGGATGTCTATCTTGAGGTTCTGCCTGAGGCCAAATAAGCAAATGAATAAATAAAAACCCTCTGTCAGCCACGGCTGTG
TGGAGTTGCACTGAGATGCATTAAATTGGGTCAGCGGCCGAGGAGGATGTAATGAAGGGTACAACTTGACAAAATGGAGGG
AGAAATAGTACTGCCGGGGTGGGTCCGAAGCACATAGATTAACAGCAGCTGTGTTTTTTTTTTTAAGGTGTAACACTACGAC
CAAAGGGCTCACACTTTCAGAAGGACTTAAAAAGACGGAGACATTTTCAGAGGTGGGGTCGACAGTTCCACTCACCTTCCGT
TCACCCATGGAGATGCAAAAATTGAGATGAGGGACCCATTTTATGGTGGTAATCAATGTGGGGGAATTACTGAAGAGGGTGT
CAGAGAACCATATGTGCCACTGCTGGGCATCAAGCCAGGGACAAAAAGCAGCAGTTACCCCAAAGCTAGTGTAATGGTCTC

CTTCTTATCTGCTCTGCTTCTCAGCCAACAGGCAGCCATGAGCACTGTCTGACACCAATGGAGAATGAATTTTGAGATCAAAA
GAACCAAGTGAACATGTCATGTACTGTCCCACATGTTCAGACACCGTGCCCCAACAAGCTCAGATACCTGTCAAGTGCTTCA
GGGCGTTCACCAGTTGCTTTCCATCAAATTCAGAGCCACATTTTTAACAAGCTCTGGCACCGTAAAAAATAAAGGAGAATGT
GTTCTATTCATATGGAAAACAGTGGCCAGTGAAGCAATTTGGATGCTCGAAATTGTTACTTTATGCCTTGCAGTCATGAACCT
GAAAAGAAGAAATTAACGTTTATTGCAAGCAGAAGCTGGCCCACTCTTTCTCTGCATATAAAACTACACCAAAAGAACAAGA
AGTAAACAGACAATTAATATTATAGAACGGGGCGAGCATCAGAGCTGCATAAGTATTCAAACCTTTTGCAATTGAAATGCTA
AATTAATTTGAGCTCATAAAATTACCATAATGAGCCATGCAATTATAGTTCATCATACATTAATTGTGTTTCTTGTGAATACA
GGATAGAAACTGTCGCTGAAGTTGATTGGGGAGGAAGTAAATTACATCCGAGAAACTTTCTAAGCAAGTCAGATCTGAAGC
AGACTGAAAGAGAAGGACGTAAACAATATTTGTATCCATATTTGTATGTCTCTGCAGATGGAATAGTCCATCTTCAAGAAAC
ACAGTGCACTGTACAAAGCAGAAAATGGAAGACAAATTGTAGTAGTGCTGTACTGCAGAAAGTGACTAGAGACTTGCCTAT
GCCGCCTGCCTATATAGATATGCTGCTGTATGCAGGTACCGTGTCCTCCTGGCAGCACATTTGAACATTGATGTGTTCAAATA
AACTGCCGATGTTACGAGTGAACTACCAAGACTAGGCAAGCGGTGTTTATCTCATAACCCATGGCTTCTCCTGCAGGTTCTTT
GTGAACTTCCCGTCGGCCAAGCAGTACTTCAGCCAGTTCCAGGACATGGAGGATGCCGAGGAGATGGAGAAGAGCGTGCAG
CTGCGCAAGCATGCCCAGCGGGTCATGAATGCCCTCAACTCCGTGGTGGAGAACCTCCAGGACCCTGACAAGGTCTCCTCCA
TCCTGGACCTGGTGGGAAAGGCGCACGCCATTAAGCACAAAGTTGAGCCCATGTACTTCAAGGTAAGGGTGTTTGGTGGCAC
ACAGAAACCGCTTGGGTCGTGGCAGCTCCTACACAGACACCTCAGTGCTAAAGCAATATGAGCAGTCAGTTTAGGACTGAGC
ATGCAAAAATTTTACATGTCAAGTGGGAGAGGTTGTAGCTGCCAAGTGACCAATTTAACAAATCAAGTCAAAGTGATCATTT
GTAAACATCACTTAGCCAGTGGATGTTTTGTTCAGCTCACGATACCTTTTAAGCTGTTAAAATGCACCTGCGTTGTCATTAGTT
TGACTGACTTGCCACCACTGTTGTCATTATAATCCTCATCAGGGAGTGACACAACAGTACTGCAGGTAGCATTGTCACCTATT
GAGCATGGGGTAGAAATCTTGCCCTAATTTCTATCTGTGAGCAGTTTGCATGAATTAACTGCTGGTGCTTTACTTTCCTTTAAC
GATCCATGCTGTGTATGCACTCTGACAATTTCCATTTGCTGTTAGCACATGACTTAAGACCCAAGTTAGTGTTTTTCAACAGA
ACCTTATGGTTGTGACCCATCACCATGACCCTGAATTGCTGTTTACTGAACATGTGTAGACAGATAGCCATCAGGCTTGTTCA
ATAATTAACTGCTGAGTGCAAAAGTTCTGCCACTCACACTCTTAGCATCTCGCAGCCATTCAGACCAGTGTGCAGTGACTTGT
CCTTTTCCTCTGTCCCAGATCCTGAGTGGGGTGATTCTGGAGGTTCTGGCGGAGGACTACGCTGAGTGCTTCACGCCAGACGT
GCAGAGGGCCTGGGCTAAGCTCATGGCTCTGGTGTACTGGCACGTCACGGGGGCCTACACAGAGGTAGGCTGGGTGCAGCT
GTCCAGCTCTGCGGTGTGACCAGGGCAGCCAGGCGCAGCTGTGCTTCCACATGGGCGACCTGGACCAGCACCACCGCATTCT
TTTCAGCCAGAGGGAGCGGGAGTCTTACTGGAGCCCCCCTCTGCAAGGGGGCCAGCCTGGGTTAGGAGCGCAGAGGGTATG
GTGGGGCACAGGGCAGAGTTTGAGATCAGAGTCTTAAGAAAAATAAAATCTCTTACTGCTGTTTATTCCTCTTCACAAGTAC
ACAGGGGAGCTTCCTTGTCTGAATCATGCCGTTATCTTGCGTAATCGTGTCCTCTGAGATGTTGTCTGTGTTGTCTGTGGCTCG
TTAAGTGTACGTGTAAGTACGGCGTCACTCACGGCCTGTCCTGATTCTCCTGAAGCTGTTTTTCTGTGACATGGTTTGGACCGT
TTGGTGATGGGGGTGGGCAGGATGTCTTAGCCCTAATGGTTTTCCCTTTTTTTTA

>Ngb_Chitala_NODE_893216_length_12521_cov_10.589090

AACATTATAATTGTTGAAAAAACATAGTGTAGGCAATTAATTTTGTCTCCTTTATTTGTTTATTCTGGTAAAATTAGGTCATGC
AAACAGTGCTCTAACACTTTGTGGTCACATTCATTTCATCGGCAGCCATCAGAGCTATTTTTTTGATTTCCAAAACCAATTAC
AGTCGCTAACTTAAAAAACATCTATAAAAGCATGCAGACAGAGCTCATATTCATTATAGCTGTTTATATTGCACTTCAGTTCC
AGCAAATGTTACCCTTCTATTCTTGCATTCCCAAGTCATGTTTCCAGGTCTCTGGTGAGCTGTTCCTGAACTCCATTGCCTCTG
TGTAATGATTGTCAGAGGGACACACCACTTCCCATCTGCCATTTGATTTCCTGTCTCTTCTCTCGCTGTTTTTCTCCGTGGAGG
GGGGAAACAGAGGTCGGCTGGGAACCGGGGGCATATCTGAAAGCAGCAACCTGCAGAGGGCACTGTGGACTGCCACAAAC
ACAGAGGAGAGTGAGCGGGCAGGCGGGCGAGCAAGAGGCCCCCTCCTTCAGCCACGGGAACAAAGCCGCAGAGCCGGCAG
AGAGCACCAGGGAAAGAGACGGCAGCCCCAGTGACGGATGTGCCCTGGGGCCACTGCCTCACCTCCATCACTGTGTTGTTAA
GTTGGAGTGCGTGAACGCCGGTCCTCTGCACATACCTGGACCACTCTGAAGGTATGCCTTGTTCTTCTCTGTGTGGGAGCGGG
TGGCTGTGGGTTTCAGTCTCAACAGCTGCAATGATTGAAGGATCGGTCCTCCTTCTTAAGAGGCAATGACTCCGTACCCTCTC
ATCTGCTGCTCACACAGTCTTTGACTTTGTTTTTTAGGAGTCACCCCAACGGGAGTCTCCCACGCCCCTTTATGTGTAATGGGT
CTGGCTGACGTGTGAGGGTCCACCGTGTAGATTCCGCGGCGCGCCAGCGTGATTCGAGGCACGGCTGCATCCAGACAGCACT
GTCACATCACTGTCACCATGGAGAAGTTGTCGGGGGAAGACAAGGAGTTGATCCGAGACAGCTGGCAGAGCCTGGGAAAAA
ACAAGGTGCCTCATGGCGTCGTCATGTTCACCAGGTAAGGTCTGCAGCAGGTGTTTGCTGCCACTCAGGTGAGCCAGGGCAT
AGTGACAGATAAAGGGGTACTTTTCTGTAAAGGGGGCCACTACCCGGGTACCAGCAGTCTAAATTTTCTATTAAATTTTTTAA
CACTAATATTGTCCACCTTTGACTTTCCTTTCACTTGTGTAGGTCTGCTGGTGTCTTCAGTCATTTCAACACATTTTAGAGCAT
GAAATGAAACTTATGTGTGCTTGTCTGGCAATGTTTATTTCTGAATGTTTGATATCAGTCATAAAGCTGTTTAGGCCACATGT
CTTTACTCCTGTTTGTTGTGGAAGTTTAGTAAAGGCATTTGAGACATCATTGTTGCAGGATTGCATGTGTGGTGACAGCTGTT
GCTCAATGTCAAGACAGAATCTCATGGGGGTCATTAGGAGGCCAATGAGCTTCTCACAGCAAGTGACACACAGCTGACAGTC
GGTCCCTCAACCTCCCATCACTGAACCTGCCTCTATTTGTTGCTGGGAGACAACAAGGTCTTTTTAAACCTCTGTTTAAAAAA
AAAATGTGTTTTTTTGCCATGAGTAGATCTGTATCCTTGTGTCGTGCGTGGCAGATTGTTCGAGCTGGACCCTGGGCTTCTGA
ACTTGTTCAGCTACAACATAAAGCACGAATCCCCACAGGACTGCCTTTCTAGCCCTGAGTTTATAGAGCACGTCACCAAGGTT
AGCCAGCCTTCAAAATTATCTCACTCCTTTCAACAGGGGAAAAAATACAGTGAAATTCTTGTAATGAAAGCAGTACATATTTT
GTCAGAGGAAACTTTCCTGCATTTAAATTCTGGCAGTAGATTATAGTGCTGTAATTTTAAAAATTTTAGTTGTACAGATCAGA
GTCAGTAGCCTTTCTGACCATATTTTTTCACATGCTAACAAGTGGCCAGTTCCCAATTGTCCTCAGAGTAAACATGTACTCAT
GGTTTAACTTTCCTTCGGATTAAGCTCTGAAGTGTTATGTACCTGTGCGTGGCTCTTCAAGGTGATGCTGGTGATCGATGCAG
CTGTCACTAACCTGGATGACCTCCCATCTTTGCACGACTATTTGCTTAACTTGGGGAAGAAGCACCAAGCGGTCGGAGTGAA
GACCCAGTCGTTTGCTGTGAGTCCGCTGTGGCTCTGCTCGTCTTTTTCTTCACAGAGTCACCAGACTGGTCATATAAACATCTT
TACATAGTTTTCTGAAGCTAAATAATGTCTTTTATGCTCTTAGGGGCTAAGTGGGATCAGCACCTTTAGCCTGTACAGGGTAA
TCAGGACAGTCTTGCTGTGGCTCTGTGGGCCATATATGAGTATGTACTCTATGCATGTGTGTCTGGGTGCCCTGCAGATGGTG
GGAGAGTCTCTGCTCTACATGTTGCAACACAGCCTGGGCCCAGCCTACACCGCCGCCCTACGCCAGGCCTGGCTCAACATGT
ACAGCATCGTGGTGGCATCTATGAGCAGTGGCTGGACCCAGAACGGGGAGCATGGCAGTGAATAAGCCCACCCCAACCTGG
CTGCCCCAGGGGTGCATACGGCACCTGACCCTCTTGCAGGTGTCAGTCAGCCATAAGGCTGCTTGAGGTAGGTTACTGGGAA
GCTTTTTACACGTTTCTTTGGAGCGCAGAGGGGTAAGATCACACAGCAGGGGTGGGAGGCATCGGGTCGGACGTACAATGTG

GCAGTAGTAACAGCTTGAGTGATTTCGACACACAGCTAGCACATAGCGTTCTTGCAACATTGCCGATATGCTCAAGTGAGAT
AGCAACAGCATAGTGCAGCCACGGTTCTGCAGGAATGGTGTGCAAATGTTAGGTGTTAGATGAGAGCAGGCACATGCTTTTT
CTGCAAAATGTGTTCAACCATCATTGCACAGGTTTTAGTTACAATTCTCTTTGTATATATGTACAACGGCTTCAAATACCTTCT
CGGTGCTTGTGTTGTGGTGTTTTCATGCAAGTCACGATGCTGCTGTGATTGGCAGTAGGGCAGCAGTTAATTGTTTTTAAAAT
TGTGGCATAAAAACCGTCAGACTTTTCTTTTCTTCAACATGCAATGATATCATCAACCCCCTTCAAGCCTTGTGGAAAACTTA
GCCATGTGAAAGATTTTATCAAAAGTTCAACTGGTTTTAAAATGCACATATGTATATGTAAAAATATAGCACATTGTCACCAT
GTTTGCTTTACCTTGAAACGGTTGACCTTTTCTCTTTTATCTTATGTGTAGTGGTATTTATTGTCCTTTTGAAATGTAACTTGTT
ATTAATGTGTTTCACTGCTGTGTACAATCCACGGACTTTATACTCTTAATTACAATGTTGTCTGTAAGTACAGTGAGCCAGTGT
ATTTTTTTTATTTTAGTGTCTGCTAATTTATTGTTAGTCTTTGGTTACTGATATTGTGCTGTAATCACTGGTGGAAACGTTAGCC
ATTTCAAGTACTGAAGTAGCTGTATTACATGAGAATGAACCTTGAAAACTGTGAAATGATTAAATTAATGTCTTTTTGCACTT
GGGTATTACTGAACAAAAAATAATTTATTGAGAAAATATATGTACACAAAGAAATGGAAAAATATAACTATAATATAGTTCA
GTTCTTAAAGTGTGACTGTTTTATCATCAGGTATGTTAGGGCACTGTTTTGTGCATCTGTGACTGTTATAATACCCACAGAGA
GCCTTATTTTAAAGGTAAACATGTGACACGTGACAGTTTTCCTTCTGACCAGAGGTTATGGAATTGTAAATTAAATCACTTTA
TCATGCACCTGTTTAGTTACCATTAAGTTGACTGTTATTTCAACCAATATGAAAACACTGTTATCACTTTAGGAGGACGTTGA
TGTAATACTGTTCTCCGCTGACCGCATACTGGCCTGCTTAAGGTATTTCAGGATTTTAATTAAGTGTAAACCTTTAGCAATGA
AAGCCCGACCATTTGGAGGCCTGGATTGAAATTAAACACCACTCGATAAACTGACAACCCCATTTCAGCGGACTGACTTTGG
TTTGGCCAGTTAATTAAAGGGACCGGTTATGTTAATCCATTCCATCAGAGGGCTTGCCATTGCTTATACTTGGGATAAATGCC
AGCCTCTACTAATTCTTAATGCTGTCTGTGTTGGTGTGTCTGTGTTAAGAGGATCGAAATGAACAATGTTTAATATGTTTCAG
ATTTGAGAATGACTCATGCCTTGAACCTGTAAACACTAGTAATTATGCCAAGTAGCATTCATACAATAGAAGACTGTTTTTGA
AAATAGCTGCCTGTCTAGCACTATTAGACAAATTGCTTTAATAAAGTTGTTGTTCAGTAGACATGTACATTTTGCCTGTAACT
AATGTAGAACAGTGGCAATTTGCTGTCAGTGATTAATGAGTGACTGCAAAAGCATAGGAACCAATGAGATTGGGGCGTCCTC
CCCAGTATTTAAAACTGGTATATATTTATATATTCTGGTTACAGTTGTAAATCCCACAATACTAACCAATGTACCCTTCATTGC
CATACTCTCCTTTGCCCTTATAGCGGTGCATATTTTTTTGTGCATTAAATTCCTATGTTCCTTTGGTCACTAGAGGTAACTCAC
CATGTGAAAGCCATATCTCTTCATCTTGGTTTTAAAATTCCAGTAAAGCTTTTGAAAAGCAAAATGTCAGATTAAATCACTGC
AATATGCTTTCATTCACTGTAATCGCTGACATTGCAGCTGCATAATGAACATACTCATGCATTCAACAAGCCTTTGCATTTCA
GATTTATTCCTACTTGCATTTTAATGTCCAGAGGAAATGTTTTCTCAGACAGAGCTGTTAAAATCTCCCCAGTTTGACACAAA
ATTTGCACAAGCTTTTTAAGTAACCGAAAGCTTTCACATTAGCTATTGTCCATTCTTCTGCTATTGAATGTTGGTTGTAGAATT
ACTCTGCTACTGGTTTAGTCCTTGTGAACTGTTCAGTCTCTTTCAGATACTGTGTATGCAACGAAGCCAGCTTCATAAACTCTT
TATTTATTTTTTGCTTCTGTGTTATTTGATGTTTTGTGTTTTAATGTCTACCTCATTCTCACGGCTGATTTTGGACAGCCAAGGA
TTCAACTGAGCATCCGGTAACCAAAAACGGTTCTGAAGCTAAACACACAAGCACCCTCGTCAACCTTGCCGAGCTGCTCCAG
AAAGCAGAGACTTCACCTTTTTGTGTGACGTACTGTACACTCGACTGTTAAGGCTAAATAAAGGTTCTCTCATGTGTCTGTTT
GTCTGTCTTCATCTACCTCTCTGTTGCCTCTCCTTCATGCAGATTGCGCTTTTGATCAGATGCTGAATCGAACACACACAATAT
CCTCAACCTCTGCCACGTTTGCTAGGTTATGCAAGCATCCTCGTTGATTTCCCCCCCACAGGGTACAAAGATGAGATGGGGGG
TAATTCAAATCTGAGGAGTGGGTGGAACACTCCCCTCCCCTGTTGAAAACCACTCAGATGTCACATCATATTTTCAGGAGCAT
CTTGGCTAATGTGCTACTCTTTCCCTGTCAGGCCCTCTCTTAATGTGTAAAAATGGTGCTGACATCTAGCCGGTGACACCCAG
TGGTTTTGTCATTGCTGAGCGAGTGATAAAAGACACAAAGTCTCATTACTGTAACTAGCACTTTAAGCAGGGGGATGGATCT
GCAGGCAGCTGTTCCCTTAAACTTTAAATTACGTTTAATAAAATGTCCACATTTTACATGCCGATCTCACAGGAGAGCAGTAC
AATAATCTGCTACATGAATTTTTAACCTGCTTGTTTTACAATTAATTCCCAAGAAGCACCCTGCAGTAACACTACAAGCTACT
TTAATATGCTGTTGTACAAATGGGTAAAAAAATGCTATGTAAAAACACGGAGCAGCCAGGCACTAAGATGGACACTGCAGA
ACTAAAGCGAGAGATGCATCACCAACAATTCTGTTTGGAATTTCCAAGAAAAATATGAAGATTTCCATGAATAGGAATGAAT
GTATGCCACATTGACTGATCTGGAGAGTATGGCCTTTGTAGTTACCATAGAAACTATATCATGTTCCTTCGCTCAGTGATCGG
ATAATTATTAAACATACATTTGAGAAATGCATAGGACACCATTTAATGCTTGGGTGACAGCAAATAAAGCCTNNNNNNNNNN
ACTTATAAATTACCATGCATTTCAAATATTCAGCTTGCAGTTTTTATGGACATAAAAAAATAAAAATCATGAATGTAAGTTAG
TTAGTCTATCTGAACATTGGTGCAGTACTGCAAATGACCAATTTCAGCATAATCTTTGTACTATGATTTTTGTACAAAATGTTT
GACAACTCAAGGAATTTTAGTTTTAAAGGTAATGAACCTAGAAATTAATTGAGGTTATGATAGTTTATCATTGGGTATATGAT
GACTGCTGGTGAGGATGACTCCTGTGACTGTTGGAGAAAGGTTCCAGTTGCCATGAATCAAGTCTGCTTGATGTCACAGGCT
GAGCTGAAAAGCTTGCTCACAAAGCGGCCAAAATAAATACAATTCTCGTGAACATCCCACCTTTCACAGCGCTGTCCAAAAA
AGCGTACATTACTGAACTGCACAACAGACGCAGTTTCACAGCCATATATCGGCAACAAACCCAGATATACATCGTGCCCGTC
GTCTGCAAGCAAGACCTTTTCTGATAGGTTGAAGAATTTACAGCTGCACTGTCGTCTGAACATACATTCATCTCATTTACTAG
TGTGTACTATAACTTAAAAATGGGCTTTTAAAAATGAGAGGTAATTTAAAGCATGAAGAAACCCAATTAATCTGCCTCAAAA
GAGCTAATCGCTTTGTTATGACACCTCGGATTTATTTCAGCAAATGGCTTTACAGCATCTAACAAATGCGCCGCTGTCTCCGA
ATCCATTAAACGGGCTCGAAAAACACCTCACTGGCCATTTCACTCTGGTTGCAAGTTACATCCTCACTATTTGTTTGTCAAAC
ATTTACTTCCCTGGGTGATTGTAGAAAAATACCACTAAGAAAGGTATTTATGTGTTGTTGTGAATCTGTGCAAGATACTCTTC
CATAAAAATGGGTAGAACAGGGTGGGGTGTGGACATCGAGTTGAAAGGTGTGGGTATTAGTGCCTCCCATTTTAGGCGTGGC
GACAAAAACACAGCAACGTTTCTTTCTACAGCCATCTGTTTGTTTATACAAGCTGCTTCTCCAATACCTGTCACAGGTGCGTA
CATGAATATTAGAGTACTTCTAATATCGTATTTGCTCAGGTTGGTATGTAGCACTACATAAATGACAACTTATAATGTACTCT
TGCTTCACAAAAACAACTGCAACACATTTTGACAACTTGCATCAACAGGTTGCGTTTGCCGATTTAGGGCACCCTTGCAGCAA
TGCAGGATGGAAGCGAATGCTTTTCTCAGACACTACGCAAGCAGTCTTGCACGCGAAAGCAAATAATGAAAATGGGAAACG
TAGAGAAAGCAGGTTTGCCAACGTAGGCTGGGAACATGCGCTGGATTCTGAGGGTGGAGGCCGGAATCTATTTCTGGGCGCC
TCCAGGTTCTTGACAACCATGCAATTTCAGCTCTGGGGGGACTAAAAGAAACGCTGTCTGAGCTAAAAGACCACAAATCAGT
CGCATTACGCTTCTGCAGCTTACAGCTTCTCCACAGATTTTTGGCCAATCTTACCTGCCTTCAGAGCTATAATTAAAATAGATT
ATTAGTAAATAGAGAGCAGACTAAATACCAGCCACATATCCACCCAATACTGCTTTTCCCTAATGGACATTCTATGTAAACA
ACCATTAAGGAAAACAAATAAAGCCAGTTCACAGCAATTAAAGAATCTTTACGCTGTGTGACTTGCTTCTGCCCTCCAGCAA
AATATTTAAGCGATACAACTTGCATTATTTCAGCACATCGAATCATATTTGCTCAGGTCGGTATGTAGTAAGGTGTATCTAAA
AAGTAAACGATGAACCCTTACAAGAGCAGGAGTCAGCTGAGGAGTGAGACAGCTCATCCACAGGAAGGTCTGAGGTTGCAT
GGAAACACCAGCAAGGCAACGTTTTGAATGTTAATTAATTACCCCCAGTTAGGGGAAGTAAATAAAAAAAGGAAAAAAAAA
TCTAAACAAAGACAATCTTCGTCATCATAGTAATTAGCCACTTATGTTCAAGCGCAGACCTCTACAAGCTGTCAGCAGTGAGT
CTATCATCTGAACGGAACCAATTCAGCTCACAACGAGACTGAGCAGCGTTTTACTTTTGATATTCTTCACATAACCAACGCTG
GCACAACGCCGGAACTAACCCTTGACATCTTAATTAATTCATGAAGACATAATAGAATGGCACTGTTTGTGATGTTTTAACCA
CTTTGCAAAAAATAGACATAACTAATATTTAGCTAAGCAACAACGTCTTTCTGAACAAGACGCGAGCCGTCAAGAAGCAATA

TAAAGCATTTTGGTAATTACATGCGCAGTCCACAGAACAATGGAGTATATCCTGACTGCGTCATATCTACACAAAAATAGGA
TGAACCTGTATTTGGAATCTAATTAGTCAGACCTGCAATATTCATTGTCAAGTAGTAATTATAACAATTTACTTTTGCTTTCAC
CAGAACCAAGTGTTTACATGCTGTGCTGTATTTAGTTTTGAAGCACTTCAACCAAACACATCACTGTTAAACTTGCCGACTGC
GGGAGGGGAAACAAGAGTGCGCGTGTGCTTTAGTGTGTGAGCAGGTATGTCTTAAACCTCAAAATTAAAGGGATAACTTTTT
GCAATCACCCTTTTCAGTAAATGTGTCTGAAATTGAATGCTGTAAGATGTGTATATGAACATGTCTTTTGAGATAAATCGGCA
CAGCCAGTAAGGAGTAATCCTTTAGTAGTACTACGTCAAGCTATCAATAGCTTCCATAAGCTGCTAAAATTCTGCTAATACCC
TTGGCAACAAGACATGAGGCATTGAGAAGAGAATGGGAGTGAATTTGACAAACAACCCAGCAAAGGAAATCCTCCTCTTGC
TCTGTATGATATTAATTACAGCTCCTCCTGTCCACTCAGGCAAGTTGGTCTGCATGCACGGCAGAGAGCTTGAACATTCGTTT
CCCTGAAGCACCTCGTTAACAGTGGCATAGAGCAGTCAGGAAAGTGGAGCCCTTAAGTGACCTGCTGTCAGCACAGGCGGTC
TGTGGGATAGGCAGGCACCTCAGAACAGAAACAAGGCCATCAGGATCTCCACACACACGCACGCACGCACGCNNNNNNNNN
NNNNNNNNNNNNNNNNNNNNNNNNNNNNNNNNNNNNNNNNNNNNNNNNNNNNNNNNNNNNNNNNNNNNNTTGAATGACATTAGCA
ATAGGCAGCAAGAGGTGCAGATACAGGATACAAAATCTGTATTGTAGAAAATGTCACTTGTTAGAAAACAAATTACATACTG
CTTATCAGTTATTAATGAAATTATTTGTATCACAGCTCACAGGAACCTGAGCGCACTGTGGAATGTCACGTTAAGTCCTAAAC
CAACAGTGCTTTTAAATGTGAATCTCTTCTGTGACACTTGACCTAACATCTTACAATCTTCACTTTGCAAGCCACTGTCCTGTC
ACAATTCATTTGACACGCCTATTCCAGGATGAAGTCTGATGGTGACAGGAGGTAGAGCACGTGTGTATATGCACGAAAATAC
ATAGGTGCATCTGACATTTTGTTTTGTACCCTTACTTAAAACTCAGGCTTTTCTGAAGCTAAGATGTGGTCTGAAGGTCTAATC
TACATGCTGATTGACCTAACAGACCCCGGCCCATCTTGACACCACTGCTCTACATAATACAACGATTGTCTTCTAAAAACAGC
TTGTCCCATCTTACAGGCGACCGACAGCACCATGTGCCAATAAACAGGCAGCACACCGCAGTAAACCGACTTCACTGAGCTA
ATCAGCATTGTGGAAAAAAGCATTGTTCCACTAAGAGAATATACTGGATTGACCACTACCATGCTATGAAACTGACTGCAAA
AGAACAACACAACATCCTACAGCTTTCAAAAAGTTGCTCCCCGATACATCTAGCAATCCCTCCACGTTCCAGGAAACACACT
GTACACCATGACAGCACCACAGCCAGGTTGCGTTCATACCAGAAACTCTGACCCCATGGACTCCACTGTTCAGTCTCAACTTC
TGTCATTTCTTGAGCTCCTTTTGCTTTTCTGCTGACAGGAGTGGATCTCAGTTAGGTCACTAGGTCCCACACACCATCTGTGAC
GAGGTACAAACTATGCATTCTTAAAGCTGCCTTTCTGCACCAACACCAAGCTGACCAAAGCCGGTATGTTACTCCACACGAC
CCCTGCGAGCCTCCATAACAGACAAGTTTGTACCACAGGATTCACAGTGGCTGTATGCATTCTGGGGGAGCAAACTGACACC
CGACTCACCCTCACTCACGAAAATCCCAGTGCGATTTCCTCTGCTACAAAACATCTCCACAAACCATCAAATCTATTTGGTAT
GACAGTGTAGTTTCACGACTAAATAAAGTCTTCTCTCAGGAAAACCGCTGTGCGAGGGACATGCATTGTTATGACAGCCTTCT
CACACACACAGTAGCCTCACAAAGAATCTGGACATGTAAGCCATGCTTAGAAAGTTCTGAATGTTTCTGAATTATACAAAGT
GTCAAACCAAATAAGCATTTTTGCCACAAATAAACTCAAGCACATTTTTCACTCAAAGTATTTAACTAAAACATATTGGTTAG
GCTTTAAATTGTAAAACAAAGAATGTACTGTTCAAAAGACAAAATAGTATTCGGACGGTTCTCAAGCTTAGTGGAACATGTT
CATGGAAAATATGAAGATGAACGAAACCTGTGGTTACAACACGTGTGTATCCATAAAGACAACTGAGTCATAAAGTCACTAC
AGATTATTTTGGAGCAGTTGTTTAGTAATTCTAAAAATGGAAAAGAATAGTGAAGATAGTTCTAAGAAATTTGCTTGCACAA
AGTCAACTCTTCCACAGATCTGCAAGAAATAATTGACATTTTTATGCAGGGCTTACATGTTCAAATATTTTTTGGGGCCAGTG
TATGCAAACATTGACATATTTCAGTATATATTGGTTTGTAAATATTTACACAGTGAAAAATTATTTACTCAATGACCTGCTAC
TTACACCCTTGTGGTTGGAAGACAGAACTGTTACAGCAATAGCTTGTAAAAGACTGAGAATGCTGGACACACTCTAGCCATG
CACCCAAGTGATGCGACAAGTTTGGAACCAAATAAGCATCCTGTACTTATAACACTCCGGCCAAGTCTTAAAAACAACTTCA
GAGTAGGCTTTGCAAAATTATTACTGCAAGACAGAAAAACAAGATTAAGAAATTATCTTTTAACGTCCTTTTCTTCAAAGAA
AATGAACTTTTTGTGAATTATATCGGCAAATATCACAAGAACAAAATAATGAAAGAAATACACGAGAGGCAGAAAATAATA
AAAATGAACATTTACTTGAAAACAGAAAGGCTAAACGTGGTGTGATTATGTTTGTGTCACTGTAGGTCTTGCCAGCTGAGAT
GTTTTTAGACAGCTCTAGGAGTATGTAATTATGTTTAGGCTGGAGGACAGGCACCTTTCCACTCTGTGCACATAGAGTATCGG
TGGGTCAGTGACCAGCACAGGTCAAAACATGAGAGCAGAAGCCCAGGGCTTCAACATTTTTCAATAAATTTATTTATATATA
TCTCAACAAATGTATATTTCAAAACTCCATCTTTGTCAGGTAAGCCTCATTTGACACAGGTCTGAAATTTATATGCGATGAGC
CTCAAGAGGAAAAGCAGAAATTAAAGCTGCAATGAGTTTTCACTGACGAGGCCGCAGTCTAAACGAGGTGCCGTACACTCG
TGTGCTTCACGCTCCCAGTTAGACACGAGGCCGGTGTTAAACTCAGAAATGTGACAGCTGGAAGTGAAAGGTATTTTCTGAG
CCACTAAGGTGCTTCAGTGCAGAGCAGAGAGCAGCATTAGGCTCAAAGGGCAAGGCCTGGGCGCACTGAATGGTAAAGGAG
CTTCTCCACGCGTGACCTGAGGTGAGCAGCGGAACTATCATCTAACAGGACATGTGGCCGATACCGGAGGAGAATTATTCTC
CCCCTTTTGCACATGATGAGAATCTCTCGCTCTAGCCCGAAACTCAAGCTCCGCAATATTCGGAGGGCATGAAAGTGGTTTAC
AAGGGGTCTTCCCATCCCTTACAGCTCAGGGATAAGATGAGACACCGGGTGAAGGGGAAAGAACATTCACATCAGATTGTTG
TTGGCACAAAGTGATATATACTTCTGCATTAGTTTCACACTGATAAATAAATAAATAAATATTTTATATA

>GbX2a_Chitala_NODE_364716_length_6189_cov_8.437712

CACACACACACACACACAAATGGCAATTTAGGTTGTCCAATTTATGTCACCAGCATGTTTTTGGGGCTCTGTGAGGAAACCC
ATCCAAGCCTCACAGACAGTGCAAAAAGCCAGAATTGAACTCACAACCCTCGAGGCTGAGCCGCCACACCGCCCCCTGTTCT
CTTCCCATCATGGGATAATTCAAAGGTCCCTACAGCTACAGGGTTAACGTTTGTTCGTACAGCCCATAACAGTAAGTGACACT
CAACAGTAGTTCTTTTGTTGAGCATATTACACATCAACGTAATTATACAGTTACAGTTTATTTTCTTATATTGAGCAGTTAAAC
TGTTCTGGTCATTTCAAAAAACTTAAGTGGTTTAATTTCTCGAAAGAAACGCATGAAAGACAGACGTCTTACGGAGCCGTGTT
CTTTACATCCTCAAAGTAAGCGCTCACGGCTGGCGCATACGTGCAAGTCAAGCGGCTGTGAGCAGCGCATCTGTCTGTCCTG
AAGTACAGCTGTGCCATCAGCATCTTCTGCCGTTTTCGACACTAAGGAGCACAAACAGCTGCGGCTGTACCACTCGGACCAT
GGGCTGCGCGATATCAGGTCTAGGGTTAACGCCAAAGGCGGTGAGACAAGGGAAGGACAGCGAGCCGACAGCCGGGCGTGT
GAGCCTGGAGCAAATCGAACTGATCAGAGGCTCGTGGAAGGTCATCCAGGAAGATATCGCTAAAGTTGGGATTATAATGTTT
GTCAGGTAAGAGTTTATTCTACAGCAACGCTGCATGGGAAGTATTCTGCAAGGCAAGTGTAAAATTACCATAGGTTACTGAT
CAAAGGACGCTTATACATGTGCAAAACTCATGCGGACGTCTCTGGGTTGTTGCTTGTTAATTTGTCGTGGTGTCATACGTTTT
GAAATTAAATTCGAACGTGCAATATATGTTAATACCGTTTCGCAACCAGAGCCACAAATTGCTTTTACGTCGCATTAATATGT
TTATGCGAAATGCCTGTTTTTTTCGCGTTTGAAAGAGTTGTTCACATAGTATTTACAGATAACCGCAACGTTGCTTGAGAACA
GGCAGGTAACAGTGCATGGCTGGAAAAGAAGAAAGCAGCAATTGTTGTAACACTACAAAAAGTAGGCTGCATGACACACTT
ATCAAAACTGATCTCAGACCGCCAGAGAACAGATTTGAGGACGGTTGTGGATGGTTTTGATTTGGATGTCAACATCGGTTCC
ACGACAGATCAACAGCTCGTTCATTTGCCGCAAACTTTGCCCGTGCATCACTGAGACGTAAGAGAGAAAGTGCGTGTGCTTC
AACAGGACCTTCAGTTTCCACGCACGGCTGGGAAAATAACAGCCGACAGTCTTCAGTGTTTCGCTAATCCTAATACCCACGG

GCAAATACCAAAACAGTGCCAGGGTGCAAGATGGTCGTATGTTATCATACATGGTATTCTGATTGTTACATAAAATCACAAG
TTTACTAAGTTGATGTGTTAATTAAAAACTGATCTTATCATGAACTTGTCACGTGCACCTGGGCAACGCGTACAGTAGATAGA
TGCGATGGGATTGGGAAGAACTGGTGGACACACGTTTTTGGTGGTCACTCAAAAAGACCCTAAAGAGCAGTGCCTGACAGCC
AACACCCTGTATTTTGTGTATACAGTACCCTATATATAAAATGTACTTTTTCGTGGTTCAAATGTTACCGAGAAGATCTTATA
AAATCTGTTATGTGTTGTACTCACCGAAAGCCATGCCCGATTTTTAATTTCGAGTCCTTTCGTTTGCTGGTAATATTTACCTCT
CACCAACAGAGGGCAGAAAACATAGAATTAACCTTTCCTGCTTTCCACGAAGAACATGCGTTCGTTGCATCACTCTGCATTGT
ACAATTAATGTATTTCAGTTATTATTATTTTTTAATCCGACACAGTTTTAATGATAACAGGGCAACGGTTAAAGATATAAAAA
GTTCTTAAAGGTGTGCGAGGCAATGACCTACAACATAATATCCATATTAAAATCCCAGGCAAGGGAAGGAATCATGTTTTAT
GGCCAAGTATGTATTGTCACATACAAGAAATTTGTCCTCTGCATTTGCAAGGAATTCTTGTCCTCTGCATTTAACCCATCGCC
CTATCCTCTGCATTTAACCCAGCAGTGAGAGCAAAGTACATCAGTCACATACAAGGAATTTGTCCTCTGCATACAAGGAATTT
GTTCTCTGCATTGCAAGGAATTCTTGTCCTCTGCATTTAACCCATCGCCCTTGTCCTCTGCATTTAACCGTCCCCTAGGGTGAG
CAGTCATATATGGCACTTGGGAAGCTAGTGCTGGGTGTCTTGCTCAAGGACCCAGCGCTGGGTGTCTTGCTCAAGGATTAGT
GCTGGGTGTCTTGCTCACTGGTGGCTGGCCAAGGACACAGGCTGGGATTCAAACCAGGAGACTTCAGGGTGGGATTCAAACC
AGGAGACTTCGCATCCCAGCCTGATGTTGTACCCGTTGAGCCTTAAGCCTGATGTTCTACCCATTGAGCAACCTGCCTGATGT
TCTACACATTGAGCCACCAGTGAACCAGGGACCAGGAGGCTTCTGCCTGGGAGACTTCTGCATCCCAGCCTGTTGCTCAGAG
ACTTCTGCATTCCAGCCTGATGAAAACGTCGACATCCCAGCCTGATGTTCTACGCACTTCTACATCCCAGCCTGATGTTCTAG
CTTCTGTATCCCAGCCTGATGTTCTACCCAGGGTGGGATTCAAACCAGGAGACTTCGGTCTGGGATTCAAACCAGAAGACTTC
TGGTGGGATTCAAGCCAGGAGACTTCTGCTGGGATTTGAACCAGGAGGCTTCTGCATATTCGAACCAGGAGACTTCTGCATT
CTAGCCTGATGTTCTATCCATTGAGCCAGCCTGATGTTGTACCCGTTGAGCCTCCCAGGGTGGGATTCAAACCAGGAGACTTC
GCATCCCAGCCTGATGTTCATGTGCTGGGATTTGAACCAGGAGATCTGGGCTGGGATTCAAACCAGAAGACTTCTGCATCCC
AGCCTGATGTTCTAGCCATTCTGCATCCCAGCCTGATGTTCTAGCTTCTGTATCCCAGCCTGATGTTCTACCCAGGGTGGGATT
CAAACCAGGAGACTTCTGGGATTTGAACCAGGAGGCTTCTGCATATTCGAACCAGGAGACTTCTGCATTCTAGCCTGATGTTC
TATCCATTGAGCCAGCCTGATGTTGTACCCGTTGAGCCTTAAGCCTGATGTTCTACCCATTGAGCAACCTGCCTGATGTTCTA
CACATTGAGCCACCAGTGAACCAGGGACCAGGAGGCTTCTGCCTGGGAGACTTCTGCATCCCAGCCTGTTGCTCTGCTTAAG
CTAATAAACAGAATGTCCATTGGTTTGCAAGTATCAAATTTTTTTCTTCTTTGTAACTATTACATTCTACACCATCATGGTCTC
TTACATTTATAATTTTGTGGGTAAAACTGCTACACTGGCATAATCAACTTATGAATTCTCGAGGGCGCACATAACAGTGCTCT
TAAAGCAAGCCTTTTGATCACCATATTGACATGGTACCTGGTAATTTATGAGAGAGCTCAGAGCAGCGTGTGTGTCTCCTGCG
ATTTGCATCTGAGATGGTGCCCTGTAATTTATTCACATCATTTCATCCCAGAAAACAAACGCGTACGAATAAACACAAAGCA
CTGTCAAGGAAGAAGTCCAATTAGTGGTTTTTGCGTTATTAAAGGAAGCACAGATTTAGGATTTTTTTAAGTATTACTCCTT
GTAACCGTCTCTCTCTCTCTCTGTTCAGAGCGGTTGTTTCTAATGAGAGTTGATGCTTCAAGAACAGACACGGGTTGACAGTG
GGAAGGGGGGGGGGGCAGTCATGTAATTCCTCTCTGCTAAGTGCTGGACATGAAGGGTCACAATGAAAACATGCAATCACC
ATGTTTGCAATGCATCAATGCATCAGACTATTTGAAAGGTGTACGTTGGGGATGCAGCACTTCAAAAAGTCTCTCAGTTCAGT
TTGTATTGCGGTTTATGGGACACGGTTTGTAGTCTGGTGTGGTTTGTATTCAATATAATTGCTAAGCATTAACTAGCTTTTGAT
TGACTGTAACTCTCACATGGGTGATATCCACACAGAAAATTAATTTAGATCAATTTTTGCAACTACGTTAGCTGGTTTAAAAA
ATTATTCAAAGTAGAGATCGCTATGAGTAAAATGACTAATCATGACTGTCAGAACAATAGTTCGGTTACGTGTACTGAACCA
TGGTTGCCAAACTCAAAGGTTTCTTTTTTTCCGAGGAACCATTGAATCCCTAGTATACACAAAGGCCTTGATCTCCTCCAGGG
GAGAGATGTTGTTTTTTTTTTGGTCGATGTCTGTGTCAAAAGAAGGAAAGATAAACTGGGCTTGACGGATGTTGTATTTGACA
AGCTGCCCTGAACGGCTAACAGACAAGCCTGTGTGTTGGCAGTGATGCTGTGTGACCCCCATGATTGCGTACCCTGCGGCAT
CCCTGATGCCTTTCTGAGACAATTGGGTGGACACAGGGGGTGGGGGGGCTTGGAGGGGTGAGCCGCCTACTCTTCAATCACA
AGAGATTAAATTTTCAATCAGTTTTCCCCAATGAGAGAAACTTATCGTTTTTCAAGGCCCAAGCAGACCACATGCCCCTGCCC
TCACGCACATTAGCAGACTGCCTGAGGGTTCTGCCTCCCGAACAGTGTCATGCTTCACATGCTATTCAGCAATCTTATTAAAA
CACAACTCTCACCAAAGCCGGATGGACAGAGAAGACACAACCTGCTGCTGGTAGTCCTGCTGCCTTAGAACATGGAGAGAG
CAGCCCTCACACACAATGGTTTTAGTAACACAGCTTACGAACAAACACATACCTGCAGTGTATGAGCCTTACCTCCATGGTG
GCTGTAGGAACGAGGAAGAGTAAAAGTCATTCAGAAAAAATTACACTCACCAAATCCACACATGAAAAAACTTGGTAAATT
GTGTGTAAGTTGAGGAGTGAAACTAAAGGTCAGAGAGAAAGCCTGCTGATGTAGTGCAACATAACAGGAACATGGCGGCAA
AATAATAAGGAAAAGCGAATGTATCAACGGGCGTGCAGAGGTGAAGATAAAGTGTGAAATGCTGACTGCCACAAGGCCTTT
CTGCTGTCAGAAGGGTCATATAGAGGTCTTTTTTTTTCTTTTCTTCTCCCCATTTGTATCAGCTGTGTCTGATAAGCACAGGTG
TGTGAGCTTTCATCTTAAAAAAAGTCACCATGCTCCTAACCACTTGCTATAGGAGAGGGCTAATCTTTATTATTAAGAACACA
CGACAGTTGAAAAAGCAAGCAATTAGGTATTTAATGACCCCCACAGACATTATTGAATGCTGTAATATTAGGAATAAGCGTT
TGATCAAATCTTTAAAAAGACTGTTTGTACAAGTGTGGTCTGCAAGTCTGAATAATGCTGCTCTGTTATGGTGAGCTTGTCGT
CATGGGGATGCTATGTCATAACGTGAAAGCAGTGCTAACGTGCTCAGACTCTAGGAGAGTTGCTCCCTGAACAGCTTCTGTC
TTTTTTCTAGTCCTTTCAATAACATGTTCAGAAATGGCGCAGTGTCACATGTATGCAAATTATTATTATTTGTTCCCTACTAAA
ACTAAGCAGTTTAAAATTTTCAGTCACCGATGTATCGTTAGTTTGGAACAAAATGGGGGGGGGTTGTTTCTGATATGAGAGA
AGTAAATAAGAAATATGTGGGGGGTATTTAGTTAGTACTGCAGAACCATGATCTTAGCTCACCAATGTCCGCCTTCTCGGCG
ACAGGCTGTTTGAGACCCACCCGGAATGCAAAGACGTCTTCTTCCTGTTTCGTGACGTGGAGGACCTGGAGCGGCTGCGTAC
CAGCAGGGAGCTGCGAGTCCACGGCCTCCGGTTGGTCACCCCCCCCCCCCCNNNNNNNNNNCCAGCCCCAATTCTTAACACC
TGTACTTTCCATCTCTGGGTGAAAACCAAGTGACAAATCTGTCTCAGTGGTGCACAGTCAATGTCATAGCTGAGTGAGTTTTC
ATGGTCAAAGGCACCATGATAACTTAAACTTTAAGTAATTATAACCTGCAGCCTCGTGTCTATTTTATTCAAGAATAATATTC
CAATATTACCTATTATTATTATTAT

>GbX2a_Chitala_NODE_507691_length_4177_cov_10.785253

GGTTGTGTCTGAGGCCTCCGGCTCTATTGAGGGACGGATTTTCAATTTTCCTATATAGACTCCTTCACTCTGCTCTCAAACCAC
CTATCCTTCTCTATCTCAAATGTGCACATTATTGTCTTCAAAGCAGTGTCCTGTTTCTTTTAGATGAAGGTACTTGAAATTCCT
TCCTTGTTCAGCTAACAAGCCTATCGGATTTGAGTGCTGGAAACCAACCTGGAGGATAAATATGGGGTCCCTGTTCAATGAT
AAATATGGGGTCCTTGACCAATTTTAACTCAGGCGAAGAAGCTACTTGGATAAGCAGCGACACAACTTGGATGAGTAGAGA
AACGTTCCTGCTTGGATGAGCAGTGAAATGTAAACTTGGATGAGTAGAGAAACGTTCCTGCTTAAAGTGAAACGTTCCTATC
AAGAAGAAAGCAGTCCAGTTGACATGATTCAACTTCCAGACATTATGTGCAACTGTTTCATTGCCAAGTAAAACATGTACTG
ATTTGCCAAATAAGAAAGCTTGTCTGATAAATGACAGTAGATGAGAAAGCAACTTAATCTCAAATATAGCAGCTATTCAAAT
TCGAAACGTTTTAACATAAGTACATCCATTGAATTATAATAATATGAATGACATAAAAGGGACTGATGTTAAAATACTGATG

ATTCACTACGTGAATTAAGTCTGCTGTACAGACTGGCCAATAAAAAACTCTCAGTGATGCATGAGGTAAAGGAAAGACTCAG
TTCTTTTCCTGTAAACATATAATTTCAAAGGATAAGATGGAAACATTGTCTGTTGTCTGAAAGGATTTTAGAATTGGTGGGTC
AGAGAGTAGTTCCGTTAGCTTCAAGGAATTGTCACATTTGATTTCAGTCCCAAGAAAAAGCACACAATGTTGCGGTATAGTT
AATCTACACCGCCTGCTGTTGCATCTGTAAGGACAGGGAAAATAAGCAGCGTTACAAGCGCACCCACCCTAGGGGCACTTCT
GTTAGTGTACAGGCAAGTCCAGGGGCCTCTTCGTTTGGCAGAAGGAGGTTGGTTTCAGACCCATCAAATCTCTGCCACACTG
GGGCACTAGCAGTGCAGGCTGTGGTCACATGGACTTGTACACGTGGCTCTGTGTGAATGCACACAGTTACTGACACGCACAC
ACACACACACACACACTGGCAGACATACCAGAACAGATTTTTCAACAGAGCTGAGAGGCCTACCATCTGTGAAAACTGAAA
AGAGTAATGTGGAATGTTGTTGAAGCATGTAACGTCTGGACCCGGGACCACAGAATGCTTTGGCCCTCCAGCATGGAAAAGA
TATAAAACAGGGGCTCTTTTGTGTGTCCTGGACCCCTTTGTCAATCTGGTAAAGTTTACACATCTCCTTCTCGGAATGTGTTTC
AATGCGTAAAATAAAATCGGAATTTTTAGTCAGTGAAAATAACGATAATTTTTCCCTCTCCAAACTCAGGTACCCCAGGTTGG
GAACCAGCGGTCTAGGCCGAGTTTGAAACAGACCATGGACTGTGTCAGGCATGTGGCGTGCTCAGTACTGTCCCCCCCCCGT
ACCGCTGCAGGGTGATGTCCTTCATCGAGAAAAGCGTGGCCAGACTGGACCAACTGGAGGCCCTGGATGAGCTCGCCATCGA
GCTGGGGAGGAGCCACTATCGTTACAACGCCCCCCCCAAGTATTACGGGGTAGGACAGAGACACGACCGTGAACACACCTG
GACACTTCTAGGTTCTTCGTGTGACACACTACACTGAAATAAAGAGTGGTGTCAGTCAGGGGTCACTTGTACAAGTGCGTAG
GAGTGTGTTTTATATTGAGGGGACAGAGCTCCTCAATGTCATAAAAGTAAATAAACATATCAAAGTCTGAGACATTATCCCA
CCTGTTTTGTCCTCTCATACATCTGACACTTTTAAAATCTTTGCCTGGGTGAAACAGTCCAGCAGCACCAGTTATGTGAGAGA
TCAGCCACACTAATGTTCACAATATGCATAGTTAATTTTAGTGTAAGGTACTTTAAAACTGCATGCATGTTCATTGCCCAGGA
CAGAAAAGCACTTGGGAATAAGACACCAGTCACGGCCGCACAGTTCCAACTGGCACTGTGGAATACTATGAAGGTGATGTC
AGTTCACACCTAAAACCAAGGGGACAGCGCTGTTAGCGTTTTCTTTAGTTATGCCCTCTAGTGGTGACTACACTACATACTAA
CTTGTGAAACTGCCAGCAGTGACTGATGCCCTCCTTCAAGACAATCCTCTGTGATTATTCAGTATGTGGGGGCGGAGTTTATC
AGCGTTGTCCAGCCAATCCTGAAGGAAAATTGGACTCCAGACCTGGAGGAGGCTTGGAAGGTACAGATTTACAGCATTTAAA
TTATTGCAAATGTCTTGATTTTTCGCTTTTGTTGAAATGTTATAAAAGTGTAAAATGTATAAGCAATCNNNNNNNNNNCAGAT
AAAGGTACATTTTTTTGCCACTTGAAAAAAATTATGCCACAAGTAGCAGGAAAGTGCAATTGAGATTTAAATTGTAGTTCTAT
GACAATCAAAAGGTGAGCCTTTTGAAACCACAAAAACATCTAAAGCCTCTATGCTTGTTTTATTCAAATATGTTGTGACAGG
AAATCACAATTCAAATCTCTGATATGACCTGGTTTAAGTGGGTTTTATATTTTGAGTAACTGTGTAGTGATTTAATCACAAGG
CATGGTGCGTGTAAAAAATCCCCATGCTGAACACAGGCAATGGAACATGGTCTTGTATTGAAGTGCCACCCCGTAAGTTCAC
CGCACCTCTCTCATGAACACGTCGGTTTTGCCACCGCTGACACGTGACGGCACACATGCAGACGGGGGAGCCGGAGCTGCAC
GTCAGTATTCTAACAGCCAACCTGCTGCACAGCTGGACCAGGCCAAGGTGCAAGATTAATTACCGAACAGATTAACAGGCGA
GCTAGGGGGTGGCACTGACAGGCAAATTAGTGAGCAACTCTGGTTGTTAGCTAGAGGTACAGATGTGCGTGTAATTAATGAA
CAGCAGTCCTATGAGGACGAATCATAGGACAGACTCACCTCTGGCAACCAGTGATGCTGCACTGCCACGGGCAAAGTGTAA
AACACCTTTGTGGGCACAAACGTTCTCACCCCTCCTCCCCCACCACACACAGAACATGTTCCTGTACATCTCCAGACTGATGA
CGGAAGGTTACCAGGAAGAGGAGAGGCGTATCCGCAACAGCCTGGCAGCGTCTCCACGGGAACGACCCGAGAAGGCCAAC
ACCGCCATTTAGAAAGAGCATGGAGAGCACAAAGGCCCTCTGGCAACAAATGTCATGTGCCACCTACCCATGTTCAGTGTGT
CATTTTCGTTTTGTGTTGAATGAAAACTCTACTTCCTCTTGACAACAGAATTAATTAAATTTCAAAAGCGTACACCAAGCCGC
TCTTTAGCGTATCTACGAACGTACACACAAGCAGTCAGAGCTCCAGTGAGGCAGTACGGTCTGTAGAGAATCGCTACTCTCT
GTAGCTTTGGTTGTGATTGTGTATGAACAGCACTTGGCGAACAGTTCAACATCCAACGCTATTAAAAATTTATATAACCATAC
ATGCAGAAACGAACAGCCCGAAAGCCGATTTGTTTGTGATCGAGCCCAACATAAACCCAACAGCCAGTTGAGTAACACAGC
TCCATCCAGCACAGTTTCTGTAGAACCTCAGCCATTTGGCTTCGCATTGGGCAGCAGATCTGTCAGGTCACAGCTCCCACCCT
CGGGCCGCTATTGATTACCCACAATGCTCTCCACCGGTTGATGGCAGCAGGACTCCAAACAGCACCAGTTACCAGTACTTAC
TTCCTAAAGAAATATGCCAATAGAAATCAATAAACTGTGAAGTTAAACAGCTGCCACCACGAGTTTCAACTATGATTGATGG
CTAGAAATAAACATGACACATACATCAGCAGAGTTACCAATTTATTATTCCCCCCCTTCACAAAGGTCAAACAAAAACAAAC
CAAACCAGTATTGTGGGAAGACAACACCCGAGGATTAGGGGGAGAATGTTCCCATTGTATAGACAGCAGTAGGCTGATCTTC
TCATCTCAAAAACTAAACATTTTATTCTCCAATTTTCATGAAATGTATTTAAAAAACATGGCAATTTGAAAAATAAAAATTAA
AAAAAAAAA

>GbX2a_Chitala_Full_Coding Sequence

CTCGTGGAAGGTCATCCAGGAAGATATCGCTAAAGTTGGGATTATAATGTTTGTCAGGCTGTTTGAGACCCACCCGGAATGC
AAAGACGTCTTCTTCCTGTTTCGTGACGTGGAGGACCTGGAGCGGCTGCGTACCAGCAGGGAGCTGCGAGTCCACGGCCTCC
GGTGATGTCCTTCATCGAGAAAAGCGTGGCCAGACTGGACCAACTGGAGGCCCTGGATGAGCTCGCCATCGAGCTGGGGAG
GAGCCACTATCGTTACAACGCCCCCCCCAAGTAGCGGAGTTTATCAGCGTTGTCCAGCCAATCCTGAAGGAAAATTGGACTC
CAGACCTGGAGGAGGCTTGGAAGG
